# A transcriptomics-based drug repositioning approach to identify drugs with similar activities for the treatment of muscle pathologies in spinal muscular atrophy (SMA) models

**DOI:** 10.1093/hmg/ddad192

**Published:** 2023-11-08

**Authors:** Joseph M Hoolachan, Eve McCallion, Emma R Sutton, Özge Çetin, Paloma Pacheco-Torres, Maria Dimitriadi, Suat Sari, Gavin J Miller, Magnus Okoh, Lisa M Walter, Peter Claus, Matthew J A Wood, Daniel P Tonge, Melissa Bowerman

**Affiliations:** School of Medicine, David Weatherall Building, Keele University, Staffordshire, ST5 5BG, United Kingdom; School of Medicine, David Weatherall Building, Keele University, Staffordshire, ST5 5BG, United Kingdom; School of Medicine, David Weatherall Building, Keele University, Staffordshire, ST5 5BG, United Kingdom; School of Medicine, David Weatherall Building, Keele University, Staffordshire, ST5 5BG, United Kingdom; School of Life and Medical Sciences, University of Hertfordshire, Hatfield, Hertfordshire, AL910 9AB, United Kingdom; School of Life and Medical Sciences, University of Hertfordshire, Hatfield, Hertfordshire, AL910 9AB, United Kingdom; Department of Pharmaceutical Chemistry, Hacettepe University, Ankara, 06100, Turkey; School of Chemical and Physical Sciences, Lennard-Jones Building, Keele University, Staffordshire, ST5 5BG, United Kingdom; School of Chemical and Physical Sciences, Lennard-Jones Building, Keele University, Staffordshire, ST5 5BG, United Kingdom; Centre for Glycoscience, Keele University, Staffordshire, ST5 5BG, United Kingdom; School of Medicine, David Weatherall Building, Keele University, Staffordshire, ST5 5BG, United Kingdom; SMATHERIA gGmbH – Non-Profit Biomedical Research Institute, Feodor-Lynen-Straße 31, 30625, Hannover, Germany; Centre of Systems Neuroscience (ZSN), Hannover Medical School, Bünteweg 2, 30559, Hannover, Germany; SMATHERIA gGmbH – Non-Profit Biomedical Research Institute, Feodor-Lynen-Straße 31, 30625, Hannover, Germany; Centre of Systems Neuroscience (ZSN), Hannover Medical School, Bünteweg 2, 30559, Hannover, Germany; Department of Paediatrics, University of Oxford, Level 2, Children's Hospital, John Radcliffe, Headington Oxford, OX3 9DU, United Kingdom; School of Life Sciences, Huxley Building, Keele University, Staffordshire ST5 5BG, United Kingdom; School of Medicine, David Weatherall Building, Keele University, Staffordshire, ST5 5BG, United Kingdom; Wolfson Centre for Inherited Neuromuscular Disease, RJAH Orthopaedic Hospital, Oswestry, SY10 7AG, United Kingdom

**Keywords:** spinal muscular atrophy, skeletal muscle, transcriptomics, drug repurposing, animal models

## Abstract

Spinal muscular atrophy (SMA) is a genetic neuromuscular disorder caused by the reduction of survival of motor neuron (SMN) protein levels. Although three SMN-augmentation therapies are clinically approved that significantly slow down disease progression, they are unfortunately not cures. Thus, complementary SMN-independent therapies that can target key SMA pathologies and that can support the clinically approved SMN-dependent drugs are the forefront of therapeutic development. We have previously demonstrated that prednisolone, a synthetic glucocorticoid (GC) improved muscle health and survival in severe *Smn^−/−^;SMN2* and intermediate *Smn^2B/−^* SMA mice. However, long-term administration of prednisolone can promote myopathy. We thus wanted to identify genes and pathways targeted by prednisolone in skeletal muscle to discover clinically approved drugs that are predicted to emulate prednisolone’s activities. Using an RNA-sequencing, bioinformatics, and drug repositioning pipeline on skeletal muscle from symptomatic prednisolone-treated and untreated *Smn^−/−^; SMN2* SMA and *Smn^+/−^; SMN2* healthy mice, we identified molecular targets linked to prednisolone’s ameliorative effects and a list of 580 drug candidates with similar predicted activities. Two of these candidates, metformin and oxandrolone, were further investigated in SMA cellular and animal models, which highlighted that these compounds do not have the same ameliorative effects on SMA phenotypes as prednisolone; however, a number of other important drug targets remain. Overall, our work further supports the usefulness of prednisolone’s potential as a second-generation therapy for SMA, identifies a list of potential SMA drug treatments and highlights improvements for future transcriptomic-based drug repositioning studies in SMA.

## Introduction

Spinal muscular atrophy (SMA) is a heterogenous autosomal recessive neuromuscular disorder (NMD) characterized by motor neuron degeneration alongside progressive muscle atrophy and weakness [1]. Being the leading monogenic cause of infant mortality [2], around 96% of SMA cases are mapped to homozygous loss-of-function and deletion mutations in the *survival of motor neuron 1* (*SMN1*) gene [3, 4], which ubiquitously expresses SMN, a protein that current and ongoing research has linked to diverse housekeeping and tissue-specific cellular functions [5–7]. Although complete SMN loss is embryonic lethal in most organisms [8], humans can overcome the complete loss of the *SMN1* gene due to incomplete rescue by the homologous *SMN2* gene [9, 10]. In essence, the presence of a single nucleotide mutation in *SMN2* promotes exon 7 alternative splicing that limits full length SMN (FL-SMN) expression in this gene to 10% [11]. Consequently, the limited FL-SMN expression makes *SMN2* gene copy number an important disease modifier, impacting SMA type and severity [12].

In recent years, novel SMN restorative SMA treatments have emerged that either increase FL-*SMN2* expression by an anti-sense oligonucleotide (ASO) (Nusinersen marketed as (Spinraza®)) [13, 14] or a small molecule (Evrysdi®) [15, 16] or promote exogenous FL-*SMN1* expression by an adeno-associated virus 9 (AAV-9) delivery system (Zolgensma®) [17, 18]. Despite the significant increased life expectancy and improved quality of life associated with these therapies [14, 16, 18, 19], they are not cures and their efficacy is dependent upon early intervention [20].

Thus, additional SMN-independent therapies that target affected tissues such as muscle are needed to further enhance and support the benefits of SMN-dependent treatments [21]. Indeed, pre-clinical studies and primary patient data have reported innate muscular defects in SMA, which include myogenesis [22], regeneration [23], contraction [24, 25], regulation [25], growth [26], and metabolism [27], highlighting skeletal muscle as a primary therapeutic target. Although two novel skeletal muscle-specific SMA therapies, Apitegromab™ [28] (ClinicalTrials.gov ID: NCT03921528) and Reldesemtiv™ [29] (ClinicalTrials.gov ID: NCT02644668), are showing progress in clinical trials, the high expenses involved in novel drug research and development (R&D) [30] alongside the costs of the three clinically approved SMN-dependent therapies may lead to elevated prices for combinatorial treatments [31], thus further widening the accessibility gap for SMA patients.

A useful alternative for ensuring accessibility of SMN-independent treatments for all SMA patients would be the identification of cost-effective generic drugs via drug repositioning, a strategy aimed at finding new therapeutic activities for existing pharmacological compounds [32]. One such example is prednisolone, a synthetic glucocorticoid (GC) administered to relieve muscle inflammation in Duchenne muscular dystrophy (DMD) patients [33, 34]. Interestingly, evidence also emerged that short-term prednisolone treatment additionally conferred ergogenic muscle benefits in DMD patients [35, 36], which was also validated in the *mdx* mouse model of DMD [37, 38].

Although GCs are not used for the treatment of SMA patients, prednisolone is administered for a short period (~30 days, 1 mg/kg) to alleviate the immunological adverse effects of Zolgensma® [18]. However, given prednisolone’s potential muscle benefits, we have previously investigated and demonstrated that prednisolone treatment (5 mg/kg, every other day) in severe *Smn^−/−^;SMN2* and intermediate *Smn^2B/−^* SMA mice improved survival, weight and neuromuscular phenotype [27]. Although the study was aimed at investigating prednisolone’s activity on the GC-Krüppel-Like-Factor-15 (KLF15) pathway in SMA skeletal muscle, synergistic muscle improvement was also observed in prednisolone-treated *Smn^−/−^;SMN2* SMA mice overexpressing Klf15 specifically in skeletal muscle [27], suggesting that prednisolone may act on SMA skeletal muscle via numerous effectors and pathways.

**Figure 1:**
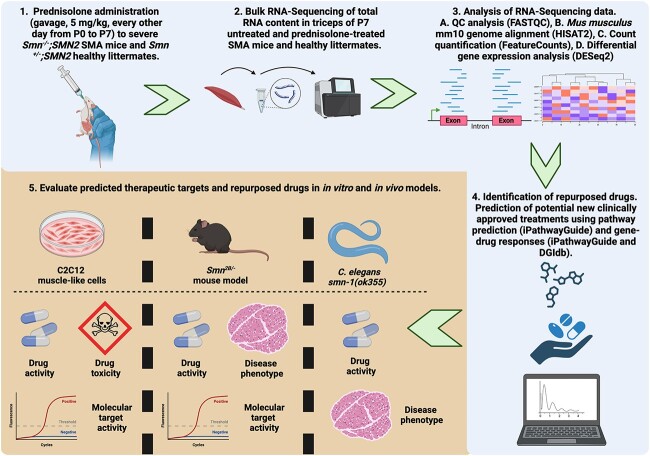
Graphical representation of the study aimed at using a transcriptomics-based drug repositioning strategy to predict and validate drug candidates that emulate prednisolone’s activity in SMA skeletal muscle. (1) 5 mg/kg prednisolone was administered was administered to *Smn^−/−^;SMN2* SMA mice and *Smn^+/−^;SMN2* healthy control animals every other day from post-natal day (P) 0 to P7. (2) Total RNA was extracted from the triceps of prednisolone- and untreated SMA mice and *Smn^+/−^;SMN2* healthy control animals and underwent library preparation for next generation sequencing against *Mus musculus* mm10 genome using Illumina NextSeq 550. (3) The RNA-sequencing (RNA-Seq) reads initially underwent QC analysis via FASTQC prior to a transcriptomics pipeline of mm10 *M. musculus* genome alignment (HISAT2), read count quantification (FeatureCounts) and differential gene expression analysis (DESeq2) to generate differentially expressed genes.(4) The differential gene expression patterns between prednisolone-treated vs untreated *Smn^−/−^;SMN2* SMA triceps was used to identify significant pathways targeted by prednisolone (iPathwayGuide). Both differentially expressed genes and significant pathways were used to predict clinically approved drug candidates from online databases (iPathwayGuide, KEGG drugs and drug gene interaction database (DGIdb)) that emulate prednisolone’s activity in skeletal muscle of SMA mice. (5) The activity and safety of predicted drug candidates of interest and their target genes were then assessed in both *in vivo* (*Smn^2B/−^* SMA mice and *Caenorhabditis elegans smn-1 (ok355)*) and *in vitro* (C2C12 muscle-like cells) models. This figure was generated in BioRender.

**Figure 2:**
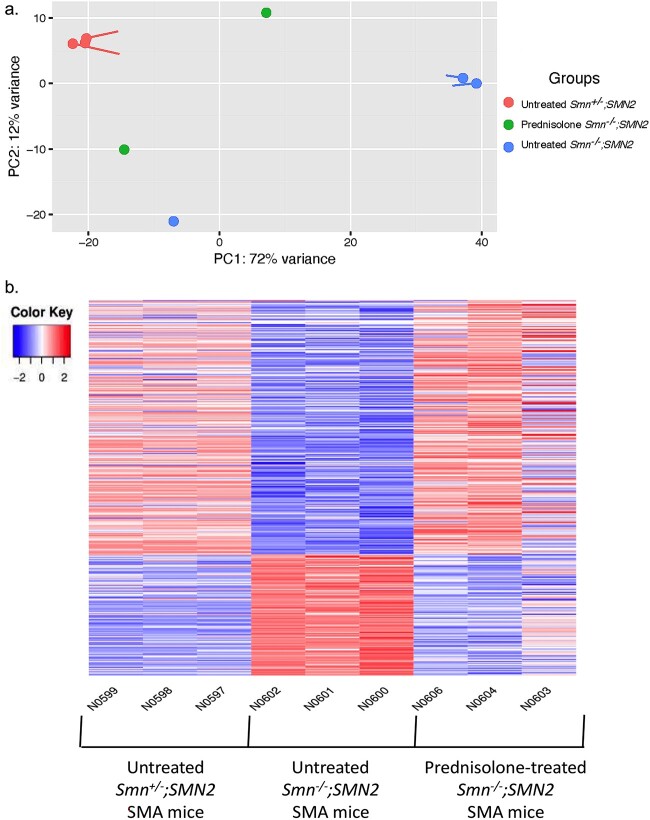
Prednisolone treatment normalizes a subset of genes in severe *Smn^−/−^;SMN2* SMA mice to healthy levels observed in untreated *Smn^+/−^;SMN2* mice. *Smn^−/−^;SMN2* SMA and *Smn^+/−^;SMN2* healthy mice received prednisolone treatment (5 mg/kg gavage every 2 days) from P0. The triceps was harvested from P7 untreated and prednisolone-treated *Smn^−/−^;SMN2* SMA and *Smn^+/−^;SMN2* healthy mice for RNA isolation and library preparation for RNA-sequencing. Differential gene expression analysis was performed by DESeq2 v2.11.40.2 with study design set to “condition and “treatment”. (a) Principal component analysis based on transcriptomic profiles between P7 untreated *Smn^+/−^;SMN2* (n = 3), prednisolone-treated *Smn^−/−^;SMN2* (n = 2) and untreated *Smn^−/−^;SMN2* (n = 3) mice. (b) Heatmap of the transcriptomic expression profiles (Log2FC > 0.6; FDR < 0.05) between P7 untreated *Smn^+/−^;SMN2* (n = 3, left), untreated *Smn^−/−^;SMN2* (n = 3, centre) and prednisolone-treated *Smn^−/−^;SMN2* (n = 3, right) mice.

Despite the benefits observed, our study did not evaluate prednisolone’s long-term effects [18, 27]. Given that chronic GC usage increases myopathy [35, 39], it is unclear whether long-term prednisolone treatments would be similarly detrimental in SMA muscle. Furthermore, the rapid onset and progression of disease in SMA mouse models (1–3 weeks on average) [9, 40, 41] does not allow sufficient comparison of intermittent vs chronic studies.

Thus, in this study, we used a transcriptomics and drug repositioning pipeline based on our previously published experimental paradigm [42] to uncover the genes and pathways restored by prednisolone in skeletal muscle of SMA mice and identify existing non-GC drugs predicted to have similar activities (Fig. 1). Our study uncovered that prednisolone restored pathways linked to growth, metabolism, and regulation in SMA skeletal muscle and identified 20 leading commercially available non-GC drugs predicted to emulate its action. Based on oral bioavailability and evidence of safety treatment in children, we selected and validated metformin and oxandrolone in SMA cellular and animal models (Fig. 1). Although, both metformin and oxandrolone improved neuromuscular activity in the *Caenorhabditis elegans (C. elegans)* model for severe SMA, we found that higher metformin doses reduced survival in the *Smn^2B/−^* SMA mouse model. On the other hand, oxandrolone treatment partially improved survival in *Smn^2B/−^* SMA mice, albeit not to the same extent as prednisolone [27].

Nevertheless, our study computationally uncovered new mechanisms behind prednisolone’s beneficial activity in SMA muscle, identified numerous potential SMA muscle-specific therapeutic candidates and highlighted the importance of transcriptomic-based drug repositioning for SMN-independent drug discovery.

## Results

### Prednisolone restores the expression of a large subset of genes involved in canonical skeletal muscle pathways in SMA mice

As described in an earlier study, we have previously demonstrated that treating SMA mice with prednisolone significantly improved several disease phenotypes, including survival, weight, and muscle health [27]. To have a more in depth understanding of the impact of prednisolone on SMA skeletal muscle at a molecular level, we performed bulk RNA-sequencing (RNA-Seq) on skeletal muscle of untreated and prednisolone-treated *Smn^−/−^;SMN2* SMA and *Smn^+/−^;SMN2* healthy mice. Specifically, we administered prednisolone (5 mg/kg, gavage, every 2 days) starting from post-natal day (P) 0 until P7 to *Smn^−/−^;SMN2* SMA and *Smn^+/−^;SMN2* healthy mice [27]. Triceps were harvested from P7 prednisolone-treated and untreated mice for RNA-Seq via Illumina NextSeq550 and a HISAT2-FeatureCounts-DESeq2 pipeline against a *Mus Musculus* mm10 genome for parameters of “condition” and “treatment” (Fig. S1).

Initially, our principal component analysis (PCA) revealed distinct clusters of untreated *Smn^−/−^;SMN2* SMA and untreated *Smn^+/−^;SMN2* healthy littermates, with prednisolone-treated *Smn^−/−^;SMN2* SMA mice falling between the aforementioned groups (Fig. 2a). Importantly, we found that prednisolone treatment restored the expression of 1361 genes in *Smn^−/−^;SMN2* SMA mice to levels similarly observed in untreated *Smn^+/−^;SMN2* healthy mice (Fig. 2b; Tables S1–S3).

Next, we determined the biological pathways associated with differentially expressed genes (DEGs) in prednisolone-treated *Smn^−/−^;SMN2* SMA mice compared to untreated *Smn^−/−^;SMN2* SMA mice. Using iPathwayGuide, we identified that 3056 significant DEGs (Log2 fold change (Log2 FC) > 0.6, false discovery rate (FDR) < 0.05) (Table S2) were targeted by prednisolone in the skeletal muscle of *Smn^−/−^;SMN2* SMA mice when compared to untreated *Smn^−/−^;SMN2* SMA mice and associated with 28 significant KEGG pathways (*P* < 0.05) (Table 1). Interestingly, these prednisolone-targeted pathways are closely associated with important skeletal muscle processes such as metabolism, atrophy and regulatory function, alongside previous associations with SMA-related pathways such as FoxO signalling [43], p53 signalling [44], AMPK signalling [45], mitophagy [46], circadian rhythm [47], PPAR signalling [48] and autophagy [49] (Table 1). An additional gene ontology (GO) analysis also revealed similar skeletal muscle biological processes associated with the DEGs in prednisolone-treated *Smn^−/−^;SMN2* SMA mice such as myotube differentiation, fatty acid oxidation, protein ubiquitination, sarcomere regulation, gluconeogenesis, and circadian rhythm (Table 2; Tables S4–S6).

Combined, our transcriptomics and pathway analyses suggest that prednisolone treatment attenuated muscle pathologies in SMA mice [27] by targeting key muscle metabolism, atrophy and regulatory pathways.

### Drug repositioning algorithms identify novel pharmacological compounds predicted to emulate prednisolone’s activity in skeletal muscle of SMA mice

As mentioned above, while prednisolone treatment significantly improves muscle health and overall disease progression in SMA mice, chronic use of prednisolone can negatively impact skeletal muscle [35, 39]. As such, we used the DEGs and associated KEGG pathways identified in prednisolone-treated *Smn^−/−^;SMN2* SMA mice to discover alternative drugs predicted to mimic prednisolone’s molecular effects in SMA skeletal muscle. Initially, we utilized the in-built integration of KEGG drugs database in iPathwayGuide [50] and the DGIdb v3.0 [51] database to initially reveal a total of 580 compounds (Tables S7–S10). To filter down our list, we focused on the drug compounds 1) that targeted > 5 prednisolone-targeted pathways or linked to upstream regulators, 2) were clinically approved and 3) were not associated with promotion of muscle-wasting (e.g. primary anti-cancer drugs [52]), leaving a total of 20 potential candidates (Tables 3–4).

**Table 1:** KEGG pathways targeted in the skeletal muscle of symptomatic prednisolone-treated *Smn^−/−^;SMN2* SMA mice compared with untreated *Smn^−/−^;SMN2* SMA mice.

KEGG ID	Pathway name	#Genes (DE/All)	*P* value
04066	HIF-1 signalling pathway	30/91	0.001
05214	Glioma	25/71	0.002
04213	Longevity regulating pathway—multiple species	24/57	0.003
04068	FoxO signalling pathway	41/116	0.003
04713	Circadian entrainment	23/73	0.004
04211	Longevity regulating pathway	30/83	0.004
04115	p53 signalling pathway	25/67	0.006
00040	Pentose and glucuronate interconversions^a^	8/15	0.007
05010	Alzheimer disease	19/158	0.008
04152	AMPK signalling pathway	37/110	0.008
04080	Neuroactive ligand-receptor interaction	23/118	0.011
05418	Fluid shear stress and atherosclerosis	38/125	0.013
05223	Non-small cell lung cancer	22/64	0.014
05034	Alcoholism	25/104	0.014
04744	Phototransduction	4/9	0.015
05215	Prostate cancer^a^	28/88	0.016
04137	Mitophagy—animal	22/61	0.018
04659	Th17 cell differentiation	18/72	0.024
03030	DNA replication^a^	13/35	0.026
05031	Amphetamine addiction	13/49	0.028
05200	Pathways in cancer^a^	110/434	0.031
04710	Circadian rhythm	12/29	0.033
01210	2-Oxocarboxylic acid metabolism^a^	7/16	0.039
04140	Autophagy—animal	36/123	0.041
04372	Apelin signalling pathway	34/121	0.045
05226	Gastric cancer	36/123	0.048
00515	Mannose type O-glycan biosynthesis^a^	8/20	0.048
03320	PPAR signalling pathway	20/56	0.048

^a^Over-representation only.

**Table 2:** Top gene ontology biological process pathways targeted in the skeletal muscle of symptomatic prednisolone-treated *Smn^−/−^;SMN2* SMA mice compared with untreated *Smn^−/−^;SMN2* SMA mice.

GO ID	GO Name	#Genes (DE/All)	*P value* (Weight)
GO:0000055	ribosomal large subunit export from nucleus	8/8	0.0000054
GO:0010830	regulation of myotube differentiation	25/53	0.000088
GO:0034504	protein localization to nucleus	81/247	0.00011
GO:0046320	regulation of fatty acid oxidation	17/32	0.00028
GO:0048662	negative regulation of smooth muscle cell proliferation	20/44	0.00045
GO:0031062	positive regulation of histone methylation	17/35	0.00046
GO:0046854	phosphatidylinositol phosphorylation	17/36	0.0007
GO:0016239	positive regulation of macroautophagy	26/64	0.00113
GO:0000083	regulation of transcription involved in G1/S transition of mitotic cell cycle	8/12	0.00113
GO:0042594	response to starvation	49/148	0.00114
GO:0031398	positive regulation of protein ubiquitination	36/101	0.00115
GO:0090073	positive regulation of protein homodimerization activity	7/10	0.00157
GO:0048671	negative regulation of collateral sprouting	6/8	0.00208
GO:0007050	cell cycle arrest	40/121	0.00319
GO:1990830	cellular response to leukaemia inhibitory factor	37/110	0.00321
GO:1901215	negative regulation of neuron death	60/198	0.00378
GO:0007623	circadian rhythm	50/160	0.00378
GO:0061635	regulation of protein complex stability	6/9	0.00508
GO:0033137	negative regulation of peptidyl-serine phosphorylation	12/26	0.00533
GO:0032088	negative regulation of NF-kappaB transcription factor activity	22/59	0.00537
GO:0006094	gluconeogenesis	23/63	0.00604
GO:0001937	negative regulation of endothelial cell proliferation	15/36	0.00629
GO:0010715	regulation of extracellular matrix disassembly	7/12	0.00681
GO:0030240	skeletal muscle thin filament assembly	7/12	0.00681
GO:0035358	regulation of peroxisome proliferator activated receptor signalling pathway	7/12	0.00681

**Table 3:** Top 10 clinically approved drugs identified by KEGG database based on prednisolone-targeted KEGG pathways in symptomatic prednisolone-treated *Smn^−/−^;SMN2* SMA mice.

Pathways Targeted	KEGG ID	Compound	Bioavailability	Gene Targets
10	D03297	Mecasermin (genetical recombination) (JAN)	Subcutaneous	*Igf1r*
10	D04870	Mecasermin rinfabate (USAN/INN)	Intravenous	*Igf1r*
9	D09680	Teprotumumab (USAN/INN)	Intravenous	*Igf1r*
8	D04966	Metformin (USAN/INN)	Oral	*Prkag3*
8	D00944	Metformin hydrochloride (JP16/USP)	Oral	*Prkag3*
7	D01697	Colforsin daropate hydrochloride (JAN)	Oral	*Adcy1, Adcy6* *Adcy7*
6	D01146	Iguratimod (JAN/INN)	Oral	*Nfkb1*
5	D07058	Acamprosate (INN)	Oral	*Grin1, Grin2a, Grin2b, Grin2c, Grin2d*
5	D02754	Acitretin (USP/INN); Soriatane (TN)	Oral	*Rarb, Rxrb, Rxrg*
5	D00085	Insulin (JAN/USP)	Intravenous Subcutaneous	*Insr*

**Table 4:** Top 10 clinically approved drugs identified by DGIdb database based on prednisolone-targeted KEGG pathways in symptomatic prednisolone-treated *Smn^−/−^;SMN2* SMA mice.

Compound	Bioavailability	Gene Target
Testosterone	Busal Nasal Oral Topical Transdermal Subcutaneous	*Ar*
Oxandrolone	Oral	*Ar*
Nandrolone phenpropionate	Oral	*Ar*
Progesterone	Oral Vaginal Intramuscular	*Ers1*
Tibolone	Oral	*Ers1*
Cannabidiol	Oral Inhale	*Cnr1*
Insulin, neutral	Intravenous Intramuscular Subcutaneous	*Insr*
Celecoxib	Oral	*Pdpk1*
Tocilizumab	Intravenous Subcutaneous	*Il-6ra*
Sarilumab	Subcutaneous	*Il-6ra*

Interestingly, our combined *in silico* drug repositioning approach revealed a subset of candidates previously investigated in SMA such as celecoxib [53] (ClinicalTrials.gov ID: NCT02876094) and colforsin [54]. To further validate our bioinformatics strategy, we chose to continue our study with drugs not yet assessed for SMA, focusing on those previously used safely in young patients and orally bioavailable. With these criteria, we narrowed down our selection to metformin, a generic asymmetric dimethyl-biguanide type 2 diabetes mellitus (T2DM) drug [55] and oxandrolone, a synthetic anabolic steroid with a higher ratio of anabolic: androgynous effects for further study [56].

Thus, using a transcriptomics-based *in silico* drug repositioning platform, we were able to generate a list of clinically approved pharmacological compounds that are predicted to emulate prednisolone’s activity in skeletal muscle.

### Molecular docking simulations support the predicted drug-target interactions identified by the transcriptomics pipeline

**Figure 3:**
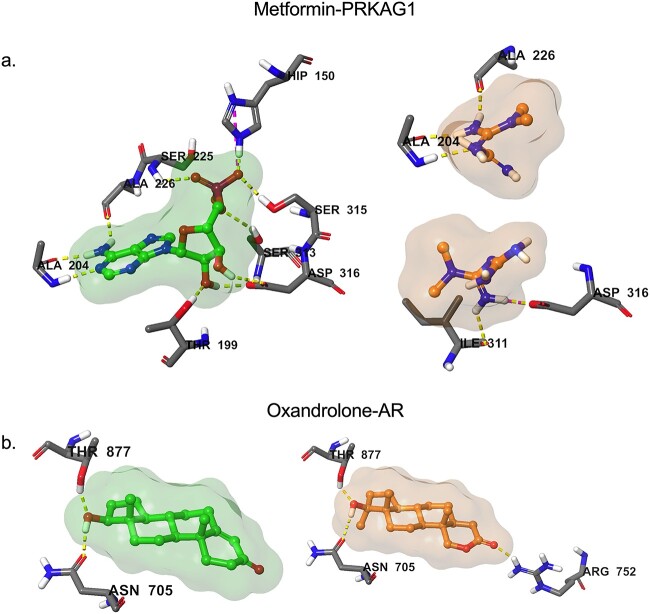
Molecular docking predicted binding of metformin and oxandrolone to their targets of interest. (a) Binding of the co-crystallized Prkag1 activator and the predicted alternative binding modes of metformin in Prkag1 active site. (b) Binding of the co-crystallized testosterone and the predicted binding of oxandrolone in the androgen receptor (AR) active site. Ligands are shown in colour ball-and-stick representation with molecular surface rendered, amino acid residues as sticks and binding interactions as colour dashed-lines.

Having selected to move forward with metformin and oxandrolone based on a combined bioinformatics and drug repurposing strategy, we performed molecular docking simulations to confirm that our drugs of interest indeed interacted with their predicted molecular targets (Prkag3 for metformin and androgen receptor (AR) for oxandrolone). In the case of metformin, no crystallographic data was available for Prkag3 (AMPK-γ3). We thus studied the interactions of metformin with Prkag1 (AMPK-γ1) (Fig. 3a), as both encode the AMPK-γ subunit, share 64% sequence homology and can form an AMPK trimeric complex with the same AMPK α2 and β2 subunits in human and mouse skeletal muscle [57]. Nevertheless, both metformin and oxandrolone demonstrated strong binding affinity to their targets of interest with metformin binding at Prkag1 key residues such as Ala 204, Ala 226 and Asp316 (Fig. 3a) and oxandrolone anchoring to the AR active sites via hydrogen bonds with Asn 705, Arg 752 and Thr 877 (Fig. 3b). Furthermore, docking studies across our larger list of orally bioavailable drug candidates (Tables 3–4) show that most of the drug molecules were predicted to fit in their respective target active sites, aligning well with known co-crystallized ligands (Fig. S2, Table S11). Thus, these molecular docking simulations support further investigating the therapeutic potential of the drug candidates identified via a transcriptomics and drug repurposing approach.

### Metformin’s primary predicted target gene *Prkag3* is dysregulated in skeletal muscle of both severe *Smn^−/−^;SMN2* and intermediate *Smn^2B/−^* SMA mice

As previously mentioned, metformin is an orally administered T2DM drug that we selected as one of the candidates to validate our bioinformatics-based drug repositioning approach. Importantly, metformin has over 60 years of clinical use with a well-known safety profile [55] and recorded administration in younger patients [58]. Furthermore, it has been previously repositioned and conferred ergogenic activities in muscular disorders such as DMD [59] and congenital muscular dystrophy type 1 A (CMDT1A) [60], highlighting its potential as a skeletal muscle therapy.

Our iPathwayGuide analysis predicted that metformin could emulate prednisolone’s targeting of the KEGG: 04068 FoxO signalling pathway (Fig. S3a). In particular, metformin was predicted to mimic prednisolone’s upregulation of *Prkag3*, which encodes for the AMPK-γ3 subunit of the predominant skeletal muscle AMPK-α2β2γ3 isoform complex [61] (Fig. S3). Furthermore, *Prkag3* upregulation was predicted to coherently downregulate the expression of *FoxO1*, *FoxO3* and *Foxo4* isoforms, while upregulating *FoxO6* (Fig. S2) supporting previous literature associating these FoxO isoforms with promotion of muscle atrophy [43, 62]. Importantly, the expression pattern of these genes in the prednisolone-treated *Smn^−/−^;SMN2* SMA mice were normalized to healthy *Smn^+/−^;SMN2* levels (Fig. S3c), supporting the usefulness of investigating metformin and these targets in SMA skeletal muscle.

**Figure 4:**
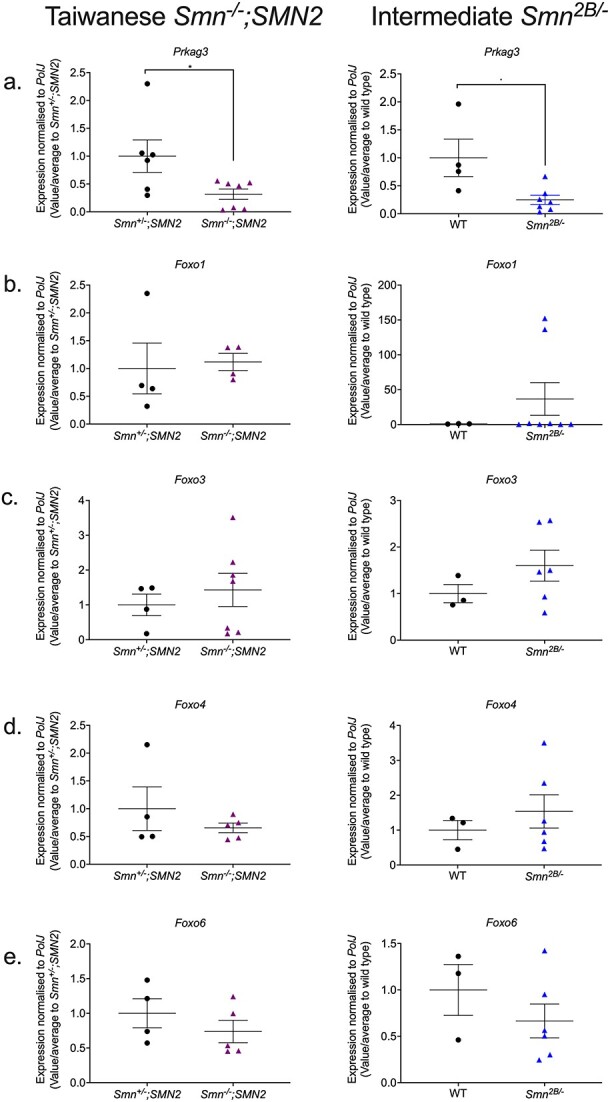
The metformin target gene, *Prkag3,* is significantly downregulated in the skeletal muscle of both symptomatic severe *Smn^−/−^;SMN2* and milder *Smn^2B/−^* SMA mice. qPCR analysis of mRNA levels for predicted metformin target genes (a) *Prkag3*, (b) *Foxo1*, (c) *Foxo3*, (d) *Foxo4* and (e). *Foxo6* in the harvested triceps of symptomatic untreated P7 Taiwanese *Smn^−/−^;SMN2* SMA mice and healthy *Smn^+/−^;SMN2* controls (left panel) and symptomatic untreated P19 intermediate *Smn^2B/−^* SMA mice and wild type (C57BL/6J background) controls (right panel). Data are shown as scatter plot represent as mean ± SEM error bars; n = 4–7 animals per experimental group, unpaired t-test, ^*^P < 0.05. *Smn^−/−^;SMN2 Prkag3*: *P* = 0.04; *Smn^−/−^;SMN2 Foxo1*: *P* = 0.82; *Smn^−/−^;SMN2 Foxo3*: *P* = 0.54; *Smn^−/−^;SMN2 Foxo4*: *P* = 0.37; *Smn^−/−^;SMN2 Foxo6*: *P* = 0.34; *Smn^2B/−^ Prkag3*: *P* = 0.02; *Smn^2B/−^ Foxo1*: *P* = 0.39; *Smn^2B/−^ Foxo3*: *P* = 0.27; *Smn^2B/−^ Foxo4*: *P* = 0.48; *Smn^2B/−^ Foxo6*: *P* = 0.33.

We thus measured the mRNA expression levels of *Prkag3* and *FoxO* isoforms in the triceps of both symptomatic P7 severe *Smn^−/−^;SMN2* and P19 milder *Smn^2B/−^* SMA mice alongside their respective healthy controls. We indeed observed that *Prkag3* levels were significantly downregulated in skeletal muscle of both SMA mouse models (Fig. 4a), supporting the bioinformatics data. However, none of the *FoxO* isoforms were significantly different between SMA mice and their healthy controls (Fig. 4b–e). Previous research also reported no significant upregulation of *FoxO* isoforms in P7 severe *Smn^−/−^;SMN2* SMA mice via qPCR [43]. Although another study observed a significant upregulation of FoxO isoforms in the hindlimb muscles of *Smn^2B/−^* SMA mice [43], the wide variation observed in our experimental cohort could be due to differential vulnerabilities to denervation-induced muscle atrophy between hindlimb and triceps muscle [63] that could impact FoxO expression levels. However, the fact that our qPCR data did not reflect the bioinformatics predictions may also be due to variability in our experimental cohorts, the sequencing depth coverage not being sufficiently conservative and/or intrinsic differences between RNA-Seq and primer-based qPCR approaches [64].

Overall, our qPCR experiments revealed that the primary metformin target *Prkag3* matched its bioinformatics prediction of being downregulated in both *Smn^−/−^;SMN2* and *Smn^2B/−^* SMA mice, suggesting that this gene may be involved in both severe and milder SMA pathologies and an appropriate therapeutic molecular target in SMA muscle.

### The predicted target genes for metformin are mostly Smn-independent in an SMA muscle cellular model

**Figure 5:**
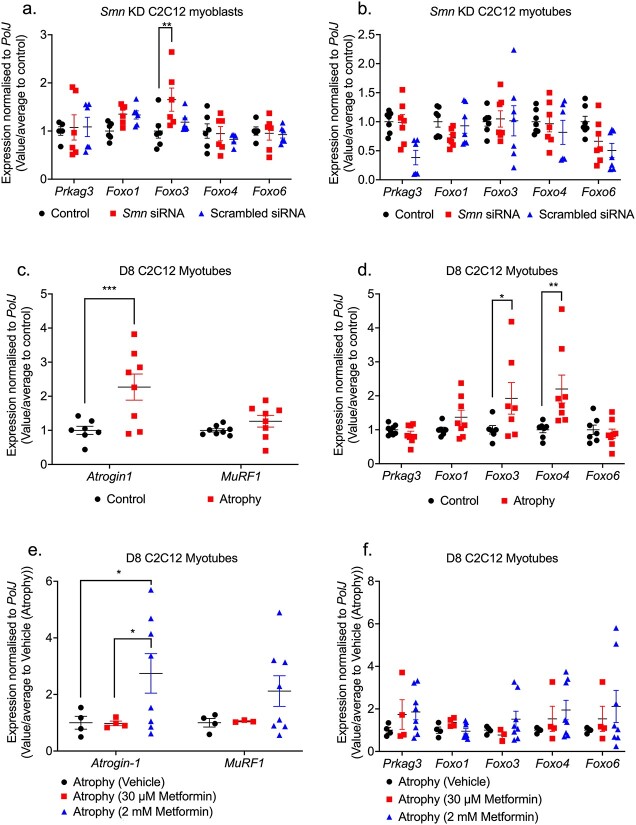
Metformin target genes are pre-dominantly SMN-independent in an SMA muscle C2C12 cellular model. *Smn* siRNA knockdown was performed for (a) 48 h in C2C12 myoblasts and (b) every 48 h throughout differentiation in D8 C2C12 myotubes. mRNA expression of metformin target genes *Prkag3*, *Foxo1*, *Foxo3*, *Foxo4* and *Foxo6* was measured by qPCR and compared to non-transfected and scrambled siRNA transfected controls. D8 C2C12 myotubes were serum-starved for 24 h to induce canonical atrophy. mRNA expression of (c) atrogenes *Atrogin-1* and *MuRF-1* and (d) metformin target genes *Prkag3*, *Foxo1*, *Foxo3*, *Foxo4* and *Foxo6* was measured by qPCR and compared against non-starved myotubes. Serum-starved D8 C2C12 myotubes were treated with either physiological 30 μM metformin or supraphysiological 2 mM metformin for 24 h to evaluate mRNA expression via qPCR of (e) atrogenes *Atrogin-1* and *MuRF-1* and (f) metformin target genes *Prkag3*, *Foxo1*, *Foxo3*, *Foxo4* and *Foxo6* compared to serum-starved PBS vehicle treated control. Data are shown as scatter plot that represent mean ± SEM error bars; n = 4 samples per group across two independent experiments. Two-way ANOVA followed by uncorrected Fisher’s least significant difference (LSD). F = 3.543 (a); F = 2.332 (b); F = 4.9 (c); F = 3.493 (d); F = 0.057 (e); F = 0.235 (f); F = 0.401, ^*^P < 0.05, ^*^^*^P < 0.01, ^*^^*^^*^P < 0.001.

We next wanted to better understand if the aberrant expression of the metformin target genes was dependent on SMN expression and/or muscle atrophy. Thus, we firstly generated small interfering RNA (siRNA)-mediated Smn-depleted C2C12 myoblast-like cells, a useful and previously successful *in vitro* model [65]. We confirmed by qPCR that *Smn* mRNA levels were significantly reduced by up to 90% in C2C12 myoblasts and D8 C2C12 myotubes compared to scrambled siRNA and untreated controls (Fig. S4). We next investigated the effects of Smn knockdown on the expression of the predicted metformin target genes. In C2C12 myoblasts, we identified a significant upregulation of only the *FoxO3* gene in Smn-depleted C2C12 myoblasts compared to controls (Fig. 5a), which reflects previous microarray analyses of specific *FoxO* isoforms upregulated in quadriceps femoralis muscle biopsies from Type 1 SMA patients [66]. However, in C2C12 myotubes we found that *Smn* knockdown (KD) had no effect on the expression of predicted metformin target genes (Fig. 5b), suggesting that for the most part, the expression of the predicted metformin genes is Smn-independent, thus representing ideal targets for SMN-independent therapies.

We next investigated if the expression of the predicted metformin target genes is affected *in vitro* by muscle atrophy. However, one difficulty in mimicking SMA muscle atrophy *in vitro* is establishing denervation. Thus, based on evidence of shared pathway similarities from different pro-atrophy factors such as starvation and denervation [67], we used a validated method of 24-h serum-starvation in C2C12 myotubes to induce canonical atrophy, as confirmed by myotube loss and upregulation of pro-atrophic *atrogin-1* levels (Fig. 5c). Next, we evaluated the expression of the predicted metformin target genes and observed a significant upregulation of *FoxO3* and *FoxO4* isoforms (Fig. 5d), reflecting their established roles in atrophy-dependent ubiquitin-proteasome pathways [43].

We then evaluated whether metformin could attenuate muscle atrophy in C2C12 myotubes. Based on initial gene-dose response experiments in both control C2C12 myoblasts and D8 myotubes, we treated our cells with physiological (60 μM) and supraphysiological (2 mM) metformin concentrations for 24 h (Fig. S5) [68]. The 30 μM physiological metformin concentration for 24 h did not attenuate muscle atrophy or impact the expression of the target genes in the serum starved C2C12 myotubes (Fig. 5e and f). However, for the supraphysiological 2 mM metformin concentration [68], we observed an upregulation of *Atrogin-1* levels (Fig. 5e), suggesting an exacerbation of muscle atrophy. Further analysis of the predicted metformin target genes revealed no significant impact on their expression patterns either (Fig. 5f), suggesting that exacerbation of atrophy in C2C12 myotubes by supraphysiological metformin concentrations involves factors mostly outside of our predicted targets. However, it should be noted that metformin may have different effects in SMA muscle as there are still differences between distinct pro-atrophic factors [67].

Overall, our *in vitro* studies revealed that although most of our predicted metformin target genes are SMN-independent with some linked to muscle atrophy, they were mostly not linked to metformin’s influence on canonical atrophy in C2C12 myotubes.

### Dose-dependent effect of metformin on disease progression and survival in *Smn^2B/−^* SMA mice

Next, we assessed metformin in the *Smn^2B/−^* SMA mouse model [69]. The rationale for conducting our *in vivo* pharmacological studies in the *Smn^2B/−^* SMA mice was based on their longer lifespan [69], responsiveness to SMN-independent therapies [42, 47], established metabolic and myopathy defects [43, 47, 69–72], and later symptomatic onset [69] making them a clinically relevant model for starting treatment regimens >P5 time-points [42, 47].

**Figure 6:**
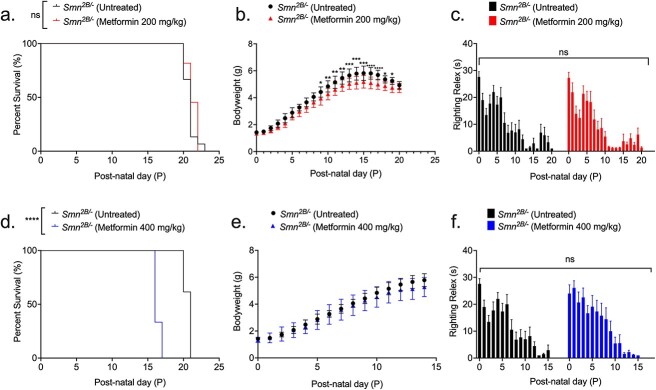
200 mg/kg/day metformin does not improve disease phenotype, while 400 mg/kg/day metformin reduces survival. All treated animals received a daily dose of metformin (either 200 or 400 mg/kg/day, diluted in 0.9% saline) by gavage starting at P5. (a) Survival curves of untreated (n = 13, 21 days median survival) and 200 mg/kg/day metformin-treated (n = 11, 21 days median survival) *Smn^2B/−^* SMA mice. Kaplan-Meier survival curve shown with log rank (Mantel-Cox) test, ns = not significant, *P* = 0.237. (b) Daily weights of untreated (n = 13) and 200 mg/kg/day metformin-treated (n = 11) *Smn^2B/−^* SMA mice. Data represented as mean ± SEM error bars; two-way ANOVA followed by a Sidak’s multiple comparison test, F = 402.1, df = 455, ^*^P < 0.05, ^*^^*^P < 0.01, ^*^^*^^*^P < 0.001, ^*^^*^^*^^*^P < 0.0001. (c) Daily righting reflex test for motor function activity up to a 30 s maximum time point in untreated (n = 13) and 200 mg/kg/day metformin-treated (n = 11) *Smn^2B/−^* SMA mice. Data are shown as bar chart with mean ± SEM error bars; unpaired T-test, ns = not significant, *P* = 0.833. (d) Survival curves of untreated (n = 13, 21 days median survival) and 400 mg/kg/day metformin-treated (n = 4, 16 days median survival) *Smn^2B/−^* SMA mice. Kaplan-Meier survival curve shown with log rank (Mantel-Cox) test, ^*^^*^^*^^*^P < 0.0001. (e) Daily weights of untreated (n = 13) and 400 mg/kg/day metformin-treated (n = 9) *Smn^2B/−^* SMA mice. Data represented as mean ± SEM error bars; two-way ANOVA followed by a Sidak’s multiple comparison test, F = 184.9.1, df = 300. (f) Daily righting reflex test for motor function activity up to a 30 s maximum time point in untreated (n = 13) and 400 mg/kg/day metformin-treated (n = 9) *Smn^2B/−^* SMA mice. Data are shown as bar chart with mean ± SEM error bars; unpaired T-test, ns = not significant, *P* = 0.733.

We initially administered a 200 mg/kg daily dose (diluted in 0.9% saline) starting from P5 until humane endpoint in *Smn^2B/−^* SMA mice and *Smn^2B/+^* healthy control littermates, based on previous success in the muscle disorder CMDT1A, where metformin concentrations ranged from 100–250 mg/kg [60]. We observed no significant improvement in survival of *Smn^2B/−^* SMA mice treated with 200 mg/kg/day compared to untreated (Fig. 6a) and vehicle-treated animals (Fig. S6). We also found a significant reduction in the body weight of 200 mg/kg/day metformin-treated *Smn^2B/−^* SMA mice compared to untreated SMA animals, beginning 4 days after initial treatment at P9 (Fig. 6b). However, we did not observe any effects on weight in the 200 mg/kg/day metformin treated *Smn^2B/+^* healthy control mice (Fig. S6b), indicating a disease specific effect of metformin. In terms of motor function, there was no significant difference in the righting reflex between untreated and 200 mg/kg/day metformin-treated *Smn^2B/−^* SMA mice (Fig. 6c) and *Smn^2B/+^* healthy control animals (Fig. S7a–c).

Since our initial 200 mg/kg/day metformin dosage did not improve disease onset or disease progression in *Smn^2B/−^* SMA mice, we conducted pilot studies with a later treatment start point (P8) and a lower dose (100 mg/kg/day), which demonstrated similar effects to our initial dosing regimen (data not shown). We therefore tried a higher daily dosage of 400 mg/kg/day, starting at P5. Surprisingly, the higher 400 mg/kg/day dose significantly reduced survival in *Smn^2B/−^* SMA pups by 5 days (Fig. 6d), while having no significant impact on weight or righting reflex (Fig. 6e and f). Interestingly, 400 mg/kg/day metformin had no adverse effects in the healthy *Smn^2B/+^* control littermates (Fig. S7d–f), suggesting that the adverse effects of the higher dose of metformin is disease specific.

Thus, our *in vivo* experiments demonstrated that metformin did not emulate prednisolone’s beneficial effects on SMA disease progression in SMA mice and is in fact not an ideal therapy candidate, due to dose- and disease-dependent adverse effects in SMA.

### Higher 400 mg/kg/day metformin dosage is associated with hypoglycaemia in non-fasted *Smn^2B/−^* SMA mice

**Figure 7:**
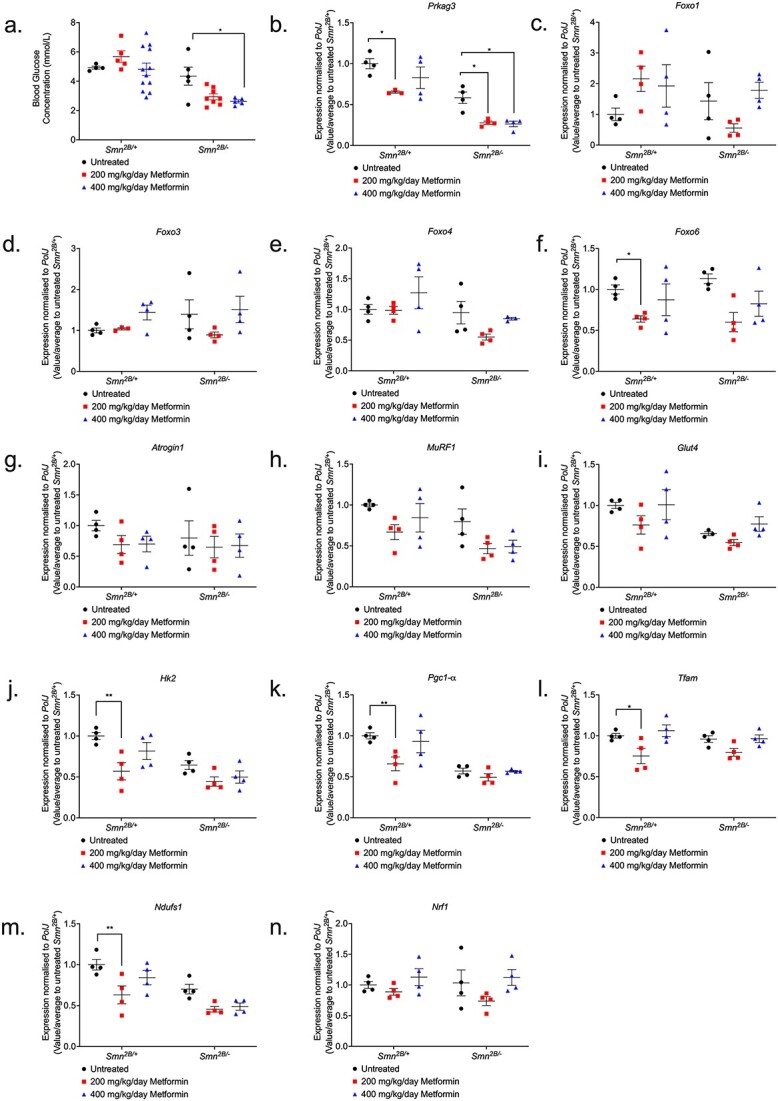
400 mg/kg/day metformin significantly lowers blood-glucose levels in *Smn^2B/−^* SMA mice, with no impact on markers of atrophy, glucose metabolism and mitochondrial regulation in skeletal muscle. (a) Blood-glucose concentrations (mmol/L) were measured 2 h after final treatment from untreated and 200 (red) or 400 mg/kg/day metformin-treated, non-fasted P14 *Smn^2B/+^* healthy and *Smn^2B/−^* SMA mice. Data represented as bar chart with scatter graph represented as mean ± SEM error bars, n = 4 animals per group; two-way ANOVA with Tukey’s multiple comparisons test, F = 25.49, ^*^P < 0.05. qPCR analysis of predicted metformin target genes (b) *Prkag3*, (c) *Foxo1*, (d) *Foxo3*, (e) *Foxo4*, (f) *Foxo6*; atrogenes (g) *Atrogin-1*, (h) *MuRF1*; and glucose uptake and metabolism genes (i) *Glut4*, (j) *Hk2*; and mitochondrial regulatory genes (k) *Pgc1-α,* (l) *Tfam,* (m) *Ndufs1* and (n) *Nrf1* in the TA muscle from untreated and 200 or 400 mg/kg/day metformin-treated, P14 *Smn^2B/+^* healthy and *Smn^2B/−^* SMA mice. Data are shown as scatter graph represented as mean ± SEM error bars, n = 4 animals per group; two way ANOVA with Tukey’s multiple comparisons test, *Prkag3* (F = 59.92), *Foxo1* (F = 1.507), *Foxo3* (F = 0.343), *Foxo4* (F = 6.475), *Foxo6* (F = 0.024), *Atrogin-1* (F = 0.381), *MuRF1* (F = 7.838), *Glut4* (F = 9.9), *Hk2* (F = 17.78), *Pgc1-α* (F = 29.84), *Tfam* (F = 0.423) *Ndufs1* (F = 22.66), and *Nrf1* (F = 0.164).

We next investigated the potential causes behind metformin’s adverse effects in SMA mice. With metformin being a glucose lowering agent for T2DM, we initially assessed blood glucose levels in P14 non-fasted, untreated, 200- and 400-mg/kg/day metformin treated *Smn^2B/−^* SMA and *Smn^2B/+^* healthy mice 2 h after the final treatment. This time point was chosen to account for the reduced median survival in the higher dose SMA cohort. We observed that neither 200- or 400-mg/kg/day metformin treatments lowered blood glucose levels in *Smn^2B/+^* healthy mice (Fig. 7a). However, we reported a significant reduction in blood glucose levels in 400 mg/kg/day metformin-treated *Smn^2B/−^* SMA mice compared to untreated SMA animals (Fig. 7a). Our results suggest that hypoglycaemic shock could have been one of the possible causes behind the premature death in the 400 mg/kg/day SMA cohort, further exacerbating the previously reported hypoglycaemia in SMA models [70] and patients [73, 74].

### Metformin did impact the expression of the predicted target genes, but not muscle pathology markers in *Smn^2B/−^* SMA mice

We next evaluated the effect of metformin on the expression of the predicted target genes in TAs from P14 untreated, 200- and 400-mg/kg/day metformin-treated *Smn^2B/−^* SMA and *Smn^2B/+^* healthy mice 2 h after final treatment. Contrary to the drug repositioning prediction that metformin could reverse *Prkag3* downregulation in SMA muscle (Fig. S3), we instead discovered that both 200- and 400-mg/kg/day metformin doses exacerbated *Prkag3* downregulation in *Smn^2B/−^* SMA muscle (Fig. 7b). Furthermore, although metformin had no impact on *FoxO1*, *FoxO3* and *FoxO4* isoforms in both *Smn^2B/+^* healthy and *Smn^2B/−^* SMA muscle (Fig. 7c–e), for the 200 mg/kg/day *Smn^2B/−^* SMA cohort, metformin significantly reduced *FoxO6* expression (Fig. 7f), which again contrasted our bioinformatics prediction that metformin would upregulate the expression of this isoform in SMA muscle. Thus, the drug-gene response for metformin-treated *Smn^2B/−^* SMA skeletal muscle reveals a contrasting pattern that does not match the bioinformatic predictions.

We next investigated metformin’s effects on the expression of dysregulated molecular markers associated with muscle atrophy (*Atrogin-1* and *MuRF-1*) and glucose metabolism (*Glut4* and *Hk2*) [75, 76]. We however observed that neither atrophy (Fig. 7g and h) or glucose metabolism markers (Fig. 7i and j) were affected by 200- and 400-mg/kg/day metformin treatments in the *Smn^2B/−^* SMA mice when compared to untreated animals.

We also investigated markers associated with mitochondrial biogenesis and function in muscle (*Pgc1-α, Tfam*, *Ndufs1*, and *Nrf1*), as previous research has established that these features are impaired in SMA skeletal muscle [46] and a common mechanism of action for metformin is mild inhibition of mitochondrial electron transport complex 1 (or NADH:ubiquinone oxidoreductase) [77]. We found that that neither the 200- or 400-mg/kg/day dose of metformin impacted the expression of mitochondrial genes in the skeletal muscle of *Smn^2B/−^* SMA mice (Fig. 7k–n), although we do observe a significant downregulation of *Pgc1-α, Tfam*, and *Ndufs1* in the skeletal muscle of 200 mg/kg/day metformin-treated *Smn^2B/+^* healthy counterparts (Fig. 7k–n).

Overall, our data highlights that metformin did not have a direct impact on the predicted target genes in skeletal muscle of SMA mice. Furthermore, the absence of direct impact on muscle atrophy, glucose metabolism, and mitochondrial function markers following metformin treatment in *Smn^2B/−^* SMA muscle, suggests that the adverse effects associated with the 400 mg/kg/day dosage may not have been linked to muscle-intrinsic effects.

### A higher dose of metformin is associated with dysregulation of mitochondrial regulatory genes in the spinal cord of *Smn^2B/−^* SMA mice

We next investigated the effects of metformin on the spinal cord given that metformin is systemically distributed [78], has the ability to cross the blood-brain-barrier (BBB) [79] and can impact the mitochondria in the spinal cord [80]. We thus evaluated whether metformin altered the expression of mitochondrial markers (*Pgc1-α*, *Tfam*, *Nrf1* and *Ndufs1*) in the spinal cord of P14 untreated, 200- and 400-mg/kg/day metformin treated *Smn^2B/−^* SMA mice compared to *Smn^2B/+^* healthy mice, 2 h after final treatment.

**Figure 8:**
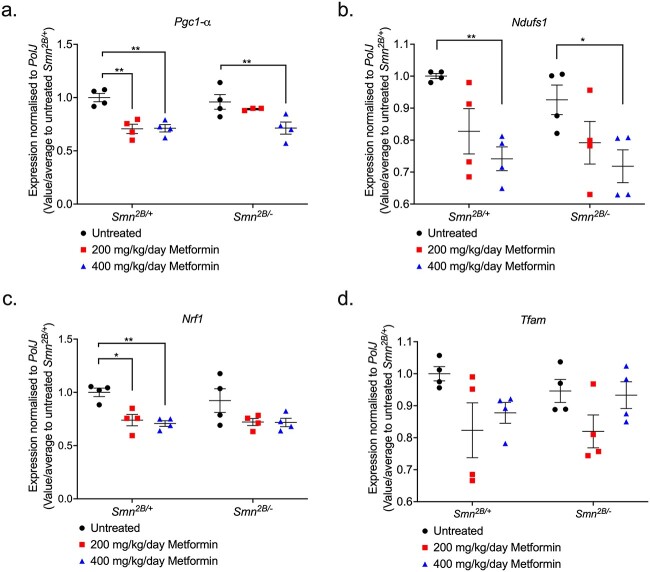
400 mg/kg/day metformin dysregulates mitochondrial regulatory genes exclusively in spinal cord tissue from Smn*^2B/−^* SMA mice. qPCR analysis of mitochondrial regulatory genes (a) *Pgc1-α,* (b) *Ndufs1,* (c) *Nrf1* and (d) *Tfam* in the spinal cord from untreated and 200 or 400 mg/kg/day metformin-treated, P14 *Smn^2B/+^* healthy and *Smn^2B/−^* SMA mice. Data are shown as scatter graph represented as mean ± SEM error bars, n = 4 animals per group; two-way ANOVA with Tukey’s multiple comparisons test. (a) *Pgc1-α* (F = 1.526), (b) *Ndufs1* (F = 1.135), (c) *Nrf1* (F = 0.362) and (d) *Tfam* (F = 0.614), ^*^P < 0.05, ^*^^*^P < 0.01.

For *Pgc1-α*, a master regulator of mitochondrial biogenesis and function, we observed that although both 200- and 400-mg/kg/day metformin concentrations significantly reduced its expression levels in *Smn^2B/+^* healthy spinal cords (Fig. 8a), it was only the higher concentration that significantly reduced *Pgc1-α* expression in *Smn^2B/−^* SMA spinal cords (Fig. 8a). Similarly, 400 mg/kg/day metformin significantly reduced *Ndufs1* levels in both *Smn^2B/+^* healthy and *Smn^2B/−^* SMA spinal cords (Fig. 8b), suggesting that for these mitochondrial health markers, the higher metformin dose negatively affected their expression independent of disease status. On the other hand, although not affected by metformin in *Smn^2B/−^* SMA spinal cords, *Nrf1* gene expression was significantly reduced by both metformin doses in the spinal cord of *Smn^2B/+^* healthy mice (Fig. 8c), while *Tfam* was not affected by metformin in either cohort (Fig. 8d).

Our results demonstrating that the higher dose of metformin (400 mg/kg/day) appears to specifically dysregulate certain mitochondrial genes in the spinal cord of *Smn^2B/−^* SMA mice is supported by recent evidence of tissue-dependent differences in conserved cellular processes between SMA motor neurons and skeletal muscle [49]. Thus, although further in-depth investigations would be needed, our results on mitochondrial health markers suggest that metformin’s adverse effects in SMA mice could be linked to the exacerbation of neuronal mitochondrial dysfunction.

### Oxandrolone’s predicted target gene, *Ddit4,* is dysregulated in the skeletal muscle of severe *Smn^−/−^;SMN2* SMA mice

Our second drug candidate that we selected to mimic prednisolone activities was oxandrolone, a synthetic orally bioavailable anabolic steroid that confers minimal androgynous effects [56]. Importantly for SMA, oxandrolone has been successful in the promotion of muscle growth for DMD [81] and mixed gender burn injury patients [82].

Oxandrolone was predicted to upregulate the *Ar* gene in SMA muscle (Fig. S8). The upregulation of *Ar* was predicted to directly upregulate downstream effectors *Igfbp5* and *myogenin* (or *MyoG*) (Fig. S8), which both regulate muscle differentiation, regeneration and myofiber growth [83, 84]. Furthermore, *Ar* was predicted to indirectly upregulate *Dok5*, a signalling protein linked to insulin and IGF-1 activity [85] and *Akap6*, which is involved in the modulation of muscle differentiation and regeneration [86] (Fig. S8). In addition to these factors, we also decided to investigate *Ddit4* as an oxandrolone target based on its direct relation with *Ar* [87] and being one of the top 20 downregulated DEG targets of prednisolone in *Smn^−/−^;SMN2* SMA skeletal muscle (Table S2; Fig. S8).

**Figure 9:**
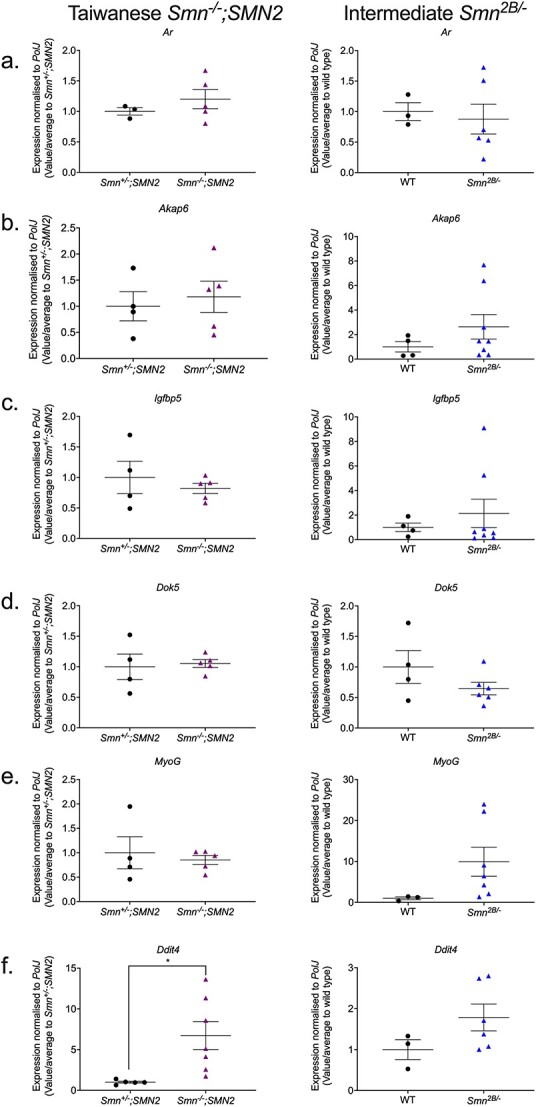
The oxandrolone target gene, *Ddit4*, is significantly upregulated in the skeletal muscle of severe *Smn^−/−^;SMN2* SMA mice. qPCR analysis of mRNA levels for predicted oxandrolone target genes (a) *Ar*, (b) *Akap6*, (c) *Igfbp5*, (d) *Dok5,* (e) *MyoG* and (f) *Ddit4* in the harvested triceps of untreated P7 Taiwanese *Smn^−/−^;SMN2* SMA mice and healthy *Smn^+/−^;SMN2* controls (left panel) and symptomatic untreated P19 milder *Smn^2B/−^* SMA mice and wild type (C57BL/6J background) controls (right panel). Data are shown as scatter plot that represents mean ± SEM error bars; n = 4–7 animals per experimental group, unpaired t-test, ^*^P < 0.05. *Smn^−/−^;SMN2 Ar*: *P* = 0.38; *Smn^−/−^;SMN2 Akap6*: *P* = 0.68; *Smn^−/−^;SMN2 Igfbp5*: *P* = 0.49; *Smn^−/−^;SMN2 Dok5*: *P* = 0.79; *Smn^−/−^;SMN2 MyoG*: *P* = 0.64; *Smn^−/−^;SMN2 Ddit4*: *P* = 0.02; *Smn^2B/−^ Ar*: *P* = 0. 75; *Smn^2B/−^ Akap6*: *P* = 0. 29; *Smn^2B/−^ Igfbp5*: *P* = 0.52; *Smn^2B/−^ Dok5*: *P* = 0.19; *Smn^2B/−^ MyoG*: *P* = 0.15, *Smn^2B/−^ Ddit4*: *P* = 0.16.

Similar to our metformin strategy above, we initially wanted to evaluate the mRNA expression levels of these target genes in the triceps of both symptomatic P7 severe *Smn^−/−^;SMN2* and P19 milder *Smn^2B/−^* SMA mice alongside their respective healthy controls. Overall, we identified no significant dysregulated expression of the target genes *Ar*, *Akap6*, *Igfbp5*, *Dok5* and *MyoG* in both severe *Smn^−/−^;SMN2* and milder *Smn^2B/−^* SMA mice (Fig. 9a–e). However, for *Ddit4,* we did identify a significant upregulation only in *Smn^−/−^;SMN2* SMA mice (Fig. 9f), supporting both our bioinformatics data for this gene and its known pro-atrophic role [87], indicating that it may play an important role in SMA muscle pathologies. In summary, the majority of the predicted oxandrolone target genes did not significantly reflect their bioinformatic predictions.

### *In vitro* oxandrolone treatment prevents canonical atrophy in C2C12 myotubes independently of the predicted Smn-independent targets

**Figure 10:**
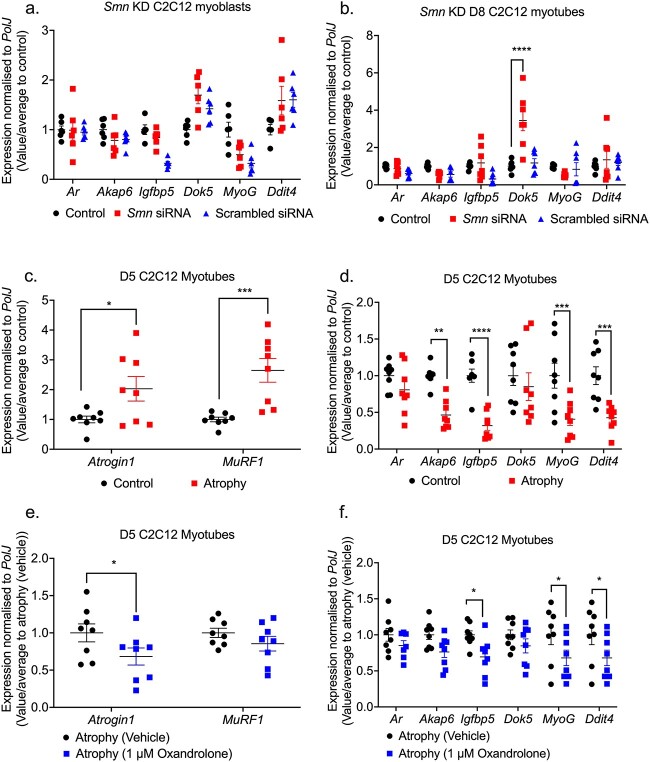
Oxandrolone target genes are pre-dominantly SMN-independent in SMA muscle C2C12 cellular model. *Smn* siRNA knockdown (red) was performed for (a) 48 h in C2C12 myoblasts and (b) every 48 h throughout differentiation in D8 C2C12 myotubes. mRNA expression of oxandrolone target genes *Ar*, *Akap6*, *Igfbp5*, *Dok5, MyoG* and *Ddit4* was measured by qPCR and compared to non-transfected and scrambled siRNA transfected controls. D5 C2C12 myotubes were serum-starved for 24 h to induce canonical atrophy. mRNA expression of (c) atrogenes *Atrogin-1* and *MuRF-1* and (d) oxandrolone target genes *Ar*, *Akap6*, *Igfbp5*, *Dok5, MyoG* and *Ddit4* was measured by qPCR and compared to non-starved myotubes. Serum-starved D5 C2C12 myotubes were treated with1 μM oxandrolone for 24 h to evaluate mRNA expression via qPCR of (e) atrogenes *Atrogin-1* and *MuRF-1* and (f) oxandrolone target genes *Ar*, *Akap6*, *Igfbp5*, *Dok5, MyoG* and *Ddit4* compared to serum-starved absolute ethanol vehicle treated control. Data are shown as scatter graphs that represent mean ± SEM error bars; n = 4 samples per group across two independent experiments. Two-way ANOVA followed by uncorrected Fisher’s least significant difference (LSD). (a) F = 5.45; (b) F = 6.87; (c) F = 1.1; (d) F = 2.03; (e) F = 0.72; (f) F = 0.36, ^*^P < 0.05, ^*^^*^P < 0.01, ^*^^*^^*^P < 0.001, ^*^^*^^*^^*^P < 0.0001.

Similar to our metformin *in vitro* studies, we wanted to evaluate whether reduced SMN levels or atrophy influenced the expression of the predicted oxandrolone target genes in SMA skeletal muscle. Although none of the target genes were affected in the Smn-depleted C2C12 myoblasts (Fig. 10a), we found that *Smn* KD triggered a significant upregulation of *Dok5* only in C2C12 myotubes (Fig. 10b), suggesting that the expression of this gene may be Smn-dependent. Nevertheless, the expression of the majority of the predicted oxandrolone target genes was Smn-independent.

We next wanted to evaluate the ability of oxandrolone to attenuate canonical atrophy in serum-deprived C2C12 myotubes [88]. However, in this case we performed the treatments in D5 C2C12 myotubes instead of D8, as although oxandrolone was non-toxic (Fig. S9), it elicited a greater androgen *Ar* response at the earlier differentiation stage (Fig. S10). Following confirmation of muscle atrophy in D5 C2C12 myotubes via significant *Atrogin-1* and *MuRF1* upregulation (Fig. 10c), we observed that the expression of the predicted oxandrolone target genes *Akap6*, *Igfbp5*, *MyoG* and *Ddit4* was significantly downregulated in serum-deprived D5 C2C12 myotubes (Fig. 10d).

Interestingly, we found that 24-h treatment with 1 μM oxandrolone attenuated canonical muscle atrophy in these serum-starved D5 C2C12 myotubes as shown by significant downregulation of *Atrogin-1* (Fig. 10e). However, we observed that *Igfbp5*, *MyoG* and *Ddit4* were further downregulated by the 1 μM oxandrolone treatment (Fig. 10f), suggesting that oxandrolone’s effects on atrophy are linked to effectors independent of the predicted target genes.

Overall, our *in vitro* studies have shown that although the expression of the predicted oxandrolone target genes is Smn-independent, they are not involved in oxandrolone’s ameliorative effects on canonical atrophy in C2C12 myotubes.

### Oxandrolone treatment improves survival in *Smn^2B/−^* SMA mice

**Figure 11:**
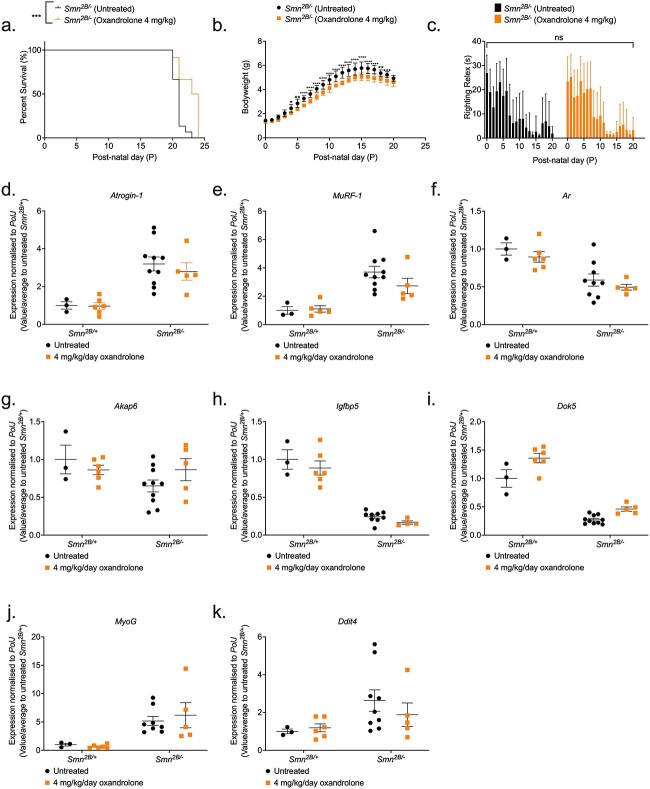
4 mg/kg/day oxandrolone treatment partially improves survival in *Smn^2B/−^* SMA mice. All treated animals received a daily dose of oxandrolone (4 mg/kg/day, suspended in 0.5% CMC) by gavage starting at P8. (a) Survival curves of untreated (n = 15, 21 days median survival) and 4 mg/kg/day oxandrolone-treated (n = 12, 24 days median survival) *Smn^2B/−^* SMA mice. Kaplan-Meier survival curve shown with log rank (Mantel-Cox) test, ^*^^*^^*^P = 0.0006. (b) Daily weights of untreated (n = 15) and 4 mg/kg/day oxandrolone-treated (n = 12) *Smn^2B/−^* SMA mice. Data represented as mean ± SEM error bars; two-way ANOVA followed by a Sidak’s multiple comparison test, F = 610.8, df = 519, ^*^P < 0.05, ^*^^*^P < 0.01, ^*^^*^^*^P < 0.001, ^*^^*^^*^^*^P < 0.0001. (c) Daily righting reflex test for motor function activity up to a 30 s maximum time point in untreated (n = 15) and 4 mg/kg/day oxandrolone-treated (n = 12) *Smn^2B/−^* SMA mice. Data are shown as bar chart with mean ± SEM error bars; unpaired t-test, ns = not significant, *P* = 0.775. qPCR analysis of mRNA levels for atrogenes (d) *Atrogin-1* and (e) *MuRF1* and predicted target genes (f) *Ar,* (g) *Akap6,* (h) *Igfbp5,* (i) *Dok5,* (j) *MyoG,* and (k) *Ddit4* in the triceps muscle from untreated and 4 mg/kg/day oxandrolone-treated, P19 *Smn^2B/+^* healthy and *Smn^2B/−^* SMA mice. Data are shown as bar chart with scatter graph represented as mean ± SEM error bars, n = 4 animals per group; two way ANOVA with Tukey’s multiple comparisons test, *Atrogin-1* (F = 0.1914), *MuRF1* (F = 1.214), *Ar* (F = 0.003), *Akap6* (F = 2.40), *Igfbp5* (F = 0.06), *Dok5* (F = 1.72), *MyoG* (F = 0.29) and *Ddit4* (F = 0.64), ^*^P < 0.05, ^*^^*^P < 0.01.

We next assessed the impact of oxandrolone in SMA mice. We initially tested preliminary treatment regimens of 1–8 mg/kg/day starting from P5 or P8 in *Smn^2B/−^* SMA and *Smn^2B/+^* healthy mice (data not shown), based on previous studies in models of spinal cord injury (SCI) [89] and burn injury [90]. We also stopped oxandrolone treatments at P21 as previous research has shown that shorter oxandrolone treatments are more effective [89]. These pilot studies allowed us to identify the optimal dosing regimen of 4 mg/kg/day oxandrolone treatment from P8 to P21, which significantly improved the median survival of *Smn^2B/−^* SMA mice (Fig. 11a).

However, we found that the body weight of 4 mg/kg/day oxandrolone-treated *Smn^2B/−^* SMA mice was significantly lower compared to their untreated counterparts (Fig. 11b), which is most likely due to the intrinsic smaller sizes of the randomly assigned litters, as demonstrated by the difference in weight starting 4 days prior to initial treatment (Fig. 11b). In terms of motor function, we observed no significant difference in righting reflex between untreated and oxandrolone-treated SMA animals (Fig. 11c). Furthermore, we identified no impact of vehicle treatment on survival, weight, and righting reflex in *Smn^2B/−^* SMA mice (Fig. S11).

In the *Smn^2B/+^* healthy mice, although 4 mg/kg/day oxandrolone had no effect on survival or motor function in treated animals (Fig. S12a and b), we did observe a significant decrease in bodyweight starting from P9, one day after initial treatment (Fig. S12c), suggesting that oxandrolone may have impacted growth.

Nevertheless, our results demonstrated that although 4 mg/kg/day oxandrolone treatment improved survival in *Smn^2B/−^* SMA mice, its effect on survival was still minor compared to prednisolone [27], suggesting that oxandrolone is not a suitable substitute as an SMA skeletal muscle therapy.

### Oxandrolone did not impact the expression of the predicted target genes or muscle pathology markers

The improved 3-day survival in 4 mg/kg/day oxandrolone-treated *Smn^2B/−^* SMA mice led us to evaluate whether this beneficial impact was related to targeting muscle pathologies. Thus, we evaluated oxandrolone’s effects on the expression of dysregulated molecular markers associated with the SMA hallmark pathology of muscle atrophy (*Atrogin-1* and *MuRF-1*) in the triceps from P19 late symptomatic, untreated and 4 mg/kg/day oxandrolone-treated *Smn^2B/−^* SMA and *Smn^2B/+^* healthy mice, 2 h after final treatment. We observed no significant reduction in elevated *Atrogin-1* or *MuRF-1* gene expression levels by oxandrolone in the *Smn^2B/−^* SMA cohort (Fig. 11d and e), suggesting that oxandrolone did not attenuate muscle atrophy.

We next evaluated the effect of oxandrolone on the expression of the predicted target genes in the same P19 untreated and 4 mg/kg/day oxandrolone-treated *Smn^2B/−^* SMA and *Smn^2B/+^* healthy mice. We found that oxandrolone did not significantly impact the predicated target genes in the triceps from the *Smn^2B/−^* SMA mice (Fig. 11f–k). However, we did observe that 4 mg/kg/day oxandrolone treatment significantly upregulated *Dok5* expression in the *Smn^2B/+^* healthy mice (Fig. 11i). Nevertheless, the pattern observed suggests that oxandrolone did not impact any of the predicted genes in the muscle from *Smn^2B/−^* SMA mice.

Overall, our data shows that oxandrolone did not have an efficient effect on the predicted target genes. Furthermore, its inability to ameliorate muscle atrophy marker dysregulation in SMA skeletal muscle, suggests that improved survival in the *Smn^2B/−^* SMA mice by oxandrolone may be independent of targeting skeletal muscle pathologies.

### Both metformin and oxandrolone drug candidates attenuate neuromuscular phenotypes in the *C. Elegans* severe SMA model

**Figure 12:**
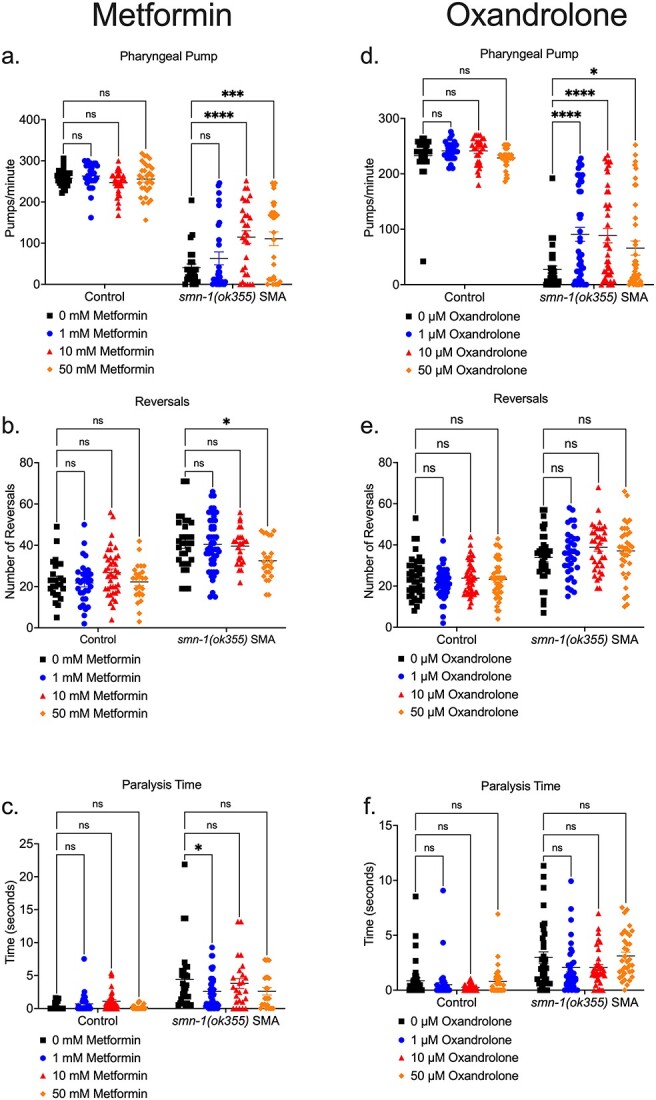
Metformin and oxandrolone partially ameliorates neuromuscular defects in the severe SMA *C. Elegans* model. Day 3 *C. Elegans smn-1(ok355)* SMA homozygotes and *smn-1(ok355)I/hT2* control heterozygotes were maintained at 20°C on nematode growth medium (NGM) plates seeded with *Escherichia coli* OP50 bacteria. In metformin conditions, the NGM contained metformin concentrations of 0, 1, 10 and 50 mM respectively. (a) Pharyngeal pumping rates (pumps/minute) defined as grinder movement in any axis at 175 frames/10 s. Locomotion assays were filmed at 15 frames/sec and quantified for 5 min for (b) reversals and (c) paralysis times in the *C. Elegans* groups. In oxandrolone conditions, the NGM contained oxandrolone concentrations of 0, 1, 10 and 50 μM respectively. (d) Pharyngeal pumping rates (pumps/minute) defined as grinder movement in any axis at 175 frames/10 s. Locomotion assays were filmed at 15 frames/sec and were quantified for 5 min for (e) reversals and (f) paralysis times in the *C. Elegans* groups. Data are shown as scatter plot that represents mean ± SEM error bars; n > 25 animals per experimental group across three independent trials; two-way ANOVA with Tukey’s multiple comparisons test, (a) F = 459.2, (b) F = 108.6, (c) F = 52.59, (d) F = 501.8, (e) F = 121.1, (f). F = 57.06, ns = not significant ^*^P < 0.05, ^*^^*^p < 0.01, ^*^^*^^*^P < 0.001.

We next wanted to investigate our drug candidates in a separate SMA model to assess whether they could attenuate neuromuscular dysfunctions in a distinct species. For this purpose, we used the *C. elegans smn-1(ok355)* invertebrate model [91], based on shared conservation of the SMN protein with vertebrate species [92] and the well described phenotypic defects of larval lethality (reduced survival) and impaired neuromuscular function in pharyngeal pumping for feeding and locomotion [91, 93]. For metformin, administration at higher doses of 50 mM partially ameliorated multiple phenotypes only in *C. elegans smn-1(ok355)*, including pharyngeal pumping defects and the locomotory defect of number of reversals (Fig. 12a and b), however, only the lower dose of 1 mM metformin significantly ameliorated paralysis times (Fig. 12c). On the other hand, oxandrolone across 1–50 μM doses significantly ameliorated pharyngeal pumping defects only (Fig. 12d), with no significant effect on locomotory defects of reversal and paralysis times (Fig. 12e and f), suggesting an improvement in muscular activity in the pharynx region. Overall, given SMN conservation across species, oxandrolone could improve neuromuscular defects across vertebrate and invertebrate models of SMA.

## Discussion

The goal of this study was to use a transcriptomics-based drug repositioning strategy to identify clinically approved drug candidates that could emulate prednisolone’s beneficial effects in SMA skeletal muscle and life expectancy [27], without the risks associated with long-term GC exposure [39].

Our major finding was the observation that prednisolone treatment restored specific gene sets associated with key pathological SMA pathways such as FoxO signalling [43], p53 signalling [44], AMPK signalling [45], mitophagy [46], circadian rhythm [47], PPAR signalling [48], and autophagy [49] in *Smn^−/−^;SMN2* SMA skeletal muscle. Although these pathway results highlight prednisolone’s efficacy in improving skeletal muscle health, it should be noted that our transcriptomic data cannot distinguish whether these restorations are targeted directly by prednisolone or a consequence of improved muscle health. Nevertheless, our transcriptomic data complemented the prior phenotypic data of prednisolone’s potential as a second-generation SMA therapy [27].

Despite a multitude of promising compounds identified that could be investigated in future studies, one of the findings of our study was that neither of our chosen orally bioavailable drug candidates, metformin and oxandrolone, reproduced prednisolone’s previously reported effect on muscle growth and survival in SMA mice [27]. For metformin, we observed that both 200- and 400-mg/kg/day doses counterintuitively exacerbated *Prkag3* downregulation in *Smn^2B/−^* SMA muscle instead of the predicted upregulation, which could have negative consequences since *Prkag3^−/−^* null mice presented metabolic and mitochondrial dysregulation [94, 95] similar to those reported in SMA patients [46, 74] and models [70, 71]. More surprising was the reduced survival of *Smn^2B/−^* SMA mice treated with 400 mg/kg/day metformin, which we potentially linked to possible hypoglycaemic shock and/or dysregulation of neuronal mitochondrial components. On the other hand, although various metformin doses ameliorated neuromuscular dysfunction in our SMA *C. elegans* model, the negative effects observed in *Smn^2B/−^* SMA mice could be to due vertebrate vs invertebrate systems. Curiously, previous research into AMPK agonism via 500 mg/kg AICAR treatment in the *SmnΔ7* SMA mice contrasts our metformin data, as they showed improved skeletal muscle health and no exacerbations in neuronal dysfunction [45]. One explanation for metformin and AICAR’s conflicting results in SMA mice could be related to differential pharmacological activities. Indeed, AICAR is an AMP analog with a low BBB penetrability [96] that directly binds to the γ-AMPK isoform [97, 98], while metformin can rapidly penetrate the BBB [79] and has been associated with direct and indirect AMPK activation [78], an example of the latter involving mitochondrial respiratory complex 1 inhibition [77]. With emerging evidence of naturally low mitochondrial respiratory complex 1 activity in SMA motor neurons [99] and SMA pathway profiles being tissue-dependent [49], one theory could be that the 400 mg/kg/day metformin dose exacerbated mitochondrial respiratory complex 1 inhibition in SMA motor neurons. However, future studies would be needed to verify this proposed model. Nonetheless, our findings could be important for clinical drug safety, as with reported co-morbidities of diabetes in certain SMA patients [100, 101], our pre-clinical data suggests lower metformin doses or non-biguanide drugs may be important to manage diabetes and not risk primary pathologies in SMA patients.

For oxandrolone, our mouse data showed that 4 mg/kg/day treatment from P8 partially improved survival in *Smn^2B/−^* SMA mice, although not to the same extent observed with prednisolone [27]. In addition, we identified that the lower dose of 1 μM oxandrolone *in vitro* attenuated canonical atrophy in C2C12 myotubes, while *in vivo* oxandrolone attenuated neuromuscular dysfunction in severe SMA *C. elegans* model, suggesting that in both our SMA vertebrate and invertebrate species oxandrolone may be a beneficial SMN-independent treatment option.

An additional factor that could contribute to the different pharmacological response in observed in our mouse and *C. elegans* models is sex-specific differences. Indeed, emerging clinical evidence collected from 3838 SMA patients across 18 countries shows that severe neuromuscular pathologies are more frequent in SMA types 2 and 3b male patients compared to females [102]. Furthermore, both metformin and oxandrolone have demonstrated sex-specific effects on muscle pathology. Indeed, metformin treatment significantly improved skeletal muscle function only in the female mouse model for CMDT1A [60]. On the other hand, studies with *Ar* knock out (KO) rodent models have demonstrated that Ar absence does not have the same impact on female muscle size compared to males [103]. In addition, it is suggested that intramuscular Ar content may have a stronger influence on hypertrophy than peripheral androgen levels [104], suggesting that oxandrolone may be more beneficial in males. Thus, future studies need to take into consideration the sex of the SMA models when evaluating drug efficacy.

Despite our study’s limitations, it highlighted refinements for future *in silico* SMA drug repositioning studies. Compared to a previous study that successfully identified and validated harmine’s therapeutic potential in SMA muscle [42], ours did not include proteomics. The absence of proteomics can be a caveat for drug studies as transcript levels alone do not proportionally reflect drug-protein interactions, abundance and translational modifications [105, 106]. However, a limitation of both transcriptomics and proteomics approaches is that they cannot bridge drug-pathway interactions with disease phenotypes, as demonstrated by a recent proteomics analysis of Spinraza®-treated Type 2 and 3 SMA patients that could not correlate protein profiles with functional improvements [107]. Thus, implementation of metabolomics may be beneficial for linking pathway perturbations with metabolites associated with disease and stages of muscle atrophy [108]. To the best of our knowledge, this three-pronged multi-omics approach has not previously been used in SMA drug repositioning, however it has been successful in the identification of 200 biomarker candidates for SMA [109]. Importantly, and as demonstrated in our previous work [42], the power of multi-omics lies in the ability of further defining the molecular and signalling changes that occur in cells and tissues following treatment, which can allow for the refinement of therapeutics targets and repurposed drugs as well as for the investigation of potential mechanisms behind responsive and non-responsive cohorts.

Another consideration is systemic effects of the drugs as seen with the enhanced lethality of the 400 mg/kg/day metformin’s dose in *Smn^2B/−^* SMA mice being linked to hypoglycaemia and mitochondrial dysfunction in neuronal tissue. Indeed, adverse systemic risks were also found with a previous multi-omics drug repositioning study for SMA muscle that identified the development of tremors in harmine-treated *Smn^2B/−^* SMA mice [42]. With tissue-dependent differences in conserved pathways in SMA [49], future omics studies could integrate data from both neuronal and skeletal muscle to minimize systemic adverse risks.

Even with these refinements, future drug repositioning studies for SMA skeletal muscle pathology may need to consider replacing bulk RNA-Seq. Skeletal muscle fibres are comprised of a myriad of different muscle fibre and cell types, alongside non-muscular interconnecting tissues such as neurons, tendons, adipose, immune cells and capillaries [110, 111], which altogether does not truly represent the transcriptomic profiles for distinct skeletal muscle cells. Indeed, our significant KEGG pathway results included those associated exclusively with neuronal, immune, and capillary cells such as glioma, atherosclerosis and Th17 cell differentiation. With alterations in fibre type composition [72, 112] and satellite cell dysregulation evidenced in SMA muscle [23], an emerging alternative to predict drug candidates that target dysregulated SMA pathways in these muscle types would be single-cell (scRNA-Seq) and/or single nuclear RNA-Seq (snRNA-Seq) [111], which have already been useful tools in other muscular disorders such as DMD [113, 114]. In addition, snRNA-Seq could have further benefits to narrow in on muscle regions such as nuclei located near the NMJ, since myonuclei display spatial and temporal expression pattern heterogeneity in multi-nucleated myofibers [111].

Although the drug candidates metformin and oxandrolone did not emulate prednisolone’s beneficial effects in SMA to the extent previously reported, our transcriptomics-drug repositioning approach did better define prednisolone’s activity in SMA muscle and provided a list of potential candidates for future pre-clinical SMA drug repositioning studies. Furthermore, our study highlights important refinements for future SMA drug repositioning studies.

## Materials and Methods

### Animal procedures

Experiments with the severe (Taiwanese) *Smn^−/−^;SMN2 (*FVB/N background, FVB.Cg-*Smn1tm1HungTg(SMN2)2Hung/J*) SMA mice [40] and *Smn^+/−^;SMN2* healthy control littermates were carried out in the University of Oxford Biomedical Sciences Unit (BSU), in accordance with the UK Home Office authorisation (Animals Scientific Procedures Act (1986), UK Home Office Project Licence PDFEDC6F0).

The *Smn^2B/−^* SMA mouse model [69] was generated by breeding *Smn^2B/2B^* mice (generously provided by Dr Rashmi Kothary (University of Ottawa), Dr Lyndsay Murray (University of Edinburgh) and Professor Matthew Wood (University of Oxford) before being sent to Charles River for rederivation) with *Smn^+/−^* mice (B6.Cg-*Smn1/J*, stock #007963, Jackson Labs). All live procedures on wild type (WT) (C57BL/6 background), *Smn^2B/−^* SMA and *Smn^2B/+^* healthy littermates were performed in the Keele University BSU, in accordance with the UK Home Office authorisation (Animals Scientific Procedures Act (1986), UK Home Office Project Licence P99AB3B95).

For all behavioural experiments, body weights and righting reflex [115] (up to 30 s) were assessed daily from birth until animals reached their humane endpoint, defined in our UK Home Office Project Licence (P99AB3B95) as the time at which the animal displays hindlimb paralysis, immobility, inability to right (greater than 30 s), 4 consecutive days of weight loss and/or up to 20% body weight loss.

As previously described [27], prednisolone (5 mg tablet, Almus) (5 mg/kg dissolved in water) was administered every second day by gavage from post-natal day (P)0 to P7 in *Smn^−/−^;SMN2* SMA and *Smn^+/−^;SMN2* healthy mice and from P0 to P20 in *Smn^2B/−^* SMA and *Smn^2B/+^* healthy mice. Metformin hydrochloride (#PHR1084, Sigma-Aldrich) was dissolved in 0.9% saline physiological solution (tablet dissolved in sterile water) (#07982, Sigma) and administered daily (200 or 400 mg/kg) by gavage from P5 to humane endpoint in *Smn^2B/−^* SMA, *Smn^2B/+^* healthy and WT mice. Oxandrolone (#SML0437, Sigma-Aldrich) was prepared in 0.5% carboxymethylcellulose (CMC) solution (powder dissolved in 0.9% saline solution) (#C5678, SLS) by sonication at 37 kHz for 3 min and administered (4 mg/kg) daily by gavage from P8 to P21 in *Smn^2B/−^* SMA, *Smn^2B/+^* healthy and WT mice.

### Blood-glucose measurement

Blood was collected from non-fasted *Smn^2B/−^* SMA and *Smn^2B/+^* healthy mice and glucose levels were immediately measured (mmol/l) via True Metrix® GO Self-Monitoring Blood Glucose Meter (Trividia Health™) and True Metrix® Test Strips (Trividia Health™).

### RNA-sequencing (RNA-Seq)

Total RNA was extracted from triceps of symptomatic P7 untreated and prednisolone-treated Taiwanese *Smn^−/−^;SMN2* SMA and *Smn^+/−^;SMN2* healthy mice (Table S12). The triceps were immediately placed in RNALater (#AM7030, ThermoFisher) following dissection and stored at −20°C under further analysis. For mRNA isolation, 500 ng of total RNA was used as input for the NEBNext® Poly(A) mRNA Magnetic Isolation Module’ (#E7490L, New England Biolabs (NEB)) in accordance with the manufacturer’s standard instruction. Library preparation was carried out using the NEBNext® Ultra Directional RNA Library Prep Kit for Illumina (#E7420L, NEB). Barcoded libraries from each experimental sample were combined in equimolar concentrations of 1.5 pM prior to sequencing at 75 bp × 1 (single-end) read metric on a NextSeq 550 (Illumina) system.

### Differential gene expression analysis

For the RNA-Seq data from the triceps of P7 untreated and prednisolone-treated Taiwanese *Smn^−/−^;SMN2* SMA and *Smn^+/−^; SMN2* healthy mice (Table S12), differential gene expression (DGE) analysis was performed in Galaxy (usegalaxy.org) [116]. After initial quality control assessments via FastQC v0.72 + galaxy1 [117], we trimmed reads based on SLIDINGWINDOW of 4 bp at average quality read of 32 in Trimmomatic v0.36.5 [118] and trimmed the first 12 abnormal bases in Trim sequences v1.0.2 [119]. After quality control confirmation with FastQC v0.72 + galaxy1 [117] the processed 63 bp single-reads were aligned to an in-built UCSC *Mus musculus* mm10 genome via HISAT2 v2.1.0 [116, 120] under a reverse (or antisense) strand setting. Count quantification of aligned single-reads to mapped coding genes was performed by FeatureCounts v1.6.3 + galaxy2 [116, 121] using an in-built Entrez *Mus musculus* mm10 gene transfer file (GTF) with known gene identifier set at *-exon level*. Mapping and count quantification was visualized through MultiQC v1.6 [122]. For DGE analysis of our raw transcript counts, we used DESeq2 v2.11.40.2 [123] under the design formula of “Condition” (SMA vs Healthy) and “Treatment” (Untreated vs Prednisolone) after removal of 1 outlier (prednisolone-treated *Smn^−/−^;SMN2* sample N0603). We set differentially expressed gene (DEG) significance at log2 fold change (FC) > 0.6 and false discovery rate (FDR) < 0.05. The Entrez Gene IDs were translated to official *Mus musculus* gene symbols by AnnotatemyID v3.7.0 [116]. The normalized count files generated by DESeq2 of prednisolone-treated Taiwanese *Smn^−/−^;SMN2* SMA mice vs untreated *Smn^−/−^;SMN2* SMA and *Smn^+/−^;SMN2* healthy mice were generated into heatmaps by Heatmap2 v2.2.1 + galaxy1 [116] under -*data log2 transformed* and scaled by *-row scale genes*.

### Pathway analysis and drug repositioning

Pathway analysis of the prednisolone-treated vs untreated Taiwanese *Smn^−/−^;SMN2* SMA mice was performed with iPathwayGuide [50] (Advaita) with default criteria of log2FC > 0.6 and FDR < 0.05 for DEGs. The impact analysis performed by iPathwayGuide incorporated our DEGs expression (log2FC) and its topological position in the KEGG pathway database [124] to calculate significantly impacted pathways *P* < 0.05 (KEGG v1910 Release 90.0+/05–29, May 19, GODb v1910 2019-Apr 26). Furthermore, overrepresentation analysis [125] (ORA) with elimination pruning [126] was performed for gene ontology (GO) pathways [127] and predicted upstream regulators (STRING v11.0, Jan 19^th^, 2019). Drug candidate identification was performed through an in-built KEGG drugs database [124] aligned to KEGG pathways in iPathwayGuide and by drug-gene interactions for upstream regulators in the Drug Gene Interaction Database [51] (DGIdb) v3.0.

### Molecular docking

Ligands were modelled and optimized using LigPrep (2021-4, Schrödinger LLC, New York, NY) and MacroModel (2021-4, Schrödinger LLC, New York, NY) according to the OPLS4 (2021-4, Schrödinger LLC, New York, NY) forcefield parameters [128]. Protein structures were downloaded from the RCSB databank [129] and prepared using the Protein Preparation Wizard (2021-4, Schrödinger LLC, New York, NY) [130], where redundant molecules are removed, hydrogen and missing heavy atoms were added, bond orders were assigned, water orientations were sampled, ionization and tautomeric states of certain residues are set. Restrained force field minimization was applied by converging heavy atoms to 3.0 Å and receptor grids were generated for the active sites. Molecular docking was performed using Glide (2021-4, Schrödinger LLC, New York, NY) at extra precision mode at 100 runs per each ligand-receptor complex [131].

Methodology was validated by a redocking of the co-crystallized ligand for each protein structure.

### C2C12 cell culture

The immortalized murine C2C12 myoblast-like cell line [132] (#CRL-1772, ATCC, USA) was maintained in growth media comprised of high glucose (4.5 g/l) and L-glutamine (0.6 g/l) Dulbecco’s Modified Eagle’s Media (DMEM) (#BE12-741F, Lonza), 10% foetal bovine serum (FBS) (#10438026, Gibco), and 1% penicillin-streptomycin (10 000 U/ml) (#15140122, Gibco). C2C12 myoblasts were differentiated into myotubes in a differentiation media comprised of high glucose (4.5 g/l) and L-glutamine (0.6 g/l) DMEM (#BE12-741F, Lonza), 2% horse serum (HS) (#26050070, Gibco), 1% penicillin-streptomycin (10 000 U/ml) (#15140122, Gibco), and 0.1% insulin (1 μg/ml) (#I6634, Sigma) for 2–8 days with media replacement every 48 h. All cultured cells were incubated in humid 37°C and 5% CO_2_ conditions (Heracell 150i CO_2_ incubator, ThermoScientific).

### *In vitro* drug treatment

Proliferating C2C12 myoblasts were seeded in 6-well plates (×4 wells per group). *In vitro* drug treatments began at 50%–60% confluence for C2C12 myoblasts and D7 stage for C2C12 myotubes. For metformin groups, they were treated with metformin hydrochloride (#PHR1084, Sigma-Aldrich) dissolved in phosphate buffered saline (PBS), pH 7.4 (#10010023, ThermoFisher) at concentrations of 0.3, 0.6, 1 and 2 mM for 24 h against a PBS control (0.1% v/v). For oxandrolone groups, they were treated with oxandrolone (#SML0437, Sigma-Aldrich) dissolved in ethanol absolute > 99.8% (#20821.296, VWR) at concentrations of 1, 10 and 100 μM for 24 h against an ethanol absolute > 99.8% vehicle control (0.1% v/v).

### Lactate dehydrogenase (LDH) assay

Drug cytotoxicity was measured by the lactate dehydrogenase (LDH)-Glo™ Cytotoxicity Assay Kit (#J2380, Promega) following manufacturer’s instructions. Luminescence was measured at 400 nm using the GloMax® Explorer Multimode Microplate Reader (#GM3500, Promega).

### Bromodeoxyuridine (BrDU) cell proliferation assay

Cell proliferation was measured by the Bromodeoxyuridine (BrDU) Cell Proliferation Assay Kit (#QIA58, Sigma-Aldrich) following manufacturer’s instructions. Absorbance was measured at 450–540 nm using the GloMax® Explorer Multimode Microplate Reader (#GM3500, Promega).

### Small interfering RNA-mediated *Smn* knockdown in C2C12 cells

A 10 μM *Smn* small interfering RNA (siRNA) (Duplex name: mm.RiSmn1.13.1) (Integrated DNA Technologies (IDT)) was used to knock down *Smn* levels against a 10 μM scrambled siRNA (scrambled DsiRNA, #51-01-19-08) (IDT) negative control. The *Smn* and scrambled siRNAs were aliquoted separately into an siRNA-lipofectamine complex containing Lipofectamine® RNAiMAX Reagent (#13778075, Invitrogen) and Opti-MEM (#31985062, Gibco) following manufacturer’s instructions. C2C12 myoblasts were transfected for 48 h with *Smn* depletion, while C2C12 myotubes were freshly transfected at differentiation (D) stages D0, D3 and D6 for 48 h with *Smn* depletion confirmed via quantitative polymerase chain reaction (qPCR) (Table S13).

### Serum starvation-induced canonical muscle atrophy in differentiated C2C12 myotubes

Differentiated C2C12 myotubes were incubated in serum-free glucose (4.5 g/l) and L-glutamine (0.6 g/l) DMEM (#BE12-741F, Lonza) with 1% Penicillin-Streptomycin (10 000 U/ml) (#15140122, Gibco) for 24 h. Atrophy was confirmed by upregulation of atrogenes *Atrogin-1* and *MuRF1* via qPCR (Table S13) and morphology via microscopy (Motic AE31E).

### RNA extraction and quantitative polymerase chain reaction (qPCR)

RNA extraction for C2C12 cells was performed with the ISOLATE II RNA Mini Kit (#BIO-52073, Meridian BIOSCIENCE) as per manufacturer’s instructions. Skeletal muscle (triceps and *Tibialis anterior* (TA)) and spinal cord tissue samples underwent homogenization with 7 mm stainless steel beads (#69990, Qiagen) in a Tissue Lyser LT (#85600, Qiagen) set at 60 oscillations/second for 2 min followed by microcentrifugation at > 10 000 RCF (MSE Sanyo Hawk 15/05) for 1 min. RNA extractions from harvested skeletal muscle was performed with the RNeasy Fibrous Tissue Kit (#74704, Qiagen) and all other harvested tissues with ISOLATE II RNA Mini Kit (#BIO-52073, Meridian BIOSCIENCE) as per manufacturer’s instructions. RNA concentrations (ng/μl) were measured with the NanoDrop 1000 spectrophotometer (ThermoScientific) before reverse transcription was performed using the qPCRBIO cDNA Synthesis Kit (#PB30.11-10, PCR Biosystems) as per manufacturer’s instructions.

The cDNA was then diluted by 1:5 in nuclease-free water (#10526945, ThermoFisher). qPCR was performed using 2× PCRBIO Sygreen Blue Mix Hi-ROX (#PB20.16-20, PCR Biosystems), nuclease-free water (#10526945, ThermoFisher) and 10 μM forward and reverse primers obtained from IDT (Table S13). The qPCR reactions were performed in the StepOnePlus™ Real-Time PCR System (ThermoScientific) with the following programme: initial denaturation (95°C for 2 min), 40 cycles of 95°C for 5 s and 60°C for 30 s and ending with melt curve stage (95°C for 15 s, 60°C for 1 min and 95°C for 15 s). Relative gene expression was quantified using the Pfaffl method [133] and referenced against the validated *RNA polymerase II polypeptide J* (*PolJ)* housekeeping gene [27, 134] (Table S13). Primer efficiency was calculated using LinRegPCR V11.0 [135].

### *C. elegans* drug treatments

The *C. elegans* (*C. elegans*) SMA strains used in this study was LM99 *smn-1(ok355)I/hT2(I;III)*, which was segregated into homozygotes *smn-1(ok355)*, lethal homozygotes *hT2/hT2* and control heterozygotes *smn-1(ok355)I/hT2* [91]. These animals were maintained at 20°C on Nematode Growth Medium (NGM) plates seeded with *Escherichia coli* OP50 bacteria [136]. *C. elegans* were treated by mixing metformin hydrochloride (#PHR1084, Sigma-Aldrich) dissolved in water at concentrations of 0, 1, 10 and 50 mM and oxandrolone (#SML0437, Sigma-Aldrich) dissolved in DMSO at concentrations of 0, 1, 10 and 50 μM with NGM agar solution.

### *C. elegans* neuromuscular assays

Neuromuscular assays were performed on day 3 animals raised on plates containing the pertinent solvent or drug. The pharyngeal pumping assay was performed as previously described [93]. Briefly, animals were filmed with a 150× objective using a AxioCam ICc5 camera at 175 frames/10 s on a Discovery.V8 SteREO microscope. Pumps were manually counted using the Zen Pro software v2.3. A pumping event was defined as a grinder movement in any axis. For locomotion assays animals were filmed with a 63× objective using a AxioCam ICc5 camara at 15 frames/sec on a Discovery.V8 SteREO microscope. Reversals, and paralysis time for 5 min (±SEM) were quantified using WormLab 1.1 software (MBF Bioscience). Final data represents three independent trials (n ≥ 25 animals in total per genotype).

### Statistical analyses

Statistical analyses were carried out using the most up to date GraphPad PRISM software. Prior to any analyses, outliers were identified via Grubb’s test (GraphPad) and subsequently removed. Appropriate statistical tests include unpaired t-test, one-way analysis of variance (ANOVA), and two-way ANOVA. Each post-hoc analyses used is noted in the respective figure legend. Kaplan-Meier survival curves were analysed with a log-rank test. Statistical significance was considered at *P* < 0.05, described in graphs as ^*^P < 0.05, ^*^^*^P < 0.01, ^*^^*^^*^P < 0.001 and ^*^^*^^*^^*^P < 0.0001.

## Supplementary Material

Supplementary_data_ddad192

## Data Availability

The datasets presented in this study can be found in the following online repository: NCBI BioProject, accession ID: PRJNA972323.

## References

[1] Kolb SJ, Kissel JT. Spinal muscular atrophy. Neurol Clin 2015;33:831–46. 26515624 10.1016/j.ncl.2015.07.004 PMC4628728

[2] Wojcik MH, Schwartz TS, Thiele KE. et al. Infant mortality: the contribution of genetic disorders. J Perinatol 2019;39:1611–9. 31395954 10.1038/s41372-019-0451-5 PMC6879816

[3] Lefebvre S, Bürglen L, Reboullet S. et al. Identification and characterization of a spinal muscular atrophy-determining gene. Cell 1995;80:155–65. 7813012 10.1016/0092-8674(95)90460-3

[4] Rodrigues NR, Owen N, Talbot K. et al. Deletions in the survival motor neuron gene on 5q13 in autosomal recessive spinal muscular atrophy. Hum Mol Genet 1995;4:631–4. 7633412 10.1093/hmg/4.4.631

[5] Coovert DD, Le TT, McAndrew PE. et al. The survival motor neuron protein in spinal muscular atrophy. Hum Mol Genet 1997;6:1205–14. 9259265 10.1093/hmg/6.8.1205

[6] Groen EJN, Perenthaler E, Courtney NL. et al. Temporal and tissue-specific variability of SMN protein levels in mouse models of spinal muscular atrophy. Hum Mol Genet 2018;27:2851–62. 29790918 10.1093/hmg/ddy195 PMC6077828

[7] Singh RN, Howell MD, Ottesen EW. et al. Diverse role of survival motor neuron protein. Biochim Biophys Acta 2017;1860:299–315. 10.1016/j.bbagrm.2016.12.008 PMC5325804 28095296

[8] Schrank B, Götz R, Gunnersen JM. et al. Inactivation of the survival motor neuron gene, a candidate gene for human spinal muscular atrophy, leads to massive cell death in early mouse embryos. Proc Natl Acad Sci 1997;94:9920–5. 9275227 10.1073/pnas.94.18.9920 PMC23295

[9] Monani UR, Sendtner M, Coovert DD. et al. The human centromeric survival motor neuron gene (SMN2) rescues embryonic lethality in Smn(−/−) mice and results in a mouse with spinal muscular atrophy. Hum Mol Genet 2000;9:333–9. 10655541 10.1093/hmg/9.3.333

[10] Rochette CF, Gilbert N, Simard LR. SMN gene duplication and the emergence of the SMN2 gene occurred in distinct hominids: SMN2 is unique to homo sapiens. Hum Genet 2001;108:255–66. 11354640 10.1007/s004390100473

[11] Lorson CL, Hahnen E, Androphy EJ. et al. A single nucleotide in the SMN gene regulates splicing and is responsible for spinal muscular atrophy. Proc Natl Acad Sci U S A 1999;96:6307–11. 10339583 10.1073/pnas.96.11.6307 PMC26877

[12] Lefebvre S, Burlet P, Liu Q. et al. Correlation between severity and SMN protein level in spinal muscular atrophy. Nat Genet 1997;16:265–9. 9207792 10.1038/ng0797-265

[13] Hua Y, Vickers TA, Okunola HL. et al. Antisense masking of an hnRNP A1/A2 Intronic splicing silencer corrects SMN2 splicing in transgenic mice. Am J Hum Genet 2008;82:834–48. 18371932 10.1016/j.ajhg.2008.01.014 PMC2427210

[14] Finkel RS, Mercuri E, Darras BT. et al. Nusinersen versus sham control in infantile-onset spinal muscular atrophy. N Engl J Med 2017;377:1723–32. 29091570 10.1056/NEJMoa1702752

[15] Poirier A, Weetall M, Heinig K. et al. Risdiplam distributes and increases SMN protein in both the central nervous system and peripheral organs. Pharmacol Res Perspect 2018;6:e00447. 30519476 10.1002/prp2.447 PMC6262736

[16] Servais L, Baranello G, Masson R. et al. FIREFISH part 2: efficacy and safety of risdiplam (RG7916) in infants with type 1 spinal muscular atrophy (SMA) (1302). Neurology 2020;94.

[17] Al-Zaidy SA, Kolb SJ, Lowes L. et al. AVXS-101 (Onasemnogene Abeparvovec) for SMA1: comparative study with a prospective natural history cohort. J Neuromuscul Dis 2019;6:307–17. 31381526 10.3233/JND-190403

[18] Mendell JR, Al-Zaidy S, Shell R. et al. Single-dose gene-replacement therapy for spinal muscular atrophy. N Engl J Med 2017;377:1713–22. 29091557 10.1056/NEJMoa1706198

[19] Mercuri E, Darras BT, Chiriboga CA. et al. Nusinersen versus sham control in later-onset spinal muscular atrophy. N Engl J Med 2018;378:625–35. 29443664 10.1056/NEJMoa1710504

[20] Dangouloff T, Servais L. Clinical evidence supporting early treatment of patients with spinal muscular atrophy: current perspectives. Ther Clin Risk Manag 2019;Volume 15:1153–61. 10.2147/TCRM.S172291 PMC6778729 31632042

[21] Bowerman M, Becker CG, Yáñez-Muñoz RJ. et al. Therapeutic strategies for spinal muscular atrophy: SMN and beyond. Dis Model Mech 2017;10:943–54. 28768735 10.1242/dmm.030148 PMC5560066

[22] Martínez-Hernández R, Bernal S, Alias L. et al. Abnormalities in early markers of muscle involvement support a delay in myogenesis in spinal muscular atrophy. J Neuropathol Exp Neurol 2014;73:559–67. 24806300 10.1097/NEN.0000000000000078

[23] Hayhurst M, Wagner AK, Cerletti M. et al. A cell-autonomous defect in skeletal muscle satellite cells expressing low levels of survival of motor neuron protein. Dev Biol 2012;368:323–34. 22705478 10.1016/j.ydbio.2012.05.037 PMC3851302

[24] Boyer JG, Murray LM, Scott K. et al. Early onset muscle weakness and disruption of muscle proteins in mouse models of spinal muscular atrophy. Skelet Muscle 2013;3:24. 24119341 10.1186/2044-5040-3-24 PMC3852932

[25] Stevens L, Bastide B, Maurage CA. et al. Childhood spinal muscular atrophy induces alterations in contractile and regulatory protein isoform expressions. Neuropathol Appl Neurobiol 2008;34:659–70. 18363640 10.1111/j.1365-2990.2008.00950.x

[26] Martínez-Hernández R, Soler-Botija C, Also E. et al. The developmental pattern of myotubes in spinal muscular atrophy indicates prenatal delay of muscle maturation. J Neuropathol Exp Neurol 2009;68:474–81. 19525895 10.1097/NEN.0b013e3181a10ea1

[27] Walter LM, Deguise M-O, Meijboom KE. et al. Interventions targeting glucocorticoid-Krüppel-like factor 15-branched-chain amino acid Signaling improve disease phenotypes in spinal muscular atrophy mice. EBioMedicine 2018;31:226–42. 29735415 10.1016/j.ebiom.2018.04.024 PMC6013932

[28] Place A . A phase 2 study to evaluate the efficacy and safety of SRK-015 in patients with later-onset spinal muscular atrophy (TOPAZ): an introduction (4534). Neurology 2020;94.

[29] Rudnicki SA, Andrews JA, Duong T. et al. Reldesemtiv in patients with spinal muscular atrophy: a phase 2 hypothesis-generating study. Neurotherapeutics 2021;18:1127–36. 33624184 10.1007/s13311-020-01004-3 PMC8423982

[30] Dickson M, Gagnon JP. The cost of new drug discovery and development. Discov Med 2004;4:172–9. 20704981

[31] Dangouloff T, Botty C, Beaudart C. et al. Systematic literature review of the economic burden of spinal muscular atrophy and economic evaluations of treatments. Orphanet J Rare Dis 2021;16:47. 33485382 10.1186/s13023-021-01695-7 PMC7824917

[32] Talevi A, Bellera CL. Challenges and opportunities with drug repurposing: finding strategies to find alternative uses of therapeutics. Expert Opin Drug Discovery 2020;15:397–401. 10.1080/17460441.2020.1704729 31847616

[33] Cruz-Topete D, Cidlowski JA. One hormone, two actions: anti- and pro-inflammatory effects of glucocorticoids. Neuroimmunomodulation 2015;22:20–32. 25227506 10.1159/000362724 PMC4243162

[34] Matthews E, Brassington R, Kuntzer T. et al. Corticosteroids for the treatment of Duchenne muscular dystrophy. Cochrane Database Syst Rev 2016;2016. 10.1002/14651858.CD003725.pub4 PMC8580515 27149418

[35] Ricotti V, Ridout DA, Scott E. et al. Long-term benefits and adverse effects of intermittent versus daily glucocorticoids in boys with Duchenne muscular dystrophy. J Neurol Neurosurg Psychiatry 2013;84:698–705. 23250964 10.1136/jnnp-2012-303902

[36] McMillan HJ . Intermittent glucocorticoid regimes for younger boys with duchenne muscular dystrophy: balancing efficacy with side effects. Muscle Nerve 2019;59:638–9. 30993732 10.1002/mus.26490

[37] Morrison-Nozik A, Anand P, Zhu H. et al. Glucocorticoids enhance muscle endurance and ameliorate Duchenne muscular dystrophy through a defined metabolic program. Proc Natl Acad Sci 2015;112:E6780–9. 26598680 10.1073/pnas.1512968112 PMC4679037

[38] Bulfield G, Siller WG, Wight PA. et al. X chromosome-linked muscular dystrophy (mdx) in the mouse. Proc Natl Acad Sci U S A 1984;81:1189–92. 6583703 10.1073/pnas.81.4.1189 PMC344791

[39] Oray M, Abu Samra K, Ebrahimiadib N. et al. Long-term side effects of glucocorticoids. Expert Opin Drug Saf 2016;15:457–65. 26789102 10.1517/14740338.2016.1140743

[40] Hsieh-Li HM, Chang JG, Jong YJ. et al. A mouse model for spinal muscular atrophy. Nat Genet 2000;24:66–70. 10615130 10.1038/71709

[41] Le TT, Pham LT, Butchbach MER. et al. SMNDelta7, the major product of the centromeric survival motor neuron (SMN2) gene, extends survival in mice with spinal muscular atrophy and associates with full-length SMN. Hum Mol Genet 2005;14:845–57. 15703193 10.1093/hmg/ddi078

[42] Meijboom KE, Volpato V, Monzón-Sandoval J. et al. Combining multiomics and drug perturbation profiles to identify muscle-specific treatments for spinal muscular atrophy. JCI Insight 2021;6:149446. 10.1172/jci.insight.149446 PMC8410072 34236053

[43] Deguise M-O, Boyer JG, McFall ER. et al. Differential induction of muscle atrophy pathways in two mouse models of spinal muscular atrophy. Sci Rep 2016;6:28846. 27349908 10.1038/srep28846 PMC4924104

[44] Simon CM, Dai Y, Van Alstyne M. et al. Converging mechanisms of p53 activation drive motor neuron degeneration in spinal muscular atrophy. Cell Rep 2017;21:3767–80. 29281826 10.1016/j.celrep.2017.12.003 PMC5747328

[45] Cerveró C, Montull N, Tarabal O. et al. Chronic treatment with the AMPK agonist AICAR prevents skeletal muscle pathology but fails to improve clinical outcome in a mouse model of severe spinal muscular atrophy. Neurotherapeutics 2016;13:198–216. 26582176 10.1007/s13311-015-0399-x PMC4720671

[46] Ripolone M, Ronchi D, Violano R. et al. Impaired muscle mitochondrial biogenesis and myogenesis in spinal muscular atrophy. JAMA Neurol 2015;72:666–75. 25844556 10.1001/jamaneurol.2015.0178 PMC4944827

[47] Walter LM, Koch CE, Betts CA. et al. Light modulation ameliorates expression of circadian genes and disease progression in spinal muscular atrophy mice. Hum Mol Genet 2018;27:3582–97. 29982483 10.1093/hmg/ddy249 PMC6168969

[48] Ng SY, Mikhail A, Ljubicic V. Mechanisms of exercise-induced survival motor neuron expression in the skeletal muscle of spinal muscular atrophy-like mice. J Physiol 2019;597:4757–78. 31361024 10.1113/JP278454 PMC6767691

[49] Sansa A, Hidalgo I, Miralles MP. et al. Spinal muscular atrophy autophagy profile is tissue-dependent: differential regulation between muscle and motoneurons. Acta Neuropathol Commun 2021;9:122. 34217376 10.1186/s40478-021-01223-5 PMC8254901

[50] Ahsan S, Drăghici S. Identifying significantly impacted pathways and putative mechanisms with iPathwayGuide. Curr Protoc Bioinformatics 2017;57:7.15.1–30. 10.1002/cpbi.24 28654712

[51] Cotto KC, Wagner AH, Feng Y-Y. et al. DGIdb 3.0: a redesign and expansion of the drug–gene interaction database. Nucleic Acids Res 2018;46:D1068–73. 29156001 10.1093/nar/gkx1143 PMC5888642

[52] Pin F, Couch ME, Bonetto A. Preservation of muscle mass as a strategy to reduce the toxic effects of cancer chemotherapy on body composition. Curr Opin Support Palliat Care 2018;12:420–6. 30124526 10.1097/SPC.0000000000000382 PMC6221433

[53] Farooq F, Abadía-Molina F, MacKenzie D. et al. Celecoxib increases SMN and survival in a severe spinal muscular atrophy mouse model via p38 pathway activation. Hum Mol Genet 2013;22:3415–24. 23656793 10.1093/hmg/ddt191

[54] Mack SG, Cook DJ, Dhurjati P. et al. Systems biology investigation of cAMP modulation to increase SMN levels for the treatment of spinal muscular atrophy. PLoS One 2014;9:e115473. 25514431 10.1371/journal.pone.0115473 PMC4267815

[55] Rojas LBA, Gomes MB. Metformin: an old but still the best treatment for type 2 diabetes. Diabetol Metab Syndr 2013;5:6. 23415113 10.1186/1758-5996-5-6 PMC3607889

[56] Orr R, Fiatarone Singh M. The anabolic androgenic steroid oxandrolone in the treatment of wasting and catabolic disorders: review of efficacy and safety. Drugs 2004;64:725–50. 15025546 10.2165/00003495-200464070-00004

[57] Kjøbsted R, Hingst JR, Fentz J. et al. AMPK in skeletal muscle function and metabolism. FASEB J 2018;32:1741–77. 29242278 10.1096/fj.201700442R PMC5945561

[58] Soliman A, De Sanctis V, Alaaraj N. et al. The clinical application of metformin in children and adolescents: a short update. Acta Biomed 2020;91:e2020086. 32921782 10.23750/abm.v91i3.10127 PMC7717009

[59] Hafner P, Bonati U, Klein A. et al. Effect of combination l-Citrulline and metformin treatment on motor function in patients with Duchenne muscular dystrophy: a randomized clinical trial. JAMA Netw Open 2019;2:e1914171. 31664444 10.1001/jamanetworkopen.2019.14171 PMC6824222

[60] Fontes-Oliveira CC, Soares Oliveira BM, Körner Z. et al. Effects of metformin on congenital muscular dystrophy type 1A disease progression in mice: a gender impact study. Sci Rep 2018;8:16302. 30389963 10.1038/s41598-018-34362-2 PMC6214987

[61] Birk JB, Wojtaszewski JFP. Predominant α2/β2/γ3 AMPK activation during exercise in human skeletal muscle. J Physiol 2006;577:1021–32. 17038425 10.1113/jphysiol.2006.120972 PMC1890393

[62] Zhang L, Zhang Y, Zhou M. et al. Role and mechanism underlying FoxO6 in skeletal muscle in vitro and in vivo. Int J Mol Med 2021;48:143. 34080654 10.3892/ijmm.2021.4976

[63] Woschitz V, Mei I, Hedlund E. et al. Mouse models of SMA show divergent patterns of neuronal vulnerability and resilience. Skelet Muscle 2022;12:22. 36089582 10.1186/s13395-022-00305-9 PMC9465884

[64] Everaert C, Luypaert M, Maag JLV. et al. Benchmarking of RNA-sequencing analysis workflows using whole-transcriptome RT-qPCR expression data. Sci Rep 2017;7:1559. 28484260 10.1038/s41598-017-01617-3 PMC5431503

[65] Shafey D, Côté PD, Kothary R. Hypomorphic Smn knockdown C2C12 myoblasts reveal intrinsic defects in myoblast fusion and myotube morphology. Exp Cell Res 2005;311:49–61. 16219305 10.1016/j.yexcr.2005.08.019

[66] Millino C, Fanin M, Vettori A. et al. Different atrophy-hypertrophy transcription pathways in muscles affected by severe and mild spinal muscular atrophy. BMC Med 2009;7:14. 19351384 10.1186/1741-7015-7-14 PMC2676312

[67] Calura E, Cagnin S, Raffaello A. et al. Meta-analysis of expression signatures of muscle atrophy: gene interaction networks in early and late stages. BMC Genomics 2008;9:630. 19108710 10.1186/1471-2164-9-630 PMC2642825

[68] Rivera ME, Lyon ES, Vaughan RA. Effect of metformin on myotube BCAA catabolism. J Cell Biochem 2020;121:816–27. 31385363 10.1002/jcb.29327

[69] Bowerman M, Murray LM, Beauvais A. et al. A critical smn threshold in mice dictates onset of an intermediate spinal muscular atrophy phenotype associated with a distinct neuromuscular junction pathology. Neuromuscul Disord 2012;22:263–76. 22071333 10.1016/j.nmd.2011.09.007

[70] Bowerman M, Swoboda KJ, Michalski J-P. et al. Glucose metabolism and pancreatic defects in spinal muscular atrophy. Ann Neurol 2012;72:256–68. 22926856 10.1002/ana.23582 PMC4334584

[71] Deguise M-O, Baranello G, Mastella C. et al. Abnormal fatty acid metabolism is a core component of spinal muscular atrophy. Ann Clin Transl Neurol 2019;6:1519–32. 31402618 10.1002/acn3.50855 PMC6689695

[72] Boyer JG, Deguise M-O, Murray LM. et al. Myogenic program dysregulation is contributory to disease pathogenesis in spinal muscular atrophy. Hum Mol Genet 2014;23:4249–59. 24691550 10.1093/hmg/ddu142 PMC4103674

[73] Bruce AK, Jacobsen E, Dossing H. et al. Hypoglycaemia in spinal muscular atrophy. Lancet 1995;346:609–10. 7651007 10.1016/s0140-6736(95)91439-0

[74] Djordjevic SA, Milic-Rasic V, Brankovic V. et al. Glucose and lipid metabolism disorders in children and adolescents with spinal muscular atrophy types 2 and 3. Neuromuscul Disord 2021;31:291–9. 33685840 10.1016/j.nmd.2021.02.002

[75] Birnbaum MJ . Identification of a novel gene encoding an insulin-responsive glucose transporter protein. Cell 1989;57:305–15. 2649253 10.1016/0092-8674(89)90968-9

[76] DeWaal D, Nogueira V, Terry AR. et al. Hexokinase-2 depletion inhibits glycolysis and induces oxidative phosphorylation in hepatocellular carcinoma and sensitizes to metformin. Nat Commun 2018;9:446. 29386513 10.1038/s41467-017-02733-4 PMC5792493

[77] Owen MR, Doran E, Halestrap AP. Evidence that metformin exerts its anti-diabetic effects through inhibition of complex 1 of the mitochondrial respiratory chain. Biochem J 2000;348:607–14. 10839993 PMC1221104

[78] Graham GG, Punt J, Arora M. et al. Clinical pharmacokinetics of metformin. Clin Pharmacokinet 2011;50:81–98. 21241070 10.2165/11534750-000000000-00000

[79] Łabuzek K, Suchy D, Gabryel B. et al. Quantification of metformin by the HPLC method in brain regions, cerebrospinal fluid and plasma of rats treated with lipopolysaccharide. Pharmacol Rep 2010;62:956–65. 21098880 10.1016/s1734-1140(10)70357-1

[80] Singh J, Olle B, Suhail H. et al. Metformin-induced mitochondrial function and ABCD2 up-regulation in X-linked adrenoleukodystrophy involves AMP-activated protein kinase. J Neurochem 2016;138:86–100. 26849413 10.1111/jnc.13562

[81] Fenichel G, Pestronk A, Florence J. et al. A beneficial effect of oxandrolone in the treatment of Duchenne muscular dystrophy: a pilot study. Neurology 1997;48:1225–6. 9153447 10.1212/wnl.48.5.1225

[82] Hart DW, Wolf SE, Ramzy PI. et al. Anabolic effects of oxandrolone after severe burn. Ann Surg 2001;233:556–64. 11303139 10.1097/00000658-200104000-00012 PMC1421286

[83] Ren H, Yin P, Duan C. IGFBP-5 regulates muscle cell differentiation by binding to IGF-II and switching on the IGF-II auto-regulation loop. J Cell Biol 2008;182:979–91. 18762576 10.1083/jcb.200712110 PMC2528583

[84] Ganassi M, Badodi S, Wanders K. et al. Myogenin is an essential regulator of adult myofibre growth and muscle stem cell homeostasis. elife 2020;9:e60445. 33001028 10.7554/eLife.60445 PMC7599067

[85] Cai D, Dhe-Paganon S, Melendez PA. et al. Two new substrates in insulin signaling, IRS5/DOK4 and IRS6/DOK5. J Biol Chem 2003;278:25323–30. 12730241 10.1074/jbc.M212430200

[86] Lee S-W, Won J-Y, Yang J. et al. AKAP6 inhibition impairs myoblast differentiation and muscle regeneration: positive loop between AKAP6 and myogenin. Sci Rep 2015;5:16523. 26563778 10.1038/srep16523 PMC4643297

[87] Wu Y, Zhao W, Zhao J. et al. REDD1 is a major target of testosterone action in preventing dexamethasone-induced muscle loss. Endocrinology 2010;151:1050–9. 20032058 10.1210/en.2009-0530 PMC2840688

[88] Mirza KA, Pereira SL, Edens NK. et al. Attenuation of muscle wasting in murine C2C 12 myotubes by epigallocatechin-3-gallate. J Cachexia Sarcopenia Muscle 2014;5:339–45. 24647719 10.1007/s13539-014-0139-9 PMC4248406

[89] Zeman RJ, Bauman WA, Wen X. et al. Improved functional recovery with oxandrolone after spinal cord injury in rats. Neuroreport 2009;20:864–8. 19424096 10.1097/WNR.0b013e32832c5cc2

[90] Ahmad A, Herndon DN, Szabo C. Oxandrolone protects against the development of multiorgan failure, modulates the systemic inflammatory response and promotes wound healing during burn injury. Burns 2019;45:671–81. 31018913 10.1016/j.burns.2018.10.006

[91] Briese M, Esmaeili B, Fraboulet S. et al. Deletion of smn-1, the Caenorhabditis elegans ortholog of the spinal muscular atrophy gene, results in locomotor dysfunction and reduced lifespan. Hum Mol Genet 2009;18:97–104. 18829666 10.1093/hmg/ddn320 PMC2644645

[92] Bertrandy S, Burlet P, Clermont O. et al. The RNA-binding properties of SMN: deletion analysis of the zebrafish orthologue defines domains conserved in evolution. Hum Mol Genet 1999;8:775–82. 10196366 10.1093/hmg/8.5.775

[93] Dimitriadi M, Derdowski A, Kalloo G. et al. Decreased function of survival motor neuron protein impairs endocytic pathways. Proc Natl Acad Sci U S A 2016;113:E4377–86. 27402754 10.1073/pnas.1600015113 PMC4968725

[94] Nilsson EC, Long YC, Martinsson S. et al. Opposite transcriptional regulation in skeletal muscle of AMP-activated protein kinase gamma3 R225Q transgenic versus knock-out mice. J Biol Chem 2006;281:7244–52. 16410251 10.1074/jbc.M510461200

[95] Barnes BR, Long YC, Steiler TL. et al. Changes in exercise-induced gene expression in 5’-AMP-activated protein kinase gamma3-null and gamma3 R225Q transgenic mice. Diabetes 2005;54:3484–9. 16306365 10.2337/diabetes.54.12.3484

[96] Marangos PJ, Loftus T, Wiesner J. et al. Adenosinergic modulation of homocysteine-induced seizures in mice. Epilepsia 1990;31:239–46. 2344840 10.1111/j.1528-1157.1990.tb05371.x

[97] Corton JM, Gillespie JG, Hawley SA. et al. 5-aminoimidazole-4-carboxamide ribonucleoside. A specific method for activating AMP-activated protein kinase in intact cells? Eur J Biochem 1995;229:558–65. 7744080 10.1111/j.1432-1033.1995.tb20498.x

[98] Xiao B, Heath R, Saiu P. et al. Structural basis for AMP binding to mammalian AMP-activated protein kinase. Nature 2007;449:496–500. 17851531 10.1038/nature06161

[99] Thelen MP, Wirth B, Kye MJ. Mitochondrial defects in the respiratory complex I contribute to impaired translational initiation via ROS and energy homeostasis in SMA motor neurons. Acta Neuropathol Commun 2020;8:223. 33353564 10.1186/s40478-020-01101-6 PMC7754598

[100] Borkowska A, Jankowska A, Szlagatys-Sidorkiewicz A. et al. Coexistence of type 1 diabetes mellitus and spinal muscular atrophy in an 8-year-old girl: a case report. Acta Biochim Pol 2015;62:167–8. 25669159 10.18388/abp.2014_883

[101] Hossain S, Chao J. MON-154 abnormal glucose homeostasis in spinal muscular atrophy (SMA) leading to a transient episode of diabetic ketoacidosis (DKA). J Endocr Soc 2019;3:MON-154.

[102] Sun J, Harrington MA, Porter B. Sex difference in spinal muscular atrophy patients – are males more vulnerable? J Neuromuscul Dis 2023;10:847–67. 37393514 10.3233/JND-230011 PMC10578261

[103] MacLean HE, Chiu WSM, Notini AJ. et al. Impaired skeletal muscle development and function in male, but not female, genomic androgen receptor knockout mice. *FASEB*. *J* 2008;22:2676–89. 18390925 10.1096/fj.08-105726

[104] Morton RW, Sato K, Gallaugher MPB. et al. Muscle androgen receptor content but not systemic hormones is associated with resistance training-induced skeletal muscle hypertrophy in healthy, young men. Front Physiol 2018;9. 10.3389/fphys.2018.01373 PMC6189473 30356739

[105] Liu Y, Beyer A, Aebersold R. On the dependency of cellular protein levels on mRNA abundance. Cell 2016;165:535–50. 27104977 10.1016/j.cell.2016.03.014

[106] Knorre DG, Kudryashova NV, Godovikova TS. Chemical and functional aspects of posttranslational modification of proteins. Acta Nat 2009;1:29–51. PMC3347534 22649613

[107] Kessler T, Latzer P, Schmid D. et al. Cerebrospinal fluid proteomic profiling in nusinersen-treated patients with spinal muscular atrophy. J Neurochem 2020;153:650–61. 31903607 10.1111/jnc.14953

[108] Gardell SJ, Zhang X, Kapoor N. et al. Metabolomics analyses of muscle atrophy induced by hind limb unloading. Methods Mol Biol 2019;1996:297–309. 31127563 10.1007/978-1-4939-9488-5_22

[109] Finkel RS, Crawford TO, Swoboda KJ. et al. Candidate proteins, metabolites and transcripts in the biomarkers for spinal muscular atrophy (BforSMA) clinical study. PLoS One 2012;7:e35462. 22558154 10.1371/journal.pone.0035462 PMC3338723

[110] De Micheli AJ, Spector JA, Elemento O. et al. A reference single-cell transcriptomic atlas of human skeletal muscle tissue reveals bifurcated muscle stem cell populations. Skelet Muscle 2020;10:19. 32624006 10.1186/s13395-020-00236-3 PMC7336639

[111] Williams K, Yokomori K, Mortazavi A. Heterogeneous skeletal muscle cell and nucleus populations identified by single-cell and single-nucleus resolution transcriptome assays. Front Genet 2022;13. 10.3389/fgene.2022.835099 PMC9136090 35646075

[112] Habets LE, Bartels B, Asselman F-L. et al. Magnetic resonance reveals mitochondrial dysfunction and muscle remodelling in spinal muscular atrophy. Brain 2022;145:1422–35. 34788410 10.1093/brain/awab411 PMC9128825

[113] Saleh KK, Xi H, Switzler C. et al. Single cell sequencing maps skeletal muscle cellular diversity as disease severity increases in dystrophic mouse models. iScience 2022;25:105415. 36388984 10.1016/j.isci.2022.105415 PMC9646951

[114] Chemello F, Wang Z, Li H. et al. Degenerative and regenerative pathways underlying Duchenne muscular dystrophy revealed by single-nucleus RNA sequencing. Proc Natl Acad Sci 2020;117:29691–701. 33148801 10.1073/pnas.2018391117 PMC7703557

[115] Nguyen AT, Armstrong EA, Yager JY. Neurodevelopmental reflex testing in neonatal rat pups. J Vis Exp 2017;122:55261. 10.3791/55261 PMC5565095 28518104

[116] Afgan E, Baker D, Batut B. et al. The galaxy platform for accessible, reproducible and collaborative biomedical analyses: 2018 update. Nucleic Acids Res 2018;46:W537–44. 29790989 10.1093/nar/gky379 PMC6030816

[117] Babraham Bioinformatics . FastQC A Quality Control tool for High Throughput Sequence Data. https://www.bioinformatics.babraham.ac.uk/projects/fastqc/ (accessed Apr 25, 2022).

[118] Bolger AM, Lohse M, Usadel B. Trimmomatic: a flexible trimmer for Illumina sequence data, *Bioinformatics* 2014;30:2114–2120. 10.1093/bioinformatics/btu170 PMC4103590 24695404

[119] FASTX-Toolkit http://hannonlab.cshl.edu/fastx_toolkit/ (accessed Apr 25, 2022).

[120] Kim D, Paggi JM, Park C. et al. Graph-based genome alignment and genotyping with HISAT2 and HISAT-genotype. Nat Biotechnol 2019;37:907–15. 31375807 10.1038/s41587-019-0201-4 PMC7605509

[121] Liao Y, Smyth GK, Shi W. featureCounts: an efficient general purpose program for assigning sequence reads to genomic features. Bioinformatics 2014;30:923–30. 24227677 10.1093/bioinformatics/btt656

[122] Ewels P, Magnusson M, Lundin S. et al. MultiQC: summarize analysis results for multiple tools and samples in a single report. Bioinformatics 2016;32:3047–8. 27312411 10.1093/bioinformatics/btw354 PMC5039924

[123] Love MI, Huber W, Anders S. Moderated estimation of fold change and dispersion for RNA-seq data with DESeq2. Genome Biol 2014;15:550. 25516281 10.1186/s13059-014-0550-8 PMC4302049

[124] Kanehisa M, Furumichi M, Tanabe M. et al. KEGG: new perspectives on genomes, pathways, diseases and drugs. Nucleic Acids Res 2017;45:D353–61. 27899662 10.1093/nar/gkw1092 PMC5210567

[125] Draghici S, Khatri P, Martins RP. et al. Global functional profiling of gene expression. Genomics 2003;81:98–104. 12620386 10.1016/s0888-7543(02)00021-6

[126] Jantzen SG, Sutherland BJ, Minkley DR. et al. GO trimming: systematically reducing redundancy in large gene ontology datasets. BMC Res Notes 2011;4:267. 21798041 10.1186/1756-0500-4-267 PMC3160396

[127] The Gene Ontology Consortium . The gene ontology resource: 20 years and still going strong. Nucleic Acids Res 2019;47:D330–8. 30395331 10.1093/nar/gky1055 PMC6323945

[128] Lu C, Wu C, Ghoreishi D. et al. OPLS4: improving force field accuracy on challenging regimes of chemical space. J Chem Theory Comput 2021;17:4291–300. 34096718 10.1021/acs.jctc.1c00302

[129] Berman HM, Westbrook J, Feng Z. et al. The protein data bank. Nucleic Acids Res 2000;28:235–42. 10592235 10.1093/nar/28.1.235 PMC102472

[130] Sastry GM, Adzhigirey M, Day T. et al. Protein and ligand preparation: parameters, protocols, and influence on virtual screening enrichments. J Comput Aided Mol Des 2013;27:221–34. 23579614 10.1007/s10822-013-9644-8

[131] Friesner RA, Murphy RB, Repasky MP. et al. Extra precision glide: docking and scoring incorporating a model of hydrophobic enclosure for protein-ligand complexes. J Med Chem 2006;49:6177–96. 17034125 10.1021/jm051256o

[132] Yaffe D, Saxel O. Serial passaging and differentiation of myogenic cells isolated from dystrophic mouse muscle. Nature 1977;270:725–7. 563524 10.1038/270725a0

[133] Pfaffl MW . A new mathematical model for relative quantification in real-time RT-PCR. Nucleic Acids Res 2001;29:e45. 11328886 10.1093/nar/29.9.e45 PMC55695

[134] Radonić A, Thulke S, Mackay IM. et al. Guideline to reference gene selection for quantitative real-time PCR. Biochem Biophys Res Commun 2004;313:856–62. 14706621 10.1016/j.bbrc.2003.11.177

[135] Untergasser A, Ruijter JM, Benes V. et al. Web-based LinRegPCR: application for the visualization and analysis of (RT)-qPCR amplification and melting data. BMC Bioinformatics 2021;22:398. 34433408 10.1186/s12859-021-04306-1 PMC8386043

[136] Brenner S . The genetics of Caenorhabditis elegans. Genetics 1974;77:71–94. 4366476 10.1093/genetics/77.1.71 PMC1213120

