# Supplementary material for: A transcriptomics-based drug repositioning approach to identify drugs with similar activities for the treatment of muscle pathologies in spinal muscular atrophy (SMA) models
Source: Hum Mol Genet. 2023 Nov 8;33(5):400–25. doi: 10.1093/hmg/ddad192 (PMC10877467; doi:10.1093/hmg/ddad192)
Supplement: Supplementary_data_ddad192 [file supplementary_data_ddad192.zip › Supplementary_data_ddad192/SUPPLEMENTARY FIGURE AND TABLE LEGENDS.docx]

**SUPPLEMENTARY FIGURE LEGENDS**

**Figure S1. “Condition” and “Treatment” groups share distinct transcriptomic patterns for a specific sub-set of genes in prednisolone-treated *Smn^-/-^;SMN2* SMA mice and untreated *Smn^-/-^;SMN2* SMA and *Smn^+/-^;SMN2* healthy mice.**

*Smn^-/-^;SMN2* SMA and *Smn^+/-^;SMN2* healthy mice received prednisolone treatment (5 mg/kg gavage every 2 days) from P0. The triceps was harvested from P7 untreated and prednisolone-treated *Smn^-/-^;SMN2* SMA and *Smn^+/-^;SMN2* healthy mice for RNA isolation and library preparation for RNA-Sequencing. Differential gene expression analysis was performed by DESeq2 v2.11.40.2 with study design set to “condition and “treatment”. Principal component analysis based on transcriptomic profiles between **a.** P7 untreated *Smn^+/-^;SMN2* (red, n=3) and untreated *Smn^-/-^;SMN2* (blue, n=3) mice. **b.** P7 untreated *Smn^-/-^;SMN2* (green, n=3) and prednisolone-treated *Smn^-/-^;SMN2* (blue, n=2) SMA mice**.**

**Figure S2. Molecular docking identifies the majority of the predicted drug-gene target interactions as viable matches.**

Binding of the co-crystallized compounds (green) and predicted binding of the drug molecules (orange) to their suggested targets. Glutamate (A) and acamprosate (B) in Grin1 active site; retinoic acid (C) and acitretin (D) in RarB active site; testosterone (E) and nandrolone (F) in AR active site; estriol (G) progesterone (H), and tibolone (I) in Esr1 active site; THC (J) and cannabidiol (K) in Cnr1 active site; a selective Pdpk1 inhibitor (L) and celecoxib (M) in Pdpk1 active site. Ligands are shown in colour ball-and-stick representation with molecular surface rendered, amino acid residues as gray sticks and binding interactions as colour dashed-lines.

**Figure S3. Bioinformatic identification of shared target genes between metformin and prednisolone in SMA skeletal muscle.**

**a.** The pathway diagram contains proteins within the FOXO signalling pathway (KEGG: 04068) encoded by the predicted differentially expressed genes from prednisolone vs untreated *Smn^-/-^;SMN2* SMA skeletal muscle. The AMPK protein (yellow circle) represents the AMPK-γ3 isoform gene *Prkag3*. Its activity on the iPathwayGuide interactive server highlights that it can be targeted by metformin via a built-in KEGG Drugs database. The red lines represent coherent cascades, which supports the consistency of the transcriptomic data and published pathway activity. In the FOXO signalling pathway, activation of AMPK represented by *Prkag3* coherently downregulates the FOXO protein (green circle), which represents *Foxo1*, *Foxo3*, *Foxo4*, and *Foxo6* isoforms. The highest logFC patterns are shown in dark red and lowest in dark blue as indicated in the legend value box. Graph generated in iPathwayGuide (Advaita). **b.** Differential gene expression pattern by logFC (Y-axis) of predicted metformin target genes *Prkag3*, *Foxo1*, *Foxo3*, *Foxo4* and *Foxo6* based on transcriptomic data from prednisolone vs untreated *Smn^-/-^;SMN2* SMA skeletal muscle. Upregulated genes above X-axis are highlighted in red and downregulated genes below X-axis are highlighted in blue. The box and whisker plot on Y-axis represents 1^st^ quartile, median and 3^rd^ quartile. Graph generated in iPathwayGuide (Advaita). **c.** Heatmap visualization for predicted metformin targets *Prkag3*, *Foxo1*, *Foxo3*, *Foxo4* and *Foxo6* (Log2FC > 0.6; FDR < 0.05) between untreated *Smn^+/-^;SMN2* healthy mice (left), untreated *Smn^-/-^;SMN2* SMA mice (centre) and prednisolone-treated *Smn^-/-^;SMN2* SMA mice (right). Colour key represents log2FC for upregulated (red) and downregulated (blue) genes. Heatmap was generated by Heatmap v2.1.1+galaxy1.

**Figure S4. Smn knockdown in C2C12 myoblasts and myotubes via *Smn* siRNA transfection.**

*Smn* siRNA knockdown (red) was performed for **a.** 48 hours in C2C12 myoblasts and **b.** every 48 hours throughout differentiation in D8 C2C12 myotubes. *Smn* knockdown of mRNA levels was confirmed by qPCR and compared to non-transfected (black) and scrambled siRNA control (blue) groups. Data are shown as scatter graph that represent mean ± SEM error bars; n = 4 samples per group across two independent experiments. One-way ANOVA followed by Tukey’s multiple comparisons test. C2C12 myoblasts F = 22.01; D8 C2C12 myotubes F = 115.4. *****p* <0.0001.

**Figure S5.** **Both physiological and supraphysiological metformin concentrations affect the expression of specific predicted target genes in C2C12 myoblasts and myotubes.**

**a.** C2C12 myoblasts and **b.** D8 C2C12 myotubes were treated with a range of physiological (30 μM (red) and 60 μM (green)) and supraphysiological (1 mM (brown) and 2 mM (blue)) metformin concentrations for 24 hours against a PBS vehicle control (black) to evaluate the mRNA expression via qPCR of predicted target genes *Prkag3*, *Foxo1*, *Foxo3*, *Foxo4* and *Foxo6*. Data are shown as bar charts with scatter graph that represent mean ± SEM error bars; n = 4 samples per group across two independent experiments. Two-way ANOVA followed by uncorrected Fisher’s least significant difference (LSD). C2C12 myoblasts F = 7.822; D8 C2C12 myotubes F = 12.17. **p* <0.05, ***p* <0.01, ****p* <0.001, *****p* <0.0001.

**Figure S6. 0.9% saline vehicle treatment had no effect on survival or phenotype in *Smn^2B/-^* SMA and *Smn^2B/+^* healthy mice.**

All treated animals received a daily dose of 0.9% physiological saline vehicle by gavage starting at P5**. a.** Survival curves of untreated (n = 13, black) and 0.9% saline vehicle-treated (n = 14, green) *Smn^2B/-^* SMA mice. Kaplan-Meier survival curve shown with log rank (Mantel-Cox) test, ns = not significant, *p* = 0.0653. **b.** Daily weights of untreated (n = 13, black) and 0.9% saline vehicle-treated (n = 14, green) *Smn^2B/-^* SMA mice. Data represented as mean ± SEM error bars; Two-way ANOVA followed by a Sidak’s multiple comparison test, F = 454.6, df = 501 **c.** Daily righting reflex test for motor function activity up to a 30 second maximum time point in untreated (n = 13, black) and 0.9% saline vehicle-treated (n = 14, green) *Smn^2B/-^* SMA mice. Data are shown as bar chart with mean ± SEM error bars; unpaired T-test, ns = not significant, *p* = 0.6626**. d.** Survival curves of untreated (n = 16, black) and 0.9% saline vehicle-treated (n = 22, green) *Smn^2B/+^* healthy mice. Kaplan-Meier survival curve shown with log rank (Mantel-Cox) test, ns = not significant, *p* > 0.9999. **e.** Daily weights of untreated (n = 16, black) and 0.9% saline vehicle-treated (n = 22, green) *Smn^2B/+^* healthy mice. Data represented as mean ± SEM error bars; Two-way ANOVA followed by a Sidak’s multiple comparison test, F =328, df = 757. **f.** Daily righting reflex test for motor function activity up to a 30 second maximum time point in untreated (n = 16, black) and 0.9% saline vehicle-treated (n = 22, green) *Smn^2B/+^* healthy mice. Data are shown as bar chart with mean ± SEM error bars; unpaired T-test, ns = not significant, *p* = 0.9555**.**

**Figure S7. 200 and 400 mg/kg/day metformin had no negative effect on survival or phenotype in healthy *Smn^2B/+^* mice.**

All treated animals received a daily dose of metformin (either 200 or 400 mg/kg/day, diluted in 0.9% saline) by gavage starting at P5. **a.** Survival curves of untreated (n = 16, black) and 200 mg/kg/day metformin-treated (n = 20, red) *Smn^2B/+^* healthy mice. Kaplan-Meier survival curve shown with log rank (Mantel-Cox) test, ns = not significant, *p* > 0.9999. **b.** Daily weights of untreated (n = 16, black) and 200 mg/kg/day metformin-treated (n = 20, red) *Smn^2B/+^* healthy mice. Data represented as mean ± SEM error bars; Two-way ANOVA followed by a Sidak’s multiple comparison test, F = 549.5, df = 680. **c.** Daily righting reflex test for motor function activity up to a 30 second maximum time point in untreated (n = 16, black) and 200 mg/kg/day metformin-treated (n = 20, red) *Smn^2B/+^* healthy mice. Data are shown as bar chart with mean ± SEM error bars; unpaired T-test, ns = not significant, *p* = 0.9183**. d.** Survival curves of untreated (n = 16, black) and 400 mg/kg/day metformin-treated (n = 15, blue) *Smn^2B/+^* healthy mice. Kaplan-Meier survival curve shown with log rank (Mantel-Cox) test, ns = not significant, *p* > 0.9999. **e.** Daily weights of untreated (n = 16, black) and 400 mg/kg/day metformin-treated (n = 15, blue) *Smn^2B/+^* healthy mice. Data represented as mean ± SEM error bars; Two-way ANOVA followed by a Sidak’s multiple comparison test, F = 261.9, df = 435. **f.** Daily righting reflex test for motor function activity up to a 30 second maximum time point in untreated (n = 16, black) and 400 mg/kg/day metformin-treated (n = 15, blue) *Smn^2B/+^* healthy mice. Data are shown as bar chart with mean ± SEM error bars; unpaired T-test, ns = not significant, *p* = 0.9966**.**

**Figure S8. Oxandrolone is predicted to emulate the target patterns of prednisolone in the skeletal muscle (Triceps) of *Smn^-/-^;SMN2* SMA mice.**

**a.** Predicted model based on upstream regulator patterns predicted by iPathwayGuide in prednisolone vs untreated *Smn^-/-^;SMN2* SMA skeletal muscle. *Ar* upregulates downstream targets *Igfbp5* and *MyoG*, whilst it downregulates *Ddit4*. *Igfbp5* upregulates *Dok5* and *MyoG* upregulates *Akap6*. Upregulated genes shaded in red, downregulated genes shaded in blue. Downregulation of *Ddit4* based on previous published literature. **b.** Differential gene expression pattern by logFC (Y-axis) of predicted oxandrolone targets *Ddit4*, *Igfbp5*, *Ar*, *MyoG*, *Akap6* and *Dok5* based on transcriptomic data from prednisolone vs untreated *Smn^-/-^;SMN2* SMA skeletal muscle. Upregulated genes above X-axis are highlighted in red and downregulated genes below X-axis are highlighted in blue. The box and whisker plot on Y-axis represents 1^st^ quartile, median and 3^rd^ quartile. Graph generated in iPathwayGuide (Advaita). **c.** Heatmap visualization for predicted oxandrolone targets *Ar*, *MyoG*, *Igfbp5*, *Dok5*, *Akap6* and *Ddit4* (log2FC > 0.6; FDR < 0.05) between untreated *Smn^+/-^;SMN2* healthy mice (left), untreated *Smn^-/-^;SMN2* SMA mice (centre) and prednisolone-treated *Smn^-/-^;SMN2* SMA mice (right). Colour key represents log2FC for upregulated (red) and downregulated (blue) genes. Heatmap was generated by Heatmap v2.1.1+galaxy1.

**Figure S9. Low 1 μM oxandrolone treatment is non-toxic and does not impact proliferation in C2C12 myoblasts and myotubes.**

**a.** C2C12 myoblasts and **b.** D5 C2C12 myotubes were treated with 1 μM oxandrolone for 24 (purple) and 72 hours (orange) and compared to an absolute ethanol vehicle (24 hours red, 72 hours blue) in addition to untreated (black) and 1% Triton-X max lactate dehydrogenase (LDH) control (green). LDH levels in cell culture supernatant were measured by proportional fluorescence absorption readings (nm). C2C12 myoblasts were treated with 1 μM oxandrolone for **c.** 24 (orange) and **d.** 72 hours (purple) against an absolute ethanol vehicle (24 hours red, 72 hours blue) in addition to blank media (brown), blank cells (cyan), and untreated cells (black) controls. Absorption readings (nm) were measured from anti-BrDU antibody immunostained samples. Data are shown as bar charts that represent mean ± SEM error bars; n = 6 samples per group across one independent experiments; one-way ANOVA followed by Dunnett’s multiple comparisons test, **a.** F = 44.25, **b.** F = 3.092, **c.** F = 67.51, **d.** F = 64.71, **p* < 0.05, ****p* < 0.001, *****p* < 0.0001, ns = not significant.

**Figure S10. D5 stage in C2C12 myotubes for oxandrolone to elicit an *Ar* gene response.**

**a.** C2C12 myoblasts were treated with a range of oxandrolone concentrations of 1 (blue), 10 (red) and 100 μM (green) for 24 hours and compared to an absolute ethanol (vehicle, black) to evaluate the mRNA expression via qPCR of predicted target genes *Ar*, *Dok5*, *Igfbp5*, *Akap6*, *MyoG* and *Ddit4*. Data are shown as a bar chart and scatter graph that represent mean ± SEM error bars; n = 4 samples per group across two independent experiments. Two-way ANOVA followed by uncorrected Fisher’s least significant difference (LSD), F = 1.693. **p* <0.05, ***p* <0.01, ****p* <0.001, *****p* <0.0001. **b.** D8 C2C12 myotubes were treated with a range of oxandrolone concentrations of 1 (blue), 10 (red) and 100 μM (green) for 24 hours and compared to an absolute ethanol (vehicle, black) to evaluate the mRNA via qPCR of predicted target gene *Ar* and downstream targets *Igfbp5* and *MyoG*. Data are shown as bar chart and scatter graph that represent mean ± SEM error bars; n = 4 samples per group across two independent experiments. Two-way ANOVA followed by uncorrected Fisher’s least significant difference (LSD), F = 0.8708. **c.** D3 and **d.** D5 C2C12 myotubes were treated with 1 μM oxandrolone for 24 hours (blue) against an absolute ethanol (vehicle, black) to evaluate the mRNA levels via qPCR of *Ar*. Data are shown as scatter graphs that represent mean ± SEM error bars; n = 4 samples per group across one independent experiment; unpaired T-test D3 C2C12 myotubes *p* = 0.0203, D5 C2C12 myotubes *p* = 0.0480, **p* <0.05. **e.** D5 C2C12 myotubes were treated with 1 μM oxandrolone (blue) for 24 hours against an absolute ethanol (vehicle, black) to evaluate the mRNA levels via qPCR of target genes *Dok5*, *Igfbp5*, *Akap6*, *MyoG* and *Ddit4*. Data are shown as bar charts with scatter graphs that represent mean ± SEM error bars; n = 4 samples per group across two independent experiments. Two-way ANOVA followed by uncorrected Fisher’s least significant difference (LSD), F = 0.7013.

**Figure S11. 0.5% carboxymethyl cellulose vehicle treatment had no effect on survival or phenotype in *Smn^2B/-^* SMA and *Smn^2B/+^* healthy mice.**

All treated animals received a daily dose of 0.5% carboxymethyl cellulose (CMC) vehicle by gavage starting at P8**. a.** Survival curves of untreated (n = 15, black) and 0.5% CMC vehicle-treated (n = 13, violet) *Smn^2B/-^* SMA mice. Kaplan-Meier survival curve shown with log rank (Mantel-Cox) test, ns = not significant, *p* = 0.4222. **b.** Daily weights of untreated (n = 15, black) and 0.5% CMC vehicle-treated (n = 13, violet) *Smn^2B-/-^* SMA mice. Data represented as mean ± SEM error bars; Two-way ANOVA followed by a Sidak’s multiple comparison test, F = 335 df = 511 **c.** Daily righting reflex test for motor function activity up to a 30 second maximum time point in untreated (n = 15, black) and 0.5% CMC vehicle-treated (n = 13, violet) *Smn^2B/-^* SMA mice. Data are shown as bar chart with mean ± SEM error bars; unpaired T-test, ns = not significant, *p* = 0.5602**. d.** Survival curves of untreated (n = 21, black) and 0.5% CMC vehicle-treated (n = 16, violet) *Smn^2B/+^* healthy mice. Kaplan-Meier survival curve shown with log rank (Mantel-Cox) test, ns = not significant, *p* > 0.9999. **e.** Daily weights of untreated (n = 21, black) and 0.5% CMC vehicle-treated (n = 16, violet) *Smn^2B/+^* healthy mice. Data represented as mean ± SEM error bars; Two-way ANOVA followed by a Sidak’s multiple comparison test, F = 377.3, df = 724. **f.** Daily righting reflex test for motor function activity up to a 30 second maximum time point untreated (n = 21, black) and 0.5% CMC vehicle-treated (n = 16, violet) *Smn^2B/+^* healthy mice. Data are shown as bar chart with mean ± SEM error bars; unpaired T-test, ns = not significant, *p* = 0.9638**.**

**Figure S12.** **4 mg/kg/day oxandrolone decreased bodyweight in healthy *Smn^2B/+^* mice.**

All treated animals received a daily dose of oxandrolone (4 mg/kg/day, suspended in 0.5% CMC) by gavage starting at P8. **a.** Survival curves of untreated (n = 21, black) and 4 mg/kg/day oxandrolone-treated (n = 10, orange) *Smn^2B/+^* healthy mice. Kaplan-Meier survival curve shown with log rank (Mantel-Cox) test, ns = not significant, *p* > 0.9999. **b.** Daily weights of untreated (n = 21, black) and 4 mg/kg/day oxandrolone-treated (n = 10, orange) *Smn^2B/+^* healthy mice. Data represented as mean ± SEM error bars; Two-way ANOVA followed by a Sidak’s multiple comparison test, F = 395.5, df = 598, **p* < 0.05, ***p* < 0.01, ****p* < 0.001, *****p* < 0.0001. **c.** Daily righting reflex test for motor function activity up to a 30 second maximum time point in untreated (n = 21, black) and 4 mg/kg/day oxandrolone-treated (n = 10, orange) *Smn^2B/+^* healthy mice. Data are shown as bar chart with mean ± SEM error bars; unpaired T-test, ns = not significant, *p* = 0.7865**.**

**SUPPLEMENTARY TABLES**

**Table S1. RNA sequencing sample groups.**

**Table S2. Murine primers for qPCR.**

**Table S3. Significant differentially expressed genes (Log2FC > 0.6; FDR < 0.05) in skeletal muscle (triceps) between P7 untreated *Smn^-/-^;SMN2* SMA and *Smn^+/-^;SMN2* healthy mice.**

**Table S4. Significant differentially expressed genes (Log2FC > 0.6; FDR < 0.05) in skeletal muscle (triceps) between P7 prednisolone-treated vs untreated *Smn^-/-^;SMN2* SMA mice.**

**Table S5. Raw Counts for significant differentially expressed genes in skeletal muscle (triceps) between P7 prednisolone-treated vs untreated *Smn^-/-^;SMN2* SMA and untreated *Smn^+/-^;SMN2* healthy mice.**

**Table S6. Complete list of significant Gene Ontology (GO) Term (Biological Processes) for P7 prednisolone-treated vs untreated *Smn^-/-^;SMN2* SMA mice.**

**Table S7. Complete list of significant Gene Ontology (GO) Term (Molecular Functions) for P7 prednisolone-treated vs untreated *Smn^-/-^;SMN2* SMA mice.**

**Table S8. Complete list of significant Gene Ontology (GO) Term (Cell Components) for P7 prednisolone-treated vs untreated *Smn^-/-^;SMN2* SMA mice.**

**Table S9. List of drugs identified in iPathwayGuide from KEGGS drug database that target significantly impacted prednisolone pathways.**

**Table S10. List of predicted upstream regulators from P7 prednisolone-treated vs untreated *Smn^-/-^;SMN2* SMA mice used in DGIdb v.3. gene-drug target search.**

**Table S11. List of predicted agonist drugs from DGIdb v.3. for targeting upregulated upstream regulators.**

**Table S12. List of predicted antagonist drugs from DGIdb v.3. for targeting downregulated upstream regulators.**

**Table S13. Docking scores (kcal/mol) of the selected drugs with their predicted gene targets.**
