# Supplementary material for: A transcriptomics-based drug repositioning approach to identify drugs with similar activities for the treatment of muscle pathologies in spinal muscular atrophy (SMA) models
Source: Hum Mol Genet. 2023 Nov 8;33(5):400–25. doi: 10.1093/hmg/ddad192 (PMC10877467; doi:10.1093/hmg/ddad192)
Supplement: Supplementary_data_ddad192 [file supplementary_data_ddad192.zip › Supplementary_data_ddad192/Table S13.docx]

| **Table S13. Docking scores (kcal/mol) of the predicted drug-target interactions** | | |
| --- | --- | --- |
| **Drug** | **Protein** | **Docking score** |
| Metformin | Prkag1 | -3.0 |
| Acamprosate | Grin1 | -5.0 |
| Acamprosate | Grin2a | -4.0 |
| Acitretin | RarB | -16.2 |
| Oxandrolone | Ar | -11.7 |
| Nandrolone | Ar | -11.4 |
| Progesterone | Esr1 | -6.4 |
| Tibolone | Esr1 | -8.8 |
| Cannabidiol | Cnr1 | -10.8 |
| Celecoxib | Pdpk1 | -4.5 |
