# Supplementary figures and images for: A transcriptomics-based drug repositioning approach to identify drugs with similar activities for the treatment of muscle pathologies in spinal muscular atrophy (SMA) models
Source: Hum Mol Genet. 2023 Nov 8;33(5):400–25. doi: 10.1093/hmg/ddad192 (PMC10877467; doi:10.1093/hmg/ddad192)

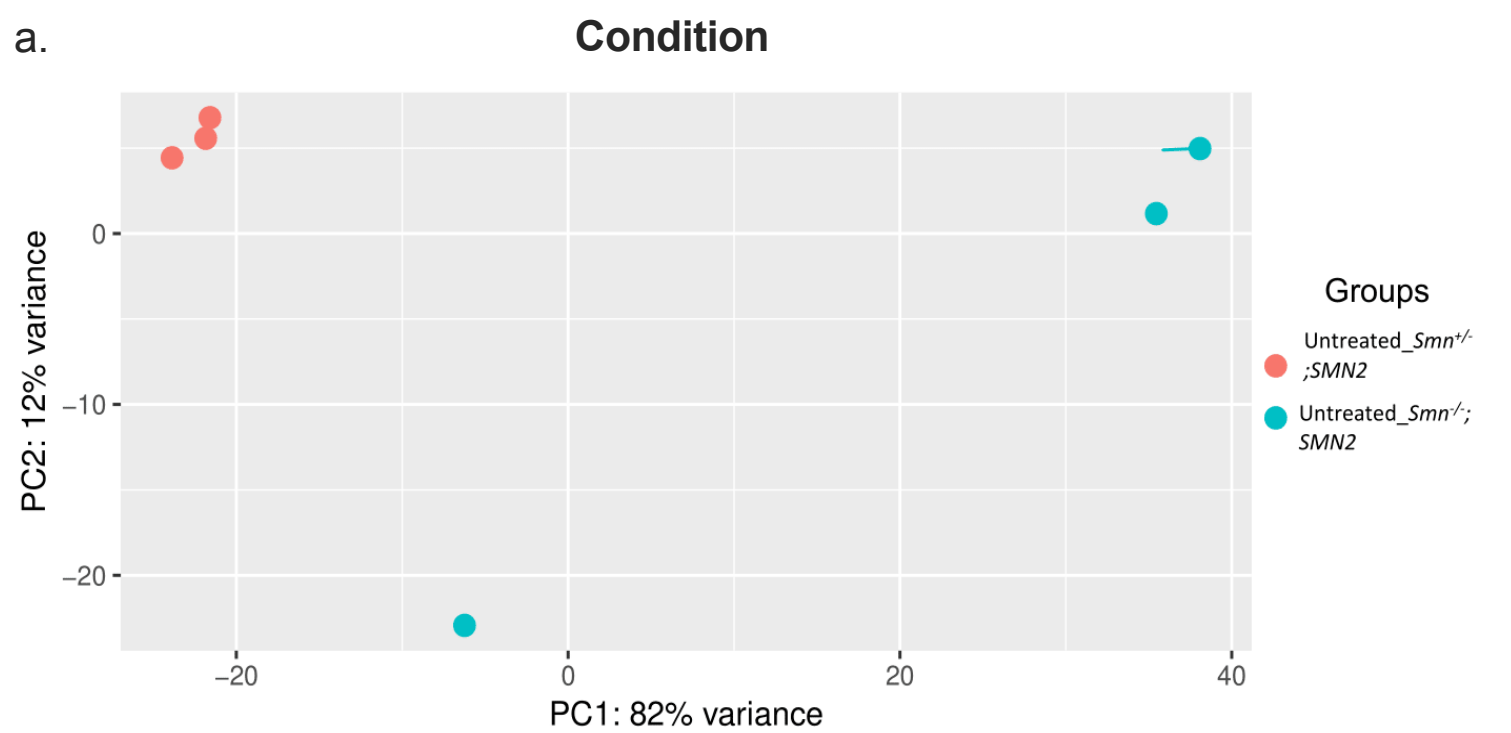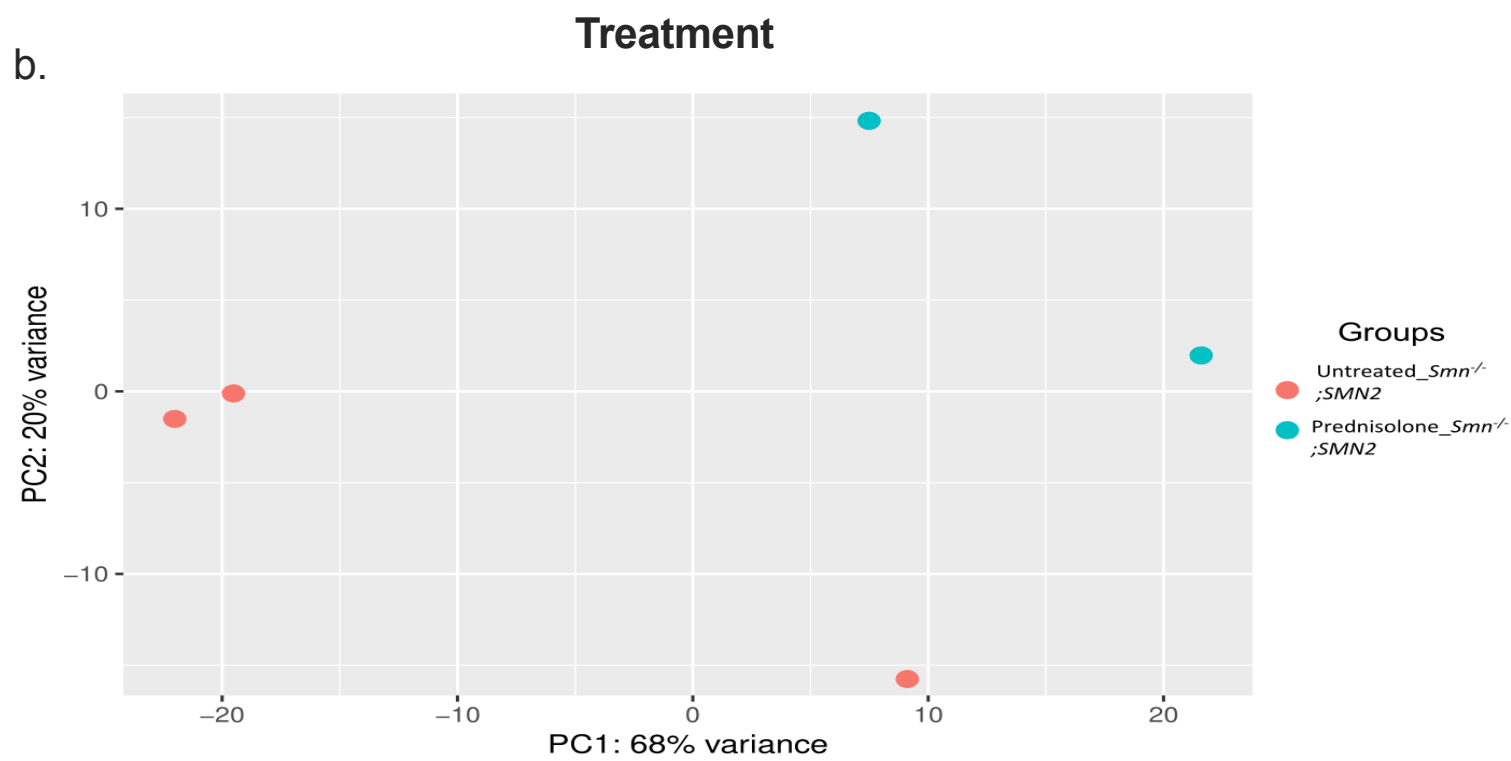

Supplement: Supplementary_data_ddad192 [file supplementary_data_ddad192.zip › Supplementary_data_ddad192/Figure S.1..pdf]

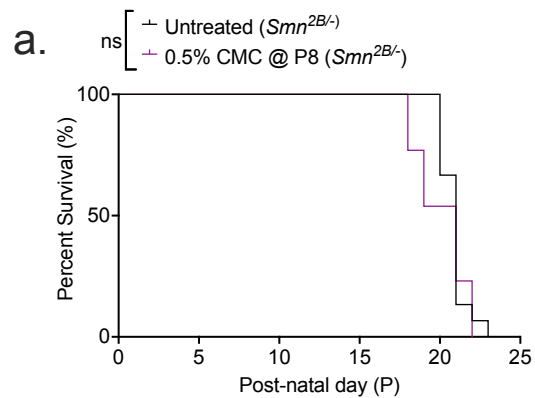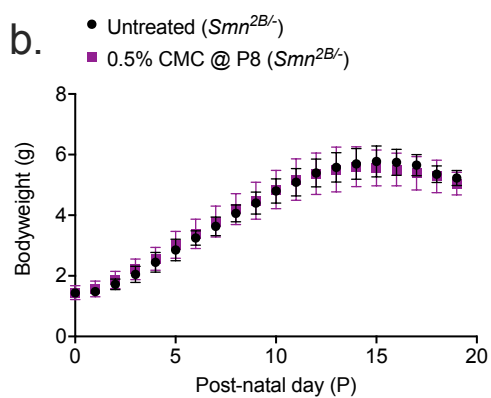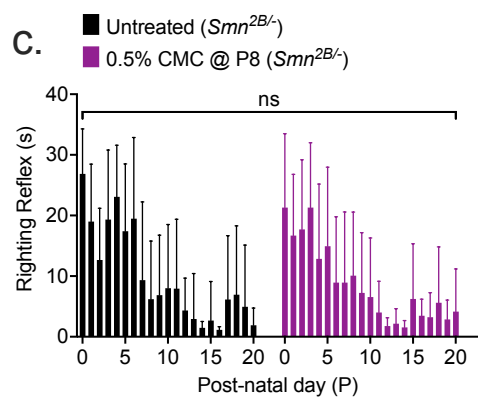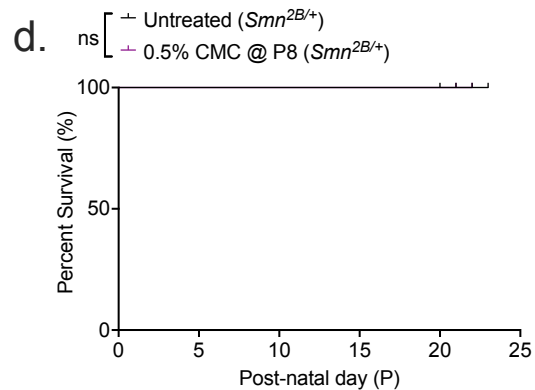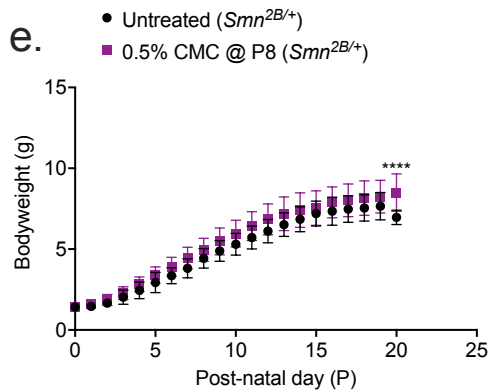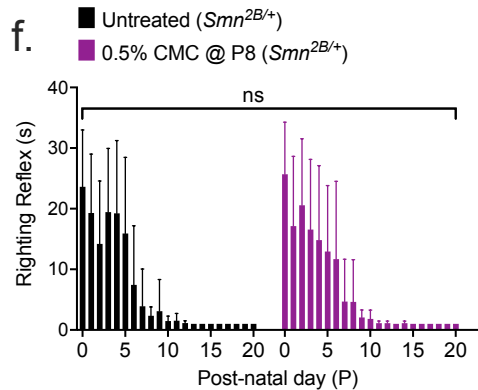

Supplement: Supplementary_data_ddad192 [file supplementary_data_ddad192.zip › Supplementary_data_ddad192/Figure S.10..pdf]

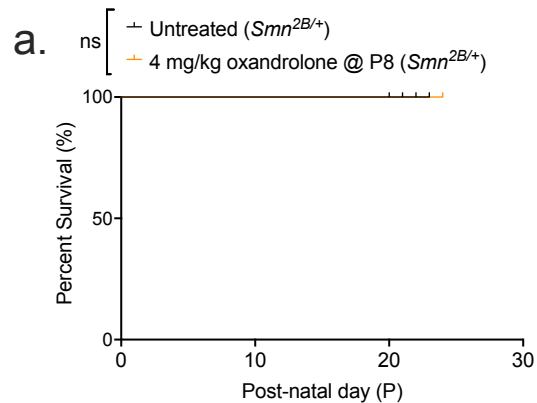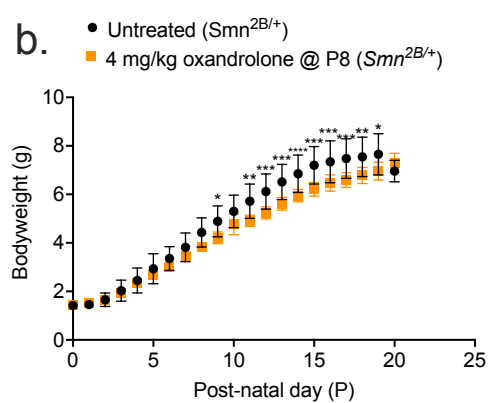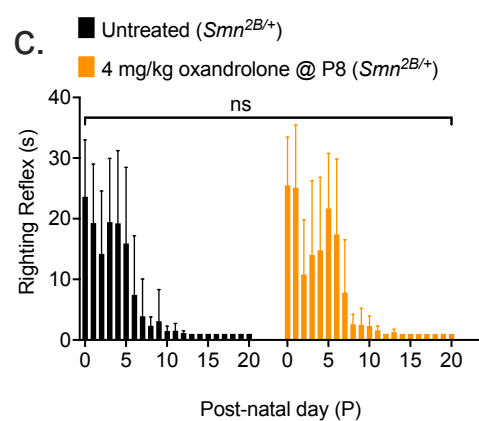

Supplement: Supplementary_data_ddad192 [file supplementary_data_ddad192.zip › Supplementary_data_ddad192/Figure S.11..pdf]

a.

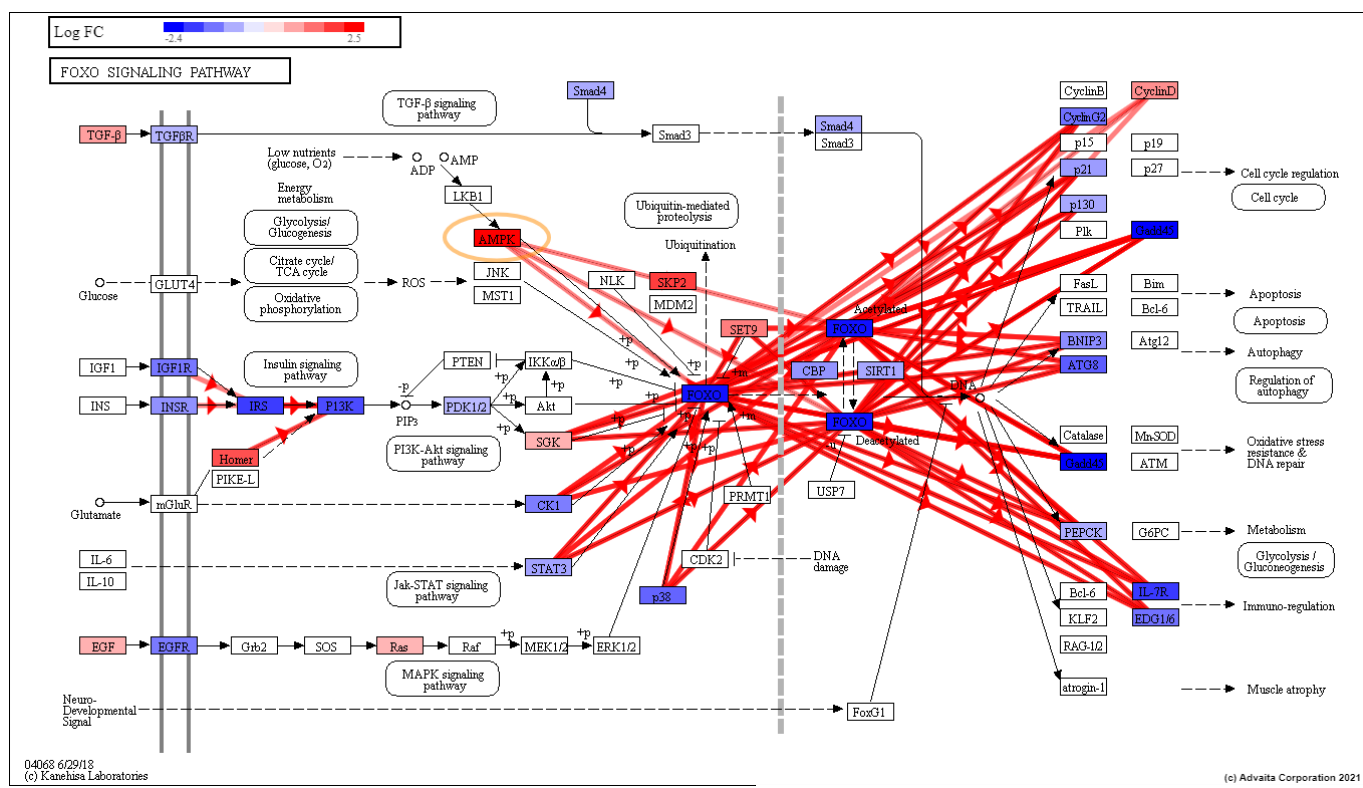

b.

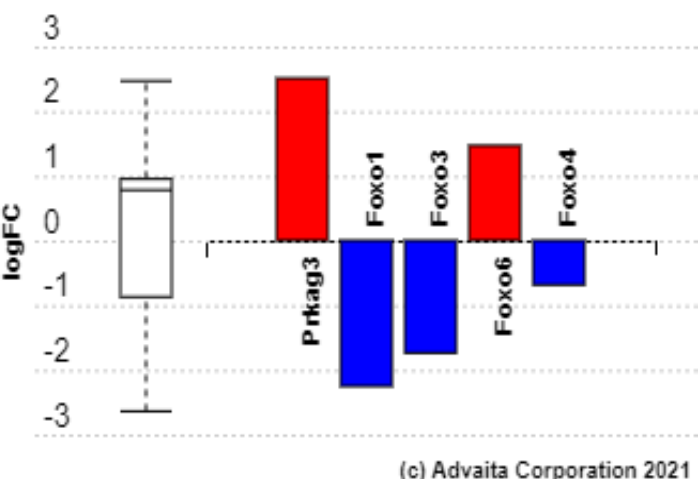

c.

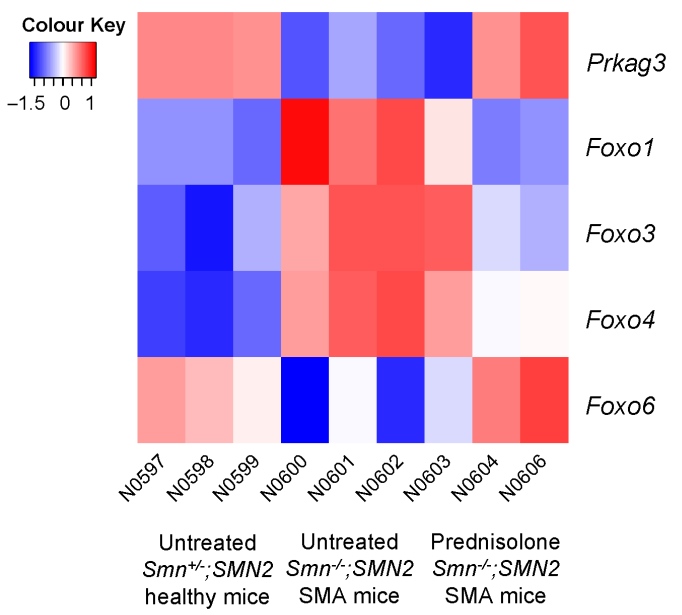

Supplement: Supplementary_data_ddad192 [file supplementary_data_ddad192.zip › Supplementary_data_ddad192/Figure S.2..pdf]

a.

C2C12 myoblast *Smn* KD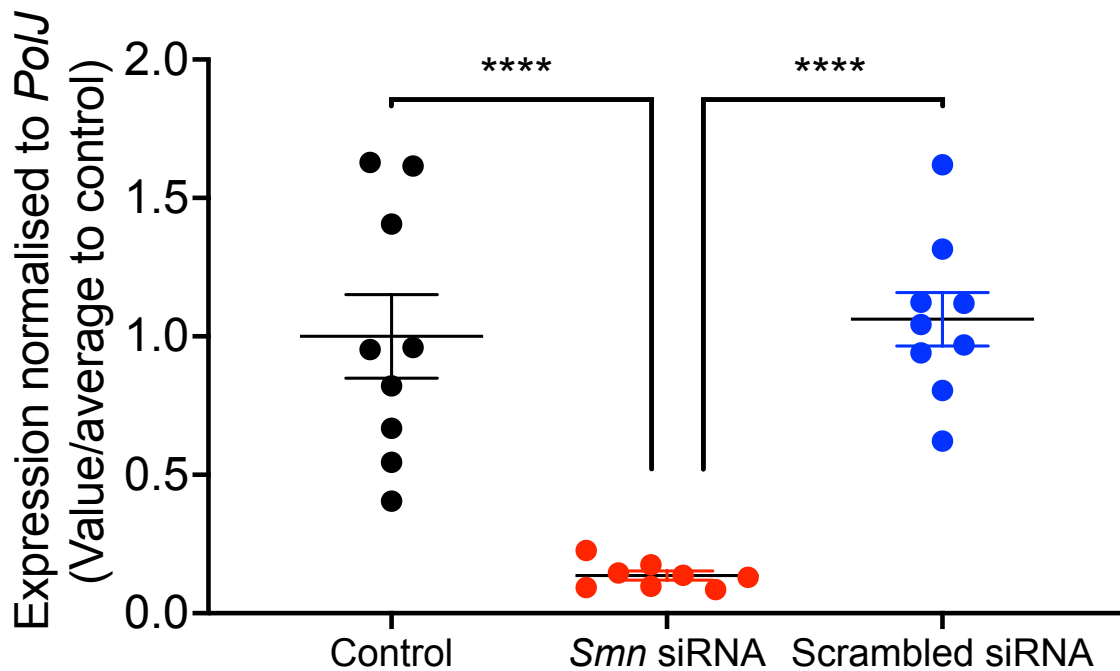

b.

D8 C2C12 myotube *Smn* KD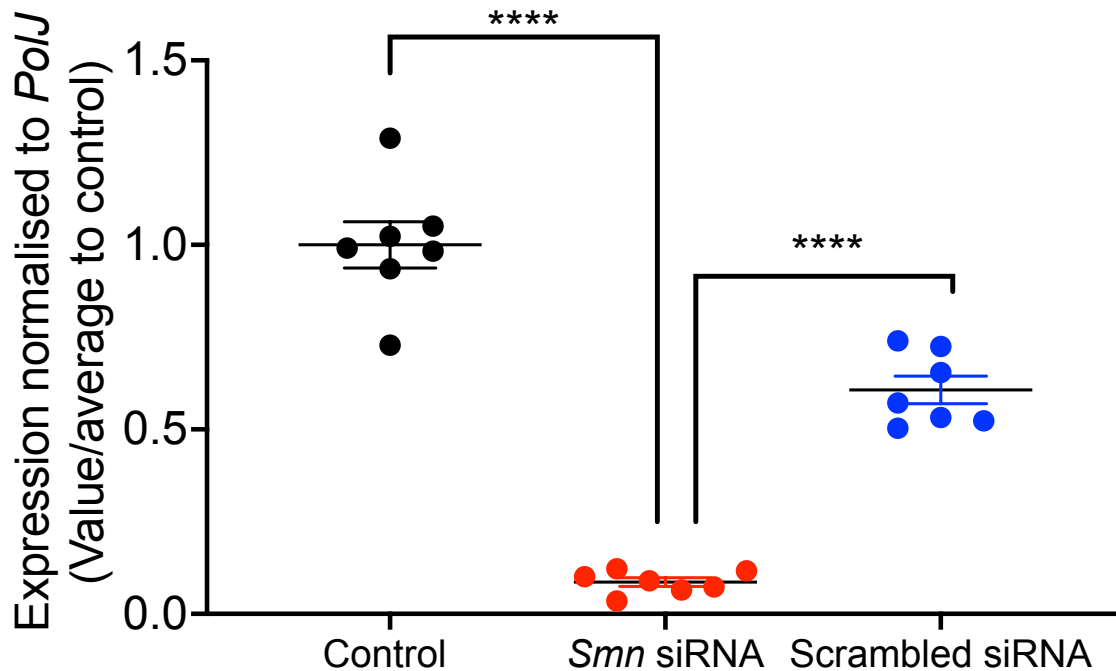

Supplement: Supplementary_data_ddad192 [file supplementary_data_ddad192.zip › Supplementary_data_ddad192/Figure S.3..pdf]

a. C2C12 myoblast Metformin gene-dose response

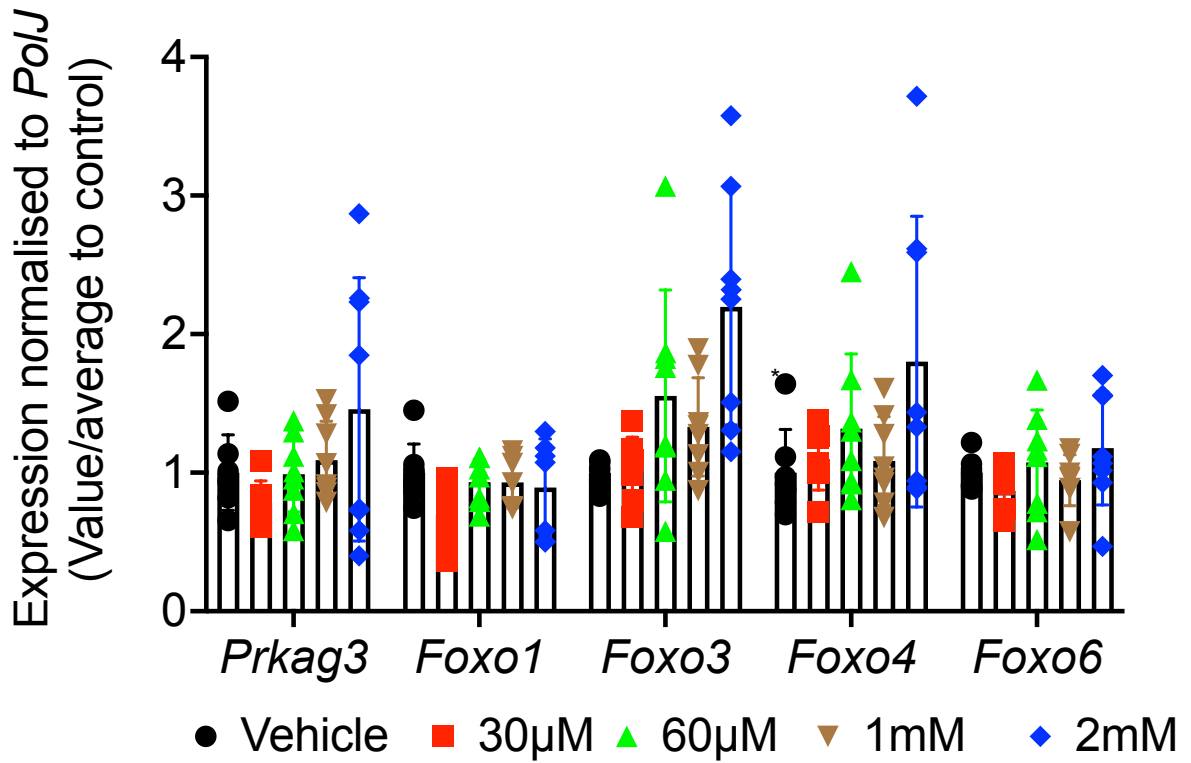

b. D8 C2C12 myotube Metformin gene-dose response

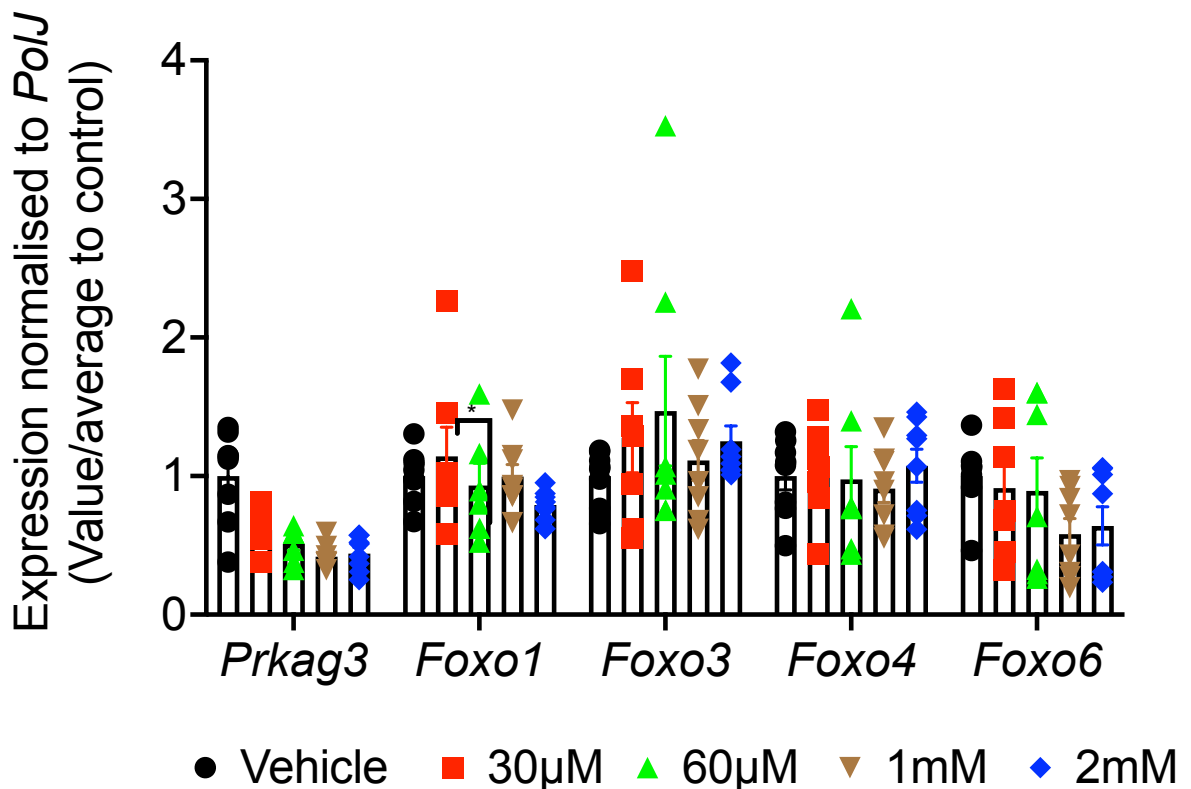

Supplement: Supplementary_data_ddad192 [file supplementary_data_ddad192.zip › Supplementary_data_ddad192/Figure S.4..pdf]

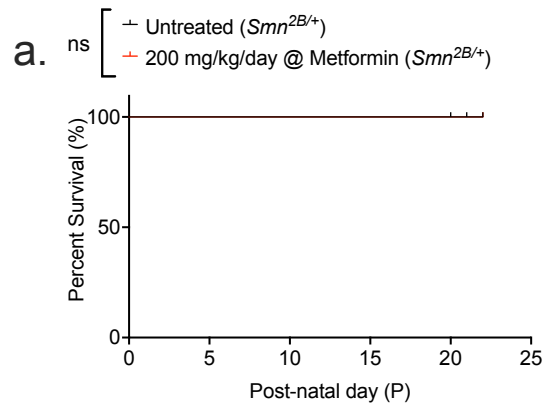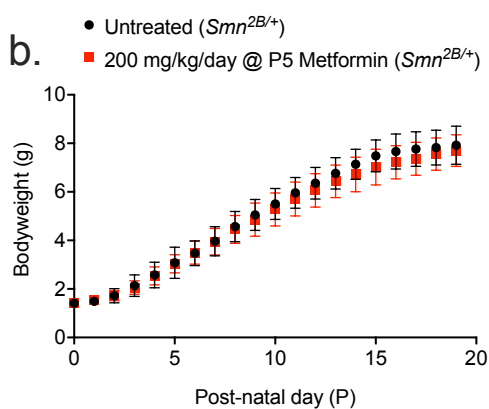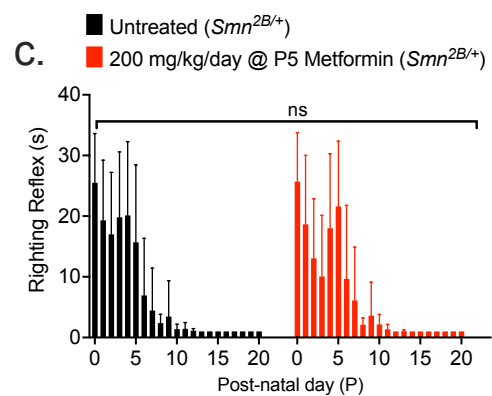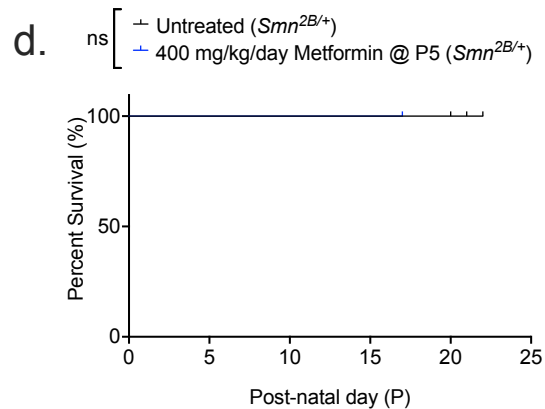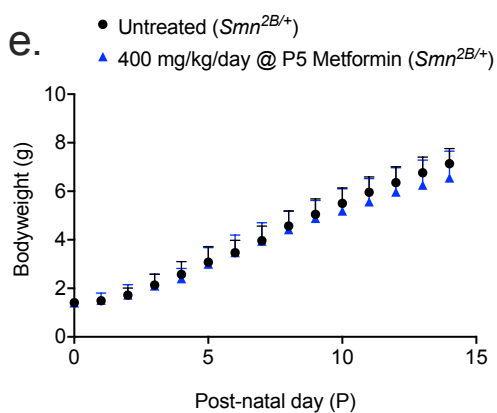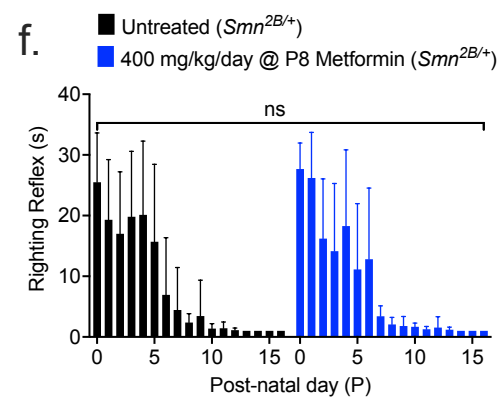

Supplement: Supplementary_data_ddad192 [file supplementary_data_ddad192.zip › Supplementary_data_ddad192/Figure S.6..pdf]

a.

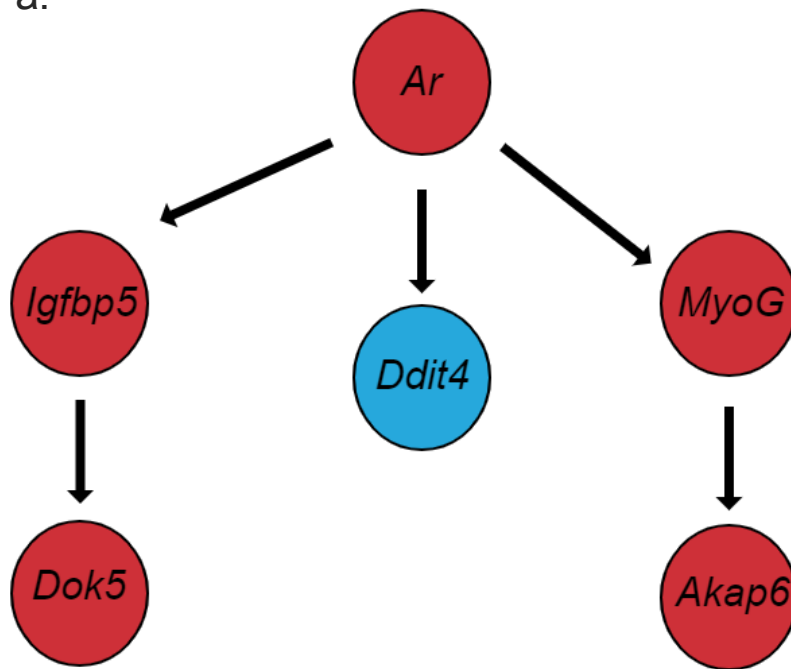

b.

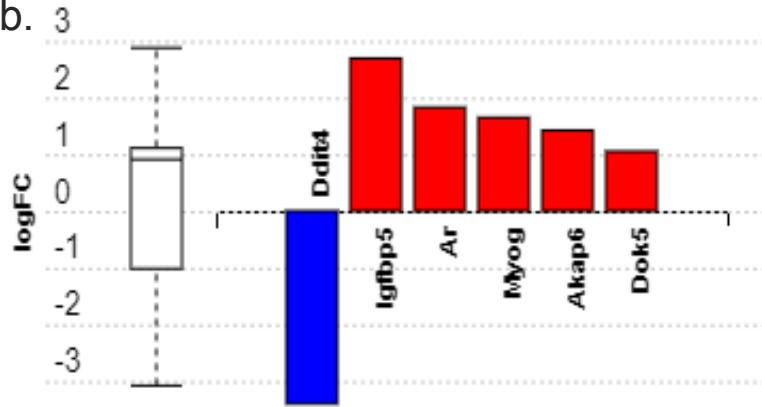

(c) Advaita Corporation 2021

c.

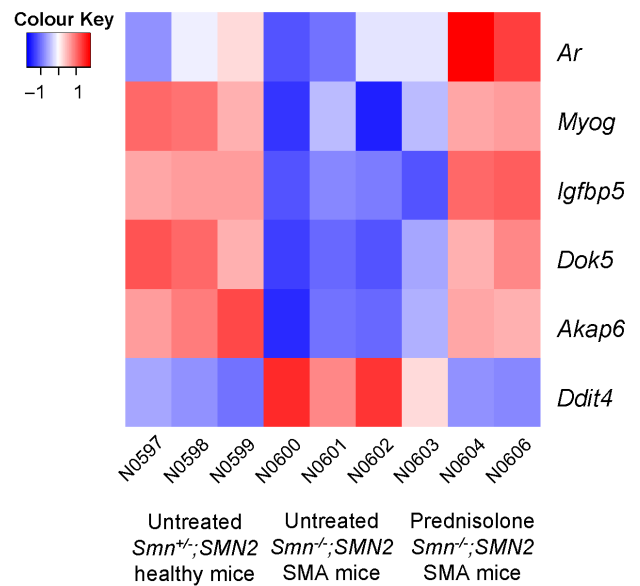

Supplement: Supplementary_data_ddad192 [file supplementary_data_ddad192.zip › Supplementary_data_ddad192/Figure S.7..pdf]

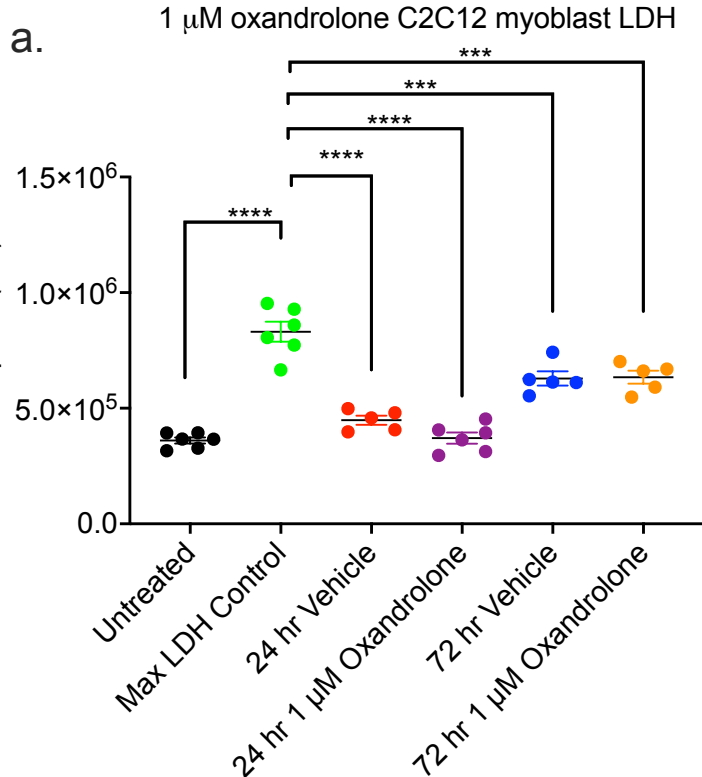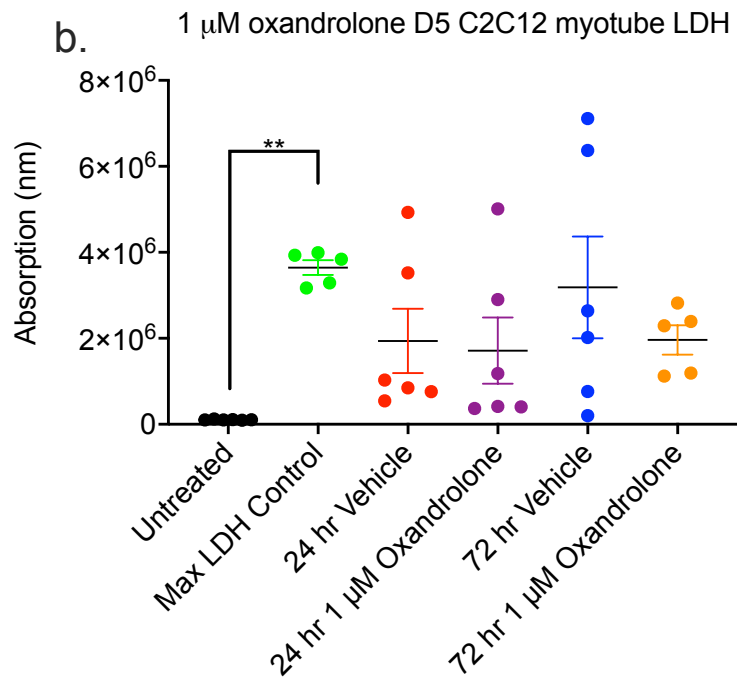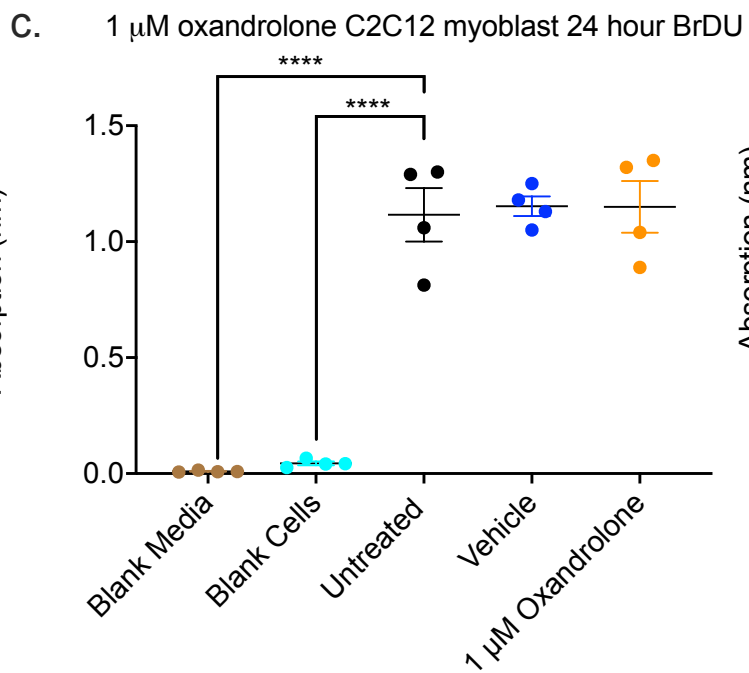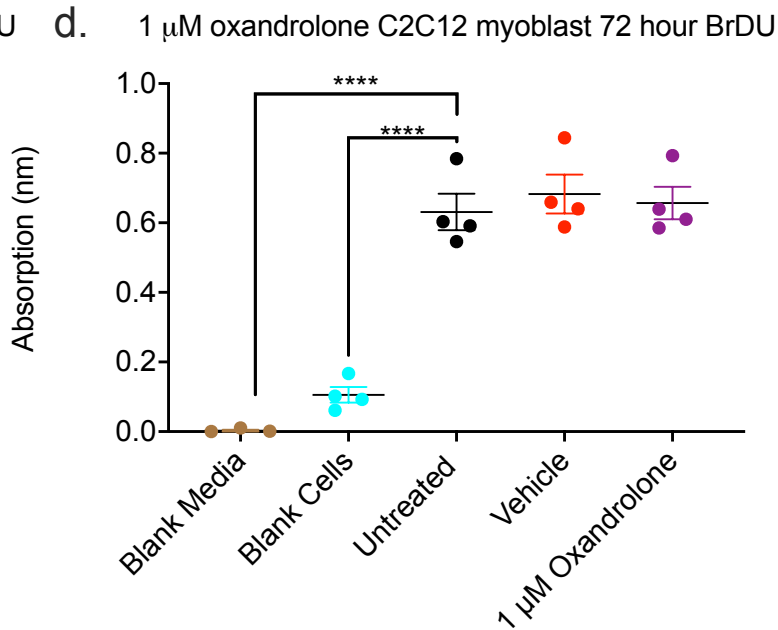

Supplement: Supplementary_data_ddad192 [file supplementary_data_ddad192.zip › Supplementary_data_ddad192/Figure S.8..pdf]

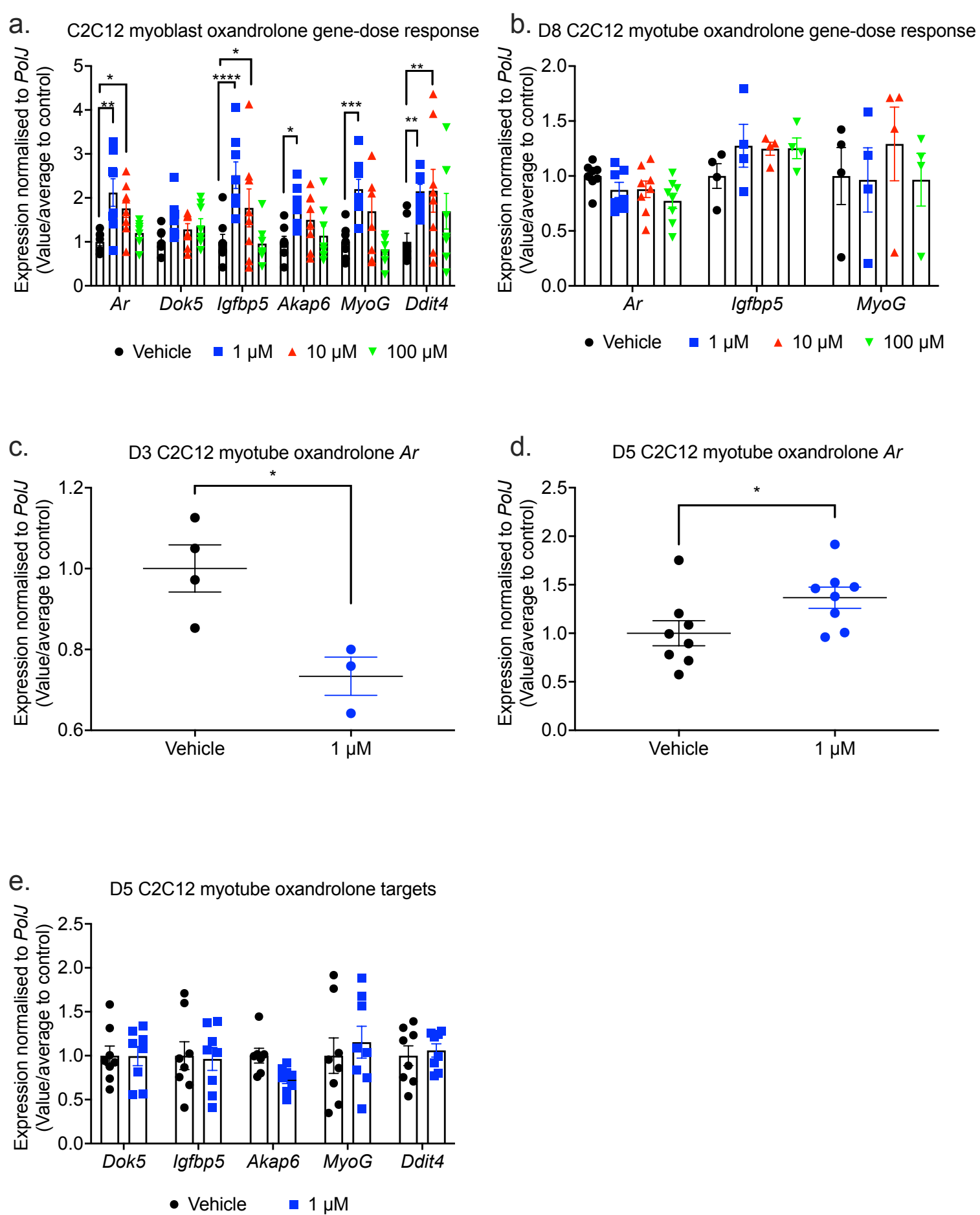

Supplement: Supplementary_data_ddad192 [file supplementary_data_ddad192.zip › Supplementary_data_ddad192/Figure S.9..pdf]
